# Simultaneous Detection of Carbon Monoxide and Viscosity Changes in Cells

**DOI:** 10.1002/anie.202008224

**Published:** 2020-09-17

**Authors:** Jonathan A. Robson, Markéta Kubánková, Tamzin Bond, Rian A. Hendley, Andrew J. P. White, Marina K. Kuimova, James D. E. T. Wilton‐Ely

**Affiliations:** ^1^ Department of Chemistry Molecular Sciences Research Hub Imperial College London White City Campus London W12 0BZ UK

**Keywords:** endogenous CO, fluorescence, ruthenium, sensing, viscosity

## Abstract

A new family of robust, non‐toxic, water‐compatible ruthenium(II) vinyl probes allows the rapid, selective and sensitive detection of endogenous carbon monoxide (CO) in live mammalian cells under normoxic and hypoxic conditions. Uniquely, these probes incorporate a viscosity‐sensitive BODIPY fluorophore that allows the measurement of microscopic viscosity in live cells via fluorescence lifetime imaging microscopy (FLIM) while also monitoring CO levels. This is the first example of a probe that can simultaneously detect CO alongside small viscosity changes in organelles of live cells.

## Graphical abstract

Carbon monoxide (CO) is produced in mammals and regulates many critical cellular functions, including the response to disease. A ruthenium(II) vinyl probe is presented that allows rapid, selective and sensitive detection of endogenous CO in live mammalian cells under normoxic and hypoxic conditions. By exploiting fluorescence lifetime imaging microscopy, the probe can also measure microscopic viscosity in live cells at the same time.

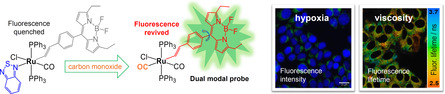

Carbon monoxide (CO) has long been associated with its toxicity, however, this colourless and odourless gas also plays a key role in cellular messaging.^[1]^ Its anti‐inflammatory, anti‐proliferative, anti‐apoptotic and anti‐coagulative properties are now recognised. Intriguingly, under pathophysiological conditions (e.g., inflammation) CO production in cells increases,^[2]^ and the real‐time monitoring of these changes could potentially provide diagnostic information. Haemoglobin is required as a substrate for CO production in vivo and the haem oxygenases (HO‐1 and HO‐2) play a key role in the generation of this gas in mammals. Emerging evidence suggests that the increased generation of HO‐derived CO plays a critical role in the resolution of inflammatory processes and alleviation of cardiovascular disorders,^[3]^ which has driven interest in CO‐releasing molecules (CORMs) for therapy.^[4]^

A major obstacle is the lack of effective methods to track CO in biological systems in real time.^[5]^ Imaging with emissive probes has emerged as one of the most powerful techniques to detect biologically important molecules. However, designing selective CO probes for the cellular environment is challenging, with its wide range of reactive species and variation in pH. He^[6]^ and Chang^[7]^ pioneered two very different approaches for the fluorogenic sensing of CO in cells. The latter study formed the basis for the majority of subsequent reports that used palladium compounds, which release the fluorophore either through carbonylation or protonolysis.[^8^, ^9^, ^10^] These processes are slow at 37 °C, leading to long response times (commonly >40 min), and use potentially cytotoxic, non‐ligated heavy metal salts or require addition of significant amounts of organic co‐solvents.[^7^, ^8^, ^9^, ^10^, ^11^] There is a pressing need for sensitive, selective, rapid and reliable fluorescent CO probes which overcome the limitations listed above.

Another key parameter of intracellular environments is microscopic viscosity (microviscosity). Abnormalities in viscosity are linked to disease and malfunction^[12]^ through the disruption of diffusion‐controlled cell signalling and transport.^[13]^ Microscopic viscosity in single live cells changes significantly as a result of production of reactive oxygen species (ROS).^[14]^ Therefore, increases in microviscosity could be correlated to CO production during inflammation and in cardiovascular disorders, which are both accompanied by oxidative stress. Furthermore, in mitochondria, CO is known to play a role in the modulation of ROS formation. This context led us to design a probe for the simultaneous detection of CO and changes in local viscosity.

Molecular rotors are fluorophores that allow quantitative mapping and real‐time monitoring of microviscosity changes within a live cell.[^15^, ^16^, ^17^] The non‐radiative decay of a fluorescent excited state of a rotor is influenced by viscosity changes.[^16^, ^17^] This sensitivity is due to a conformational change, which occurs after excitation, with a viscosity‐dependent rate. Intramolecular rotation is impeded in high viscosity environments, causing higher quantum yields and longer fluorescence lifetimes.[^16^, ^17^] Fluorescence lifetime imaging microscopy (FLIM) of molecular rotors allows viscosity mapping by spatially resolving the fluorescence decays of the molecular rotors.[^13^, ^14b^, ^14c^, ^17^, ^18^] Boron dipyrromethene (BODIPY) molecules have emerged as robust and biocompatible rotors with a wide dynamic range of fluorescence lifetimes, from 100 ps to 6 ns, corresponding to a biologically relevant viscosity range of 1 to 5000 cP (centipoise).[^14b^, ^14c^, ^18^]

Our previous work on CO detection resulted in a new approach using divalent ruthenium vinyl complexes to deliver combined chromogenic and fluorogenic responses.^[19]^ Reaction of these compounds with CO results in bright, turn‐on emission of a conjugated fluorophore, providing high selectivity and sensitivity towards CO, as demonstrated in an in vivo model of inflammation.^[20]^ The probe design described herein incorporates a BODIPY‐based molecular rotor, which not only detects the binding of CO, but also enables the use of FLIM for viscosity monitoring.

Three BODIPY compounds functionalised with a terminal alkyne were prepared (**1**–**3**) and treated with [RuHCl(CO)(PPh_3_)_3_] and 2,1,3‐benzothiadiazole (BTD) to yield **4**–**6** (Figure 1) in 72–92 % yield (Supporting Information, Section S2). Due to the presence of the BODIPY fluorophore, excitation at 480 nm in dichloromethane led to weak emission (*Φ_f_*=0.009–0.10) at 543 nm. The synthesis routes are summarised in Figure S1–1 (Supporting Information).

**Figure 1:**
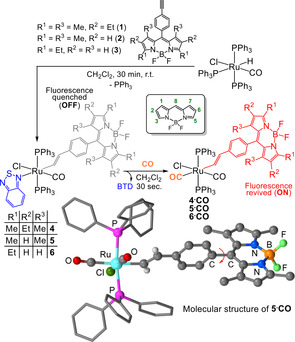
Synthesis of probes **4**–**6** and their reaction with CO.

The BTD ligand binds *trans* to the vinyl moiety and resists displacement by other species (even high concentrations of MeCN and DMSO).[^19^, ^20^] However, on exposure to CO, the BTD ligand is rapidly substituted to yield **4⋅CO**, **5⋅CO** (structurally characterised, Figure 1) and **6⋅CO**, which display significant fluorescence enhancement (Figures S4–2 to S4–6). The brightest emission (*Φ_f_*=0.77) was measured for **4⋅CO**, which displayed a 16‐fold revival (Figure S4–3) of the BODIPY fluorescence in pH 7.4, 25 mm phosphate‐buffered saline (PBS)‐DMSO (9:1 v/v) solution. The instantaneous reaction with CO is a key feature of these probes and a major advantage over Pd‐based systems, which require carbonylation or protonolysis to elicit the fluorescence response.[^5^, ^7^, ^8^, ^9^, ^10^]

Using CORMs to generate CO in aqueous solution (Supporting Information, Section S4),^[21]^ the performance of the probes was compared (Figure 2; Figure S4–7). Detection limits were 0.53 μm (≈15 ppb) for **4** and 0.36 μm (≈10 ppb) for **6**. Due to its superior fluorescence enhancement, complex **4** was chosen for sensing CO alone, while **6** was the only compound sensitive to both CO and viscosity and so was chosen for the combined sensing of both parameters.

**Figure 2:**
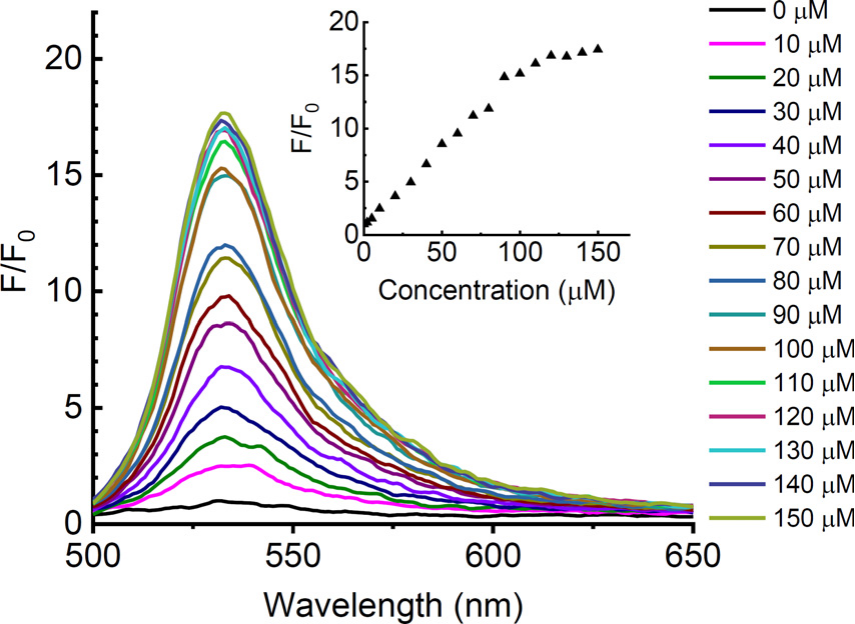
Fluorescence response of **6** (10 μm, pH 7.4, 25 mm PBS‐DMSO (9:1 v/v) solution) with 0–150 μm CORM‐2; *λ*_ex_=480 nm. Inset: the change in fluorescence intensity at 535 nm (Supporting Information, Figure S4–8). Key: fluorescence intensity without CO (F_0_), fluorescence intensity upon addition of CORM‐2 (F).

The selectivity for CO over other possible interferents was also measured (Supporting Information, Section S6). At pH 7.4, 25 mm PBS‐DMSO (9:1 v/v), all compounds were stable (as shown by NMR and fluorescence spectroscopy) towards N_2_, O_2_, CO_2_ and exposure to UV light for 24 h. The stability of the probes (10 μm in 1:9 v/v DMSO or acetone:PBS) was investigated with cellular species (bovine serum albumin, aspartic, glutamic, lipoic, citric, folic and ascorbic acids) and ROS (ClO^−^, HSO_3_^−^, SO_3_^2−^, H_2_O_2_), resulting in negligible changes to the fluorescence of the probes or their CO adducts (Supporting Information, Section S6). The pH of solution has been identified as an issue for stability in recent reports^[5]^ but negligible fluorescence changes were observed at pH 4 to 10 (Supporting Information, Figure S6–5) for **4** and **6**. Lastly, the probes were found to be non‐toxic to MCF‐7 cells at concentrations between 0–200 μm (Supporting Information, Section S7).

To investigate the CO‐sensing ability of **4** in cellulo, MCF‐7 cells were incubated with 10–100 μm CORM‐3 and stained with **4**. A clear increase in fluorescence intensity was observed (Figures 3 A,B; Supporting Information, Figure S5–4). Probe **4** was also successful in detecting CO generated through the addition of hemin as a substrate for HO‐1 catabolism (Figure 3 C). Quantification using ImageJ (Figure 3 D) revealed a 3‐fold increase in fluorescence intensity with CORM‐3 and more than a 2‐fold increase with hemin.

**Figure 3:**
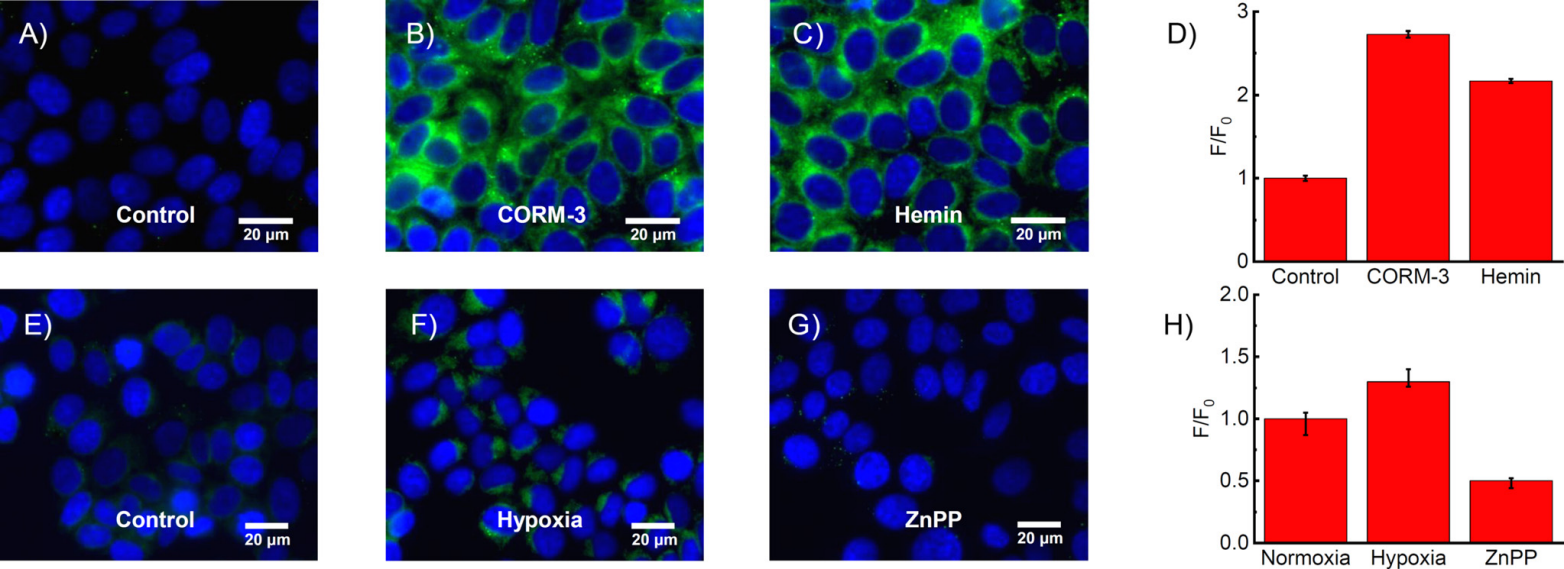
Fluorescence images of **4** (10 μm; green) and Hoechst 33342 (blue) in MCF‐7 cells: A) control untreated, B) 100 μm CORM‐3 for 30 min, C) 100 μm hemin for 5 h, E) control pre‐incubated in a normoxia incubator (37 °C, 5 % CO_2_, 95 % air) for 24 h, F) pre‐incubated in a hypoxia incubator (37 °C, 5 % CO_2_, 1 % O_2_/ 99 % N_2_) for 24 h, G) pre‐incubated with zinc protoporphyrin IX ZnPP (20 μm) in a hypoxia incubator for 12 h. Integrated change in fluorescence intensity for A–C is shown in D and for E–G in H. Key: fluorescence intensity in control or normoxia (F_0_), fluorescence intensity upon treatment (F). Data expressed as mean ±SEM of at least three independent experiments. Live cells (A–D), fixed cells (E–H). Scale bars=20 μm (Supporting Information, Figure S5–5). Key: instrument response function (IRF).

Haem oxygenase (HO‐1) plays a key role in the generation of cellular CO and is dysregulated in a wide variety of cancers.^[22]^ Hypoxic conditions can cause an increase in expression of HO‐1,^[23]^ thus probe **4** was tested against CO produced in hypoxia (24 h), in MCF‐7 cells, that were treated with **4**, then fixed to maintain the induced effect and compared to cells grown in a normoxia environment. Figures 3 E,F,H reveal the ability of the probe to detect the resulting endogenous CO. Zinc protoporphyrin IX (ZnPP) inhibits haem oxygenase and thus retards the degradation of haem to CO.^[24]^ Non‐fluorescent ZnPP (Supporting Information, Figure S6–10) was used as an additional control, preventing generation of CO (Supporting Information, Figure S5–2), as evidenced in Figure 3 G.

The simultaneous monitoring of carbon monoxide production and viscosity changes was investigated using probe **6**. A key design element of BODIPY molecular rotors is the viscosity‐dependent intramolecular twisting at the C8 position (Figure 1)^[13]^ and **6** is ideal due to the lack of substitution in the R^3^ position. Using time‐correlated single‐photon counting (TCSPC), the time‐resolved fluorescence decays of probe **6** were recorded in 0–100 % glycerol with methanol as a co‐solvent. Longer decays were observed as the glycerol percentage increased (Figure 4 A), confirming that the probe could successfully detect viscosity changes.

**Figure 4:**
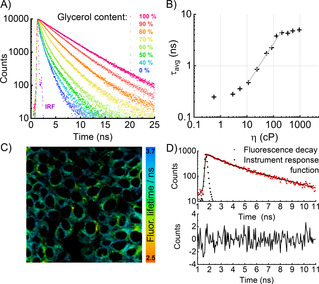
Fluorescence lifetime calibration of probe **6**. A) Time‐resolved fluorescence decays recorded in methanol‐glycerol mixtures of different viscosities. B) Double‐logarithmic calibration plot of average lifetime as a function of viscosity. The linear fit (grey) according to Equation 1 was applicable between 10 and 200 cP. C) FLIM of probe **6** (20 μm) in live MCF‐7 cells. D) Bi‐exponential fit of a typical fluorescence decay from a pixel in (C) (Supporting Information, Figure S8–7).

According to the Förster‐Hoffmann model,^[25]^ the plot of log *τ* (fluorescence lifetime, in ps) versus log *η* (viscosity, in cP) was fitted by a straight line (Figure 4 B):

(1) $$\log\;\tau {}_{avg}=-10.16+0.79\;\log\;\eta$$

Crucially, lifetime‐viscosity calibration plots for **6** and **6⋅CO** (obtained by treating **6** with CO) were almost identical (Figure S8–1), allowing viscosity measurements to be made independent of the presence of CO, while the higher fluorescence intensity of **6⋅CO** aided signal acquisition. Probe **6** was incubated in cells and FLIM was used to analyse the results (Figures 4 C,D), allowing straightforward spatiotemporal detection of the fluorescence lifetime, which correlates with viscosity according to Equation (1).

To test the simultaneous detection of CO and viscosity using **6**, MCF‐7 cells were incubated with CORM‐2 (0–200 μm) or hemin (0–100 μm). In both cases probe **6** exhibited a clear increase in fluorescence intensity (Figures 5 A–E), reporting generation of CO. At the same time, fluorescence decays were recorded to analyse microviscosity. No notable change in average lifetime was observed upon incubation with CORM‐2 (Figures 5 F–J). Using the fluorescence lifetime‐viscosity calibration of **6⋅CO**, the average lifetime of 3.3 ns corresponded to a viscosity of 133 cP, a typical value for lipid‐based intracellular organelles.[^13^, ^14b^, ^14c^, ^18^]

**Figure 5:**
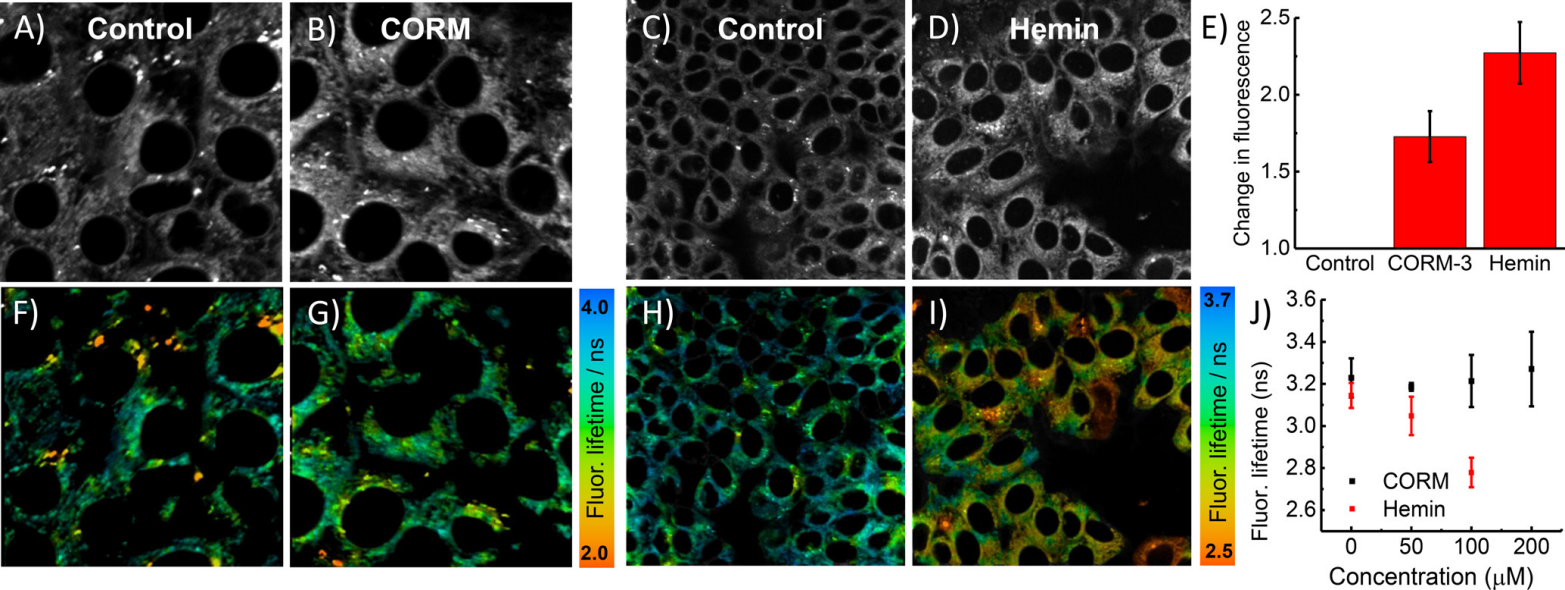
Fluorescence lifetime imaging of **6** (20 μm) in live MCF‐7 cells showing intensity (A–E) and lifetime (F–J); A,C) controls; B) incubated with 100 μm CORM‐2; D) incubated with 100 μm hemin. E) Change in fluorescence intensity of cells with CORM‐2 and hemin compared to controls. Underneath are the corresponding FLIM images. Data expressed as mean ±SEM of at least three independent experiments, and at least four FLIM images (Supporting Information, Figure S8–8).

Intriguingly, the shorter fluorescence lifetime observed upon incubation with hemin (Figures 5 I,J) suggested a decrease of intracellular viscosity (to 100 cP, 2.7 ns), which indicates that hemin and CORM‐2 treatments (both commonly used to generate CO in cells) do not result in the same cellular environment.

While CORM‐2 delivers CO into the cell and produces no changes in viscosity, hemin increases HO‐1 expression, modulating ROS inside the cell^[26]^ and our data reveals that it decreases viscosity. Furthermore, short‐lived ROS species may diffuse more easily through the cell due to the lower microviscosity induced by hemin.

Together with the fluorescence intensity data, these results represent the first simultaneous measurement of CO concentration and viscosity in cells. It is also the first example in which a molecular rotor is directly linked to a metal‐based chemosensor, while preserving its sensitivity to the viscosity of its environment, enabling dual viscosity and CO detection.

Many palladium‐based probes have been reported with greater sensitivity for CO than the ruthenium‐based system reported here.[^8^, ^10^] However, concerns remain over the toxicity of Pd^II^ salts^[11]^ in living systems and the slow response time caused by the need for diffusion controlled co‐location of three reaction partners (Pd salt, fluorophore and CO) for detection to occur. Our probe is the only competitive metal‐based system not based on palladium and successfully addresses both of these issues. It shows no cytotoxicity even up to 200 μm and instantaneous response to CO through its direct coordination to the metal. Such attributes make these systems highly effective, all‐round sensors for the detection of CO in the challenging environment of the cell. This is exemplified by the fact that probe **4** is able to detect endogenous CO generated through increased HO‐1 expression under hypoxic conditions. Most significantly, this design represents the first example of a dual modality CO and viscosity probe (**6**), allowing simultaneous measurement of CO through fluorescence intensity and viscosity through fluorescence lifetime. Our experiments show that an increase in HO‐1 expression leads to a decrease in cellular viscosity and this result may help to elucidate how HO‐1 modulates the activity of radical oxygen species. Our proof‐of‐concept biological studies provide an insight into the potential of these probes to characterise the mechanisms of delivery and signalling ability of CO within the body.

## Conflict of interest

The authors declare no conflict of interest.

## Supporting information

As a service to our authors and readers, this journal provides supporting information supplied by the authors. Such materials are peer reviewed and may be re‐organized for online delivery, but are not copy‐edited or typeset. Technical support issues arising from supporting information (other than missing files) should be addressed to the authors.

Supplementary
