# Supplementary material for: Simultaneous Detection of Carbon Monoxide and Viscosity Changes in Cells
Source: Angew Chem Int Ed Engl. 2020 Sep 17;59(48):21431–5. doi: 10.1002/anie.202008224 (PMC7756414; doi:10.1002/anie.202008224)
Supplement: Supplementary file 1 — Supplementary [file ANIE-59-21431-s001.pdf]

Angewandte Chemie
Eine Zeitschrift der Gesellschaft Deutscher Chemiker

Supporting Information

Simultaneous Detection of Carbon Monoxide and Viscosity Changes in Cells

Jonathan A. Robson, Markéta Kubánková, Tamzin Bond, Rian A. Hendley, Andrew J. P. White, Marina K. Kuimova, and James D. E. T. Wilton-Ely**

anie_202008224_sm_miscellaneous_information.pdf

S1 General considerations page S2
S2 Experimental page S6
S2.1 Synthetic procedure for preparation of ethynyl-BODIPY derivatives page S6
S2.2 Synthetic procedure for preparation of ruthenium BTD complexes page S13
S2.3 Synthetic procedure for preparation of ruthenium carbonyl adducts page S20
S3 Photophysical measurements of ethynyl-BODIPY compounds page S28
S4 Carbon monoxide detection in solution page S31
S5 Carbon monoxide detection in cells page S36
S6 Stability and competition measurements page S39
S7 Cytotoxicity assays page S45
S8 Viscosity measurements page S47
S9 Crystallography page S53
S9.1 The X-ray crystal structure of **5·CO** page S53
S10 References page S54

S1 General considerations

All the chemicals and solvents were purchased from Alfa-Aesar, Sigma-Aldrich and VWR and were used without further purification, unless otherwise stated. Solvents used for UV-Vis and fluorescence measurements were thoroughly degassed with nitrogen before use. All experiments and manipulations of compounds were conducted in air, unless otherwise specified. Petroleum ether is the fraction boiling in the 40–60 °C range. The commercially-available carbon monoxide lecture bottle (N3.7, purity 99.97%, with a CONCOA 302 – 2322 – CGA180 single-stage regulator) was purchased from CK Special Gases Limited and was used for isolation of all CO complexes. The gases used as interferents in this work were generated in situ according to standard protocols (see Appendix). These procedures provided materials of sufficient purity for synthetic and spectroscopic purposes. The complexes $[RuHCl(CO)(PPh_3)_3]$[S1] and 4,4-difluoro-8-(phenyl)-1,3,5,7-tetramethyl-4-bora-3a,4a-diaza-s-indancene[S2] were prepared according to published procedures. All moisture and oxygen sensitive compounds were prepared using standard Schlenk line and cannula techniques. Solvent mixtures are volume/volume mixtures. Solvents used in the reactions of oxygen and moisture sensitive compounds were dried and degassed according to standard techniques.

Waters LCT Premier ES-ToF (ESI) and a Micromass Autospec Premier (LSIMS) spectrometer were used for electrospray and high-resolution mass spectra (accurate mass mode). The HRMS was operated in positive mode under the following conditions: Gas1 35 psi, GS2 35, CUR 25, temperature 450 ºC, ion spray, voltage 5500 V.

Standard FT-IR spectra were measured using a Perkin Elmer Spectrum GX spectrometer and characteristic triphenylphosphine–associated infrared data are not reported. UV-Vis spectra was recorded with an Agilent 8453 UV-Vis spectrophotometer. Measurements were conducted at room temperature over a wavelength range of 190-1100 nm with a 1 nm wavelength step.

NMR spectroscopy was performed at 298 K using a Bruker AV400 or 500MHz spectrometer at room temperature in CD_2Cl_2 unless otherwise stated. ^{1}H NMR and ^{13}C NMR chemical shifts (δ) were referenced to the residual non-deuterated solvent signal and the ^{13}C signal of the deuterated solvent respectively. The deuterated solvents were all purchased from Sigma Aldrich. ^{31}P NMR chemical shifts were referenced externally to H_3PO_4 85% in H_2O respectively and were all proton decoupled. ^{19}F was referenced externally to trichlorofluoromethane and ^{11}B NMR was externally referenced to 15% BF_3•OEt_2 in $CDCl_3$.

Elemental analysis data were obtained from London Metropolitan University. The procedures given provide materials of sufficient purity for synthetic and spectroscopic purposes.

Fluorescence measurements were carried out on a FluoroMax4 spectrofluorimeter (Horiba Scientific) at room temperature. Fluorescence quantum yields Φ were determined by the comparative method using rhodamine G and cyanine-3 as fluorescence standards. Fluorescence quantum yield measurements were performed on a fluorimeter and UV/vis instrument. The slit was 2.5 nm for the excitation and 5 nm for the emission. Relative quantum efficiencies were obtained by comparing areas under the corrected emission spectrum**.** The following equation was used to calculate quantum yield:

$$\Phi_x = \Phi_{st}(I_x/I_{st})(A_{st}/A_x)\left({\eta^2}_x/{\eta^2}_{st}\right)$$

Where Φ_{st} is the reported quantum yield of the standard, I is the integrated emission spectrum, A is the absorbance at the excitation wavelength, and η is the refractive index of the solvents used. The subscript x denotes unknown and st denotes standard. $[Ru(bpy)_3]^{2+}$ ($\Phi = 0.018$ in acetonitrile) was used as a standard.

Solution Studies

Fluorescence titrations - A solution of the probe (10 µM in pH 7.4, 25 mM PBS–DMSO (9 : 1 v/v) was added to a cuvette with the fluorescence and absorbance recorded. CORM-2 (1 eq., aqueous CO generation stabilised by sodium dithionite (1 eq.))[S3] was dissolved in DMSO (2,000 µM) and 5 µL of this stock solution added to the cuvette after appropriate mixing (~1-2 min) the fluorescence spectra was recorded. Additions continued until 15 additions had been reached or fluorescence emission plateaued. Detection limit testing followed the same procedure but for much lower concentrations of CORM-2.

Selectivity testing – A solution of the probe (10 µM in pH 7.4, 25 mM PBS–DMSO (9:1 v/v) solution) was added to a cuvette with the fluorescence and absorbance recorded. A potential interferent (100 µM) was then added, after appropriate mixing (~15 min) the fluorescence spectra was recorded. With some interferents the fluorescence spectra was recorded every 5 min for 1 hr.

Stability testing - A solution of the probe (2 µM in pH 7.4, 25 mM PBS–DMSO (9:1 v/v) solution) was added to a cuvette with the fluorescence and absorbance recorded. The fluorescence spectra was then recorded at appropriate time points up to 24 hr.

The **fluorescence decays** of **6** and **6•CO** were measured using time-correlated single photon counting (TCSPC). The fluorescence decays were measured using the in-house TCSPC system composed of SPC-830 photon counting card (Becker & Hickl), DCC-100 detector control module (Becker & Hickl), PMC-100-1 PMT (Hamamatsu), Omni-l grating monochromator (LOT-Quantum Design), qpod cuvette holder (Quantum Northwest) and TC 125 Peltier temperature controller (Quantum Northwest). The probes were excited at 480 nm using the BDL488-SMN picosecond diode laser (Becker & Hickl) at 20 MHz frequency. The acquisition time window was 50 ns, with 4096 collection channels. The instrument response function, which was required to fit the decays, was measured by recording a scattering signal from a cuvette with Ludox solution.

Biological Studies

Unless stated otherwise microscopy experiments were carried out with live cells in FluoroBrite™ DMEM under normoxia conditions. Image acquisition occurred over 1 – 3 h, over this time no significant cellular death was observed. Microscope settings remained constant throughout data acquisition, therefore images taken of different wells could be compared.

CORM-3 experiments - The cells were seeded at a density of 1 x 10^{-4} cells/mL in 8-well plates (Corning® Costar®, Sigma-Aldrich) and grown for 24 h at 37 ºC in a 5% CO_2 incubator. A stock solution of CORM-3 in PBS was prepared and diluted in DMEM to reach a final concentration of (0 – 200 µM). After aspiration of cellular media, the CORM-3 solution (200 µL) was added to each well and incubated at 37 °C for 20 min. A stock solution of an appropriate CO probe in DMSO/acetone was prepared and diluted in DMEM to reach a final concentration of (10 – 20 µM). Final concentration did not contain more than 0.1% DMSO/acetone. The cellular media was once again aspirated and the probe solution (200 µL)

was added to each well and incubated at 37 °C for 10 - 20 min. After such time the media was aspirated, and cells were then washed with PBS (2 x 200 µL), FluoroBrite™ DMEM (200 µL) was then added to each well. The 8-well plate was then imaged using widefield, confocal or 2-photon microscopy.

Hemin experiments - The cells were seeded at a density of 1 x 10^{-4} cells/mL in 8-well plates (Corning® Costar®, Sigma-Aldrich) and grown for 24 h at 37 ºC in a 5% CO_2 incubator. A stock solution of hemin in 0.1 M NaOH was prepared and diluted in DMEM to reach a final concentration of (0 – 100 µM). After aspiration of cellular media, the CORM-3 solution (200 µL) was added to each well and incubated at 37 °C for 3 – 5 h. A stock solution of an appropriate CO probe in DMSO/acetone was prepared and diluted in DMEM to reach a final concentration of (10 – 20 µM). Final concentration did not contain more than 0.1% DMSO/acetone. The cellular media was once again aspirated and the probe solution (200 µL) was added to each well and incubated at 37 °C for 10 - 20 min. After such time the media was aspirated, and cells were then washed with PBS (2 x 200 µL), FluoroBrite™ DMEM (200 µL) was then added to each well. The 8-well plate was then imaged using widefield, confocal or 2-photon microscopy.

Zinc Protophophyrin IX experiments - The cells were seeded at a density of 1 x 10^{-4} cells/mL in 8-well plates (Corning® Costar®, Sigma-Aldrich) and grown for 24 h at 37 ºC in a 5% CO_2 incubator. A stock solution of ZnPP in DMSO was prepared and diluted in DMEM to reach a final concentration of (0 – 20 µM). After aspiration of cellular media, the CORM-3 solution (200 µL) was added to each well and incubated at 37 °C for 24 h. A stock solution of an appropriate CO probe in DMSO/acetone was prepared and diluted in DMEM to reach a final concentration of (10 – 20 µM). Final concentration did not contain more than 0.1% DMSO/acetone. The cellular media was once again aspirated and the probe solution (200 µL) was added to each well and incubated at 37 °C for 10 - 20 min. After such time the media was aspirated, and cells were then washed with PBS (2 x 200 µL), FluoroBrite™ DMEM (200 µL) was then added to each well. The 8-well plate was then imaged using widefield microscopy.

Cell lines - Human embryonic kidney cells (HEK 293) and Breast cancer (MCF-7) cells were kindly donated by Prof. Ed Tate from Imperial College London. The cells were routinely grown in a DMEM (Dulbecco's Modified Eagle Medium), high glucose + GlutaMAXTM medium containing 10% foetal calf serum (FCS), purchased from Gibco® by Life TechnologiesTM.

Cytotoxicity assays - once cells have reached confluence, they were rinsed with PBS, detached using trypsin, collected by centrifuge, seeded in a 96-well plates (Corning® Costar®, Sigma-Aldrich) to reach a cell density of 15000 cells per well and grown for 24 h at 37 ºC in a 5 % CO_2 incubator. For the MTT assays, the cells were then treated with various concentration of complex (0.5-250 µM) and incubated for 24 and 72h, cells treated with just medium served as a negative control. To measure the viability of the cell, the media was aspirated and replaced with (3-(4,5-dimethylthiazol-2-yl)-2,5-diphenyltetrazolium bromide) (MTT) in PBS (2 mg mL^{-1}) and incubated for 2h. The solution was then replaced with DMSO to dissolve the formazan crystals and the absorbance at 570 nm was measured on a 96-well plate reader (SpectraMax M2/M2e Microplate Reader from Molecular Devices). All experiments were done in quadruplicate and the relative cell viability was reported as a percentage relative to the control cells.

All **widefield images** of cells were carried out on WIDEFIELD WF3 Zeiss Cell Observer Live Cell Imaging System at FILM Imperial. The Facility for Imaging by Light

Microscopy (FILM) at Imperial College London is part-supported by funding from the Wellcome Trust (grant 104931/Z/14/Z) and BBSRC (grant BB/L015129/1).

For **time-resolved imaging experiments**, BODIPY was excited using a mode-locked femtosecond Ti:Sapphire laser (Coherent, Chameleon Vision II) using two photon excitation at 930 nm (140 fs pulse duration, 80 MHz). Fluorescence lifetime imaging microscopy (FLIM) was performed using a Leica, SP5 II confocal laser scanning microscope. Fluorescence was collected between 500 and 580 nm using a PMC-100-1 photomultiplier tube (Hamamatsu) and an SPC-830 single-photon counting card (Becker-Hickl). Live cell imaging was performed in glass bottom 8-well chamber slides (NuncTM, Lab-Tek, Thermofisher, MA. US.). Cells were seeded at approximately 1x10^4 cells per 0.7 cm^2 surface area chamber.

Image Processing - Microscopy images were processed and analysed using ImageJ software. Line segment analysis was performed using a macro produced by FILM Imperial. This analysis determined the fluorescence intensity of pixels crossing a minimum of ten cells, this allowed the average intensity of these cells to be calculated. The analysis was repeated for three separate experiments.

The scheme below summarises the compounds prepared (Section S2) and investigated (Sections S3-9) in this work:

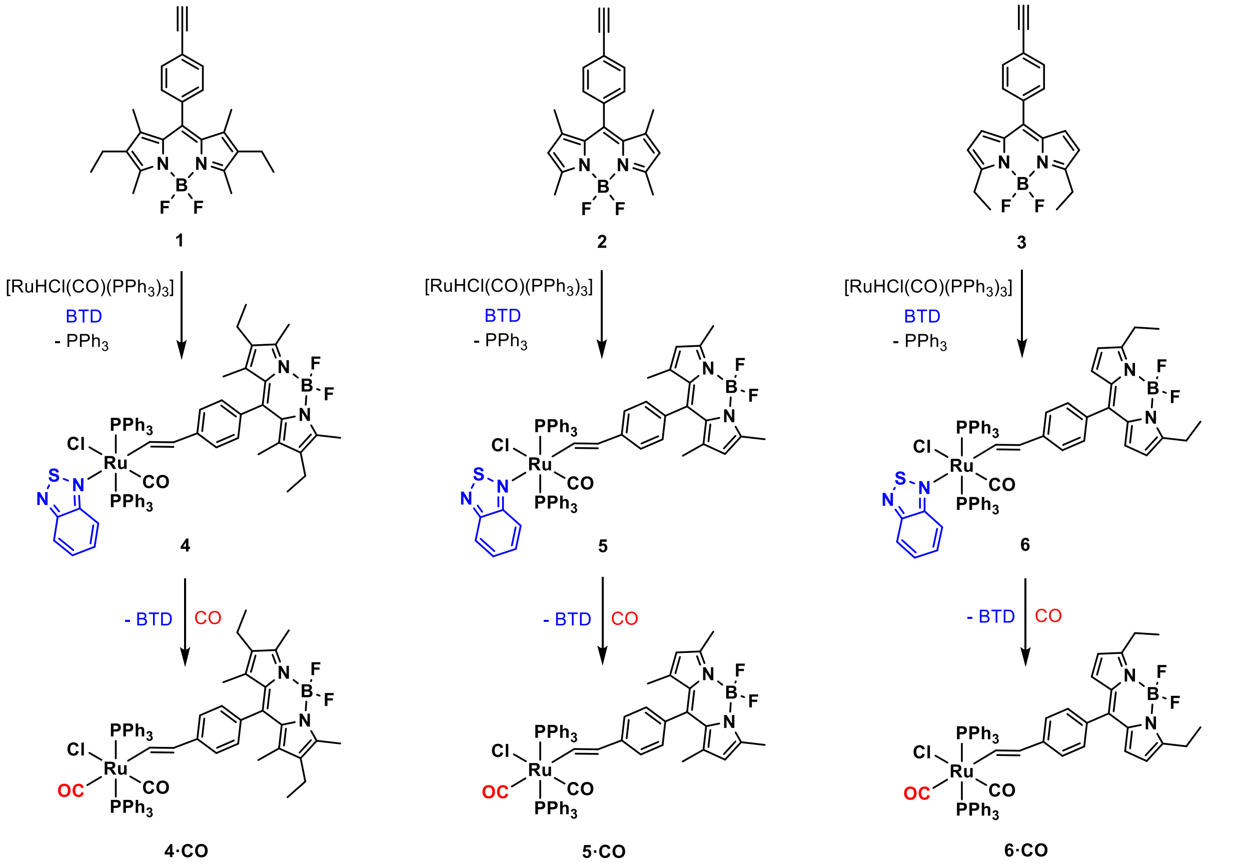

Figure S1-1. The ethynyl-BODIPY ligands, their respective Ru(II) probe complexes and the CO adducts formed. BTD = 2,1,3-benzothiadiazole.

S2 Experimental

S2.1 Synthetic procedure for preparation of ethynyl-BODIPY derivatives

Under an inert atmosphere, an alkylpyrrole (2.05 eq.) and 4-ethynylbenzaldehyde (0.60 g, 4.6 mmol, 1 eq.) were combined in anhydrous dichloromethane (300 mL). Trifluoroacetic acid (50 µL, catalytic quantity) was subsequently added and the reaction was stirred at room temperature for 48 h. A darkening of the yellow solution was observed. A solution of 2,3-dichloro-5,6-dicyano-*p*-benzoquinone (DDQ, 1 eq.) was added in dichloromethane and the solution was stirred for 50 min. Triethylamine (TEA) (15 eq.) and BF_3•OEt_2 (16 eq.) were added to the mixture and the reaction was stirred for a further 2 h. The reaction became purple on addition of BF_3•OEt_2. The solution was washed with water and dried over magnesium sulfate. After removal of the solvent, the solid was purified by chromatography on silica gel (dichloromethane – hexane 1:1 v/v). All products were isolated as orange/red powders.

Synthesis of ethynyl-BODIPY^{Me,Et,Me} (1)

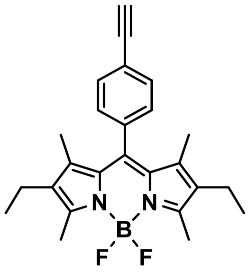

3-Ethyl-2,4-dimethylpyrrole (1.32 mL, 9.40 mmol) was treated with 4-ethynylbenzaldehyde (600 mg, 4.60 mmol) according to the general procedure[S4] giving a red powder (569 mg, 30%). IR (solid state): 1552, 1533, 1474, 1458, 1314, 1181, 1158, 1113, 1101, 1060, 1035, 1019, 971 cm^{-1}. ^1H NMR (400 MHz, CD_2Cl_2): δ 7.64, 7.28 (AB, 2 x 2H, J_{HH} = 8.1 Hz, C_6H_4), 3.21 (s, 1H, C≡CH), 2.55 (s, 6H, pyrrole-CH_3), 2.32 (q, 4H, J_{HH} = 7.6 Hz, pyrrole-CH_2), 1.32 (s, 6H, pyrrole-CH_3), 1.00 (t, J_{HH} = 7.5 Hz, 6H, pyrrole-CCH_3) ppm. $^{11}B\{^1H\}$ NMR (128 MHz, $CDCl_3$): δ 0.91 (t, J_{BF} = 33.6 Hz) ppm. $^{13}C\{^1H\}$ NMR (101 MHz, $CDCl_3$): δ 154.0, 138.1, 136.4, 132.7, 130.4, 128.5, 122.7, 109.9, 83.0, 78.3, 17.0, 14.5, 12.5 ppm. $^{19}F\{^1H\}$ NMR (377 MHz, $CDCl_3$): δ -145.79 (q, J_{FB} = 32.8 Hz) ppm. HRMS (ES^+-TOF) *m/z* 405.2325 $[M+H]^+$, calculated for $C_{25}H_{28}{}^{11}BF_2N_2$: 405.2314.[S5]

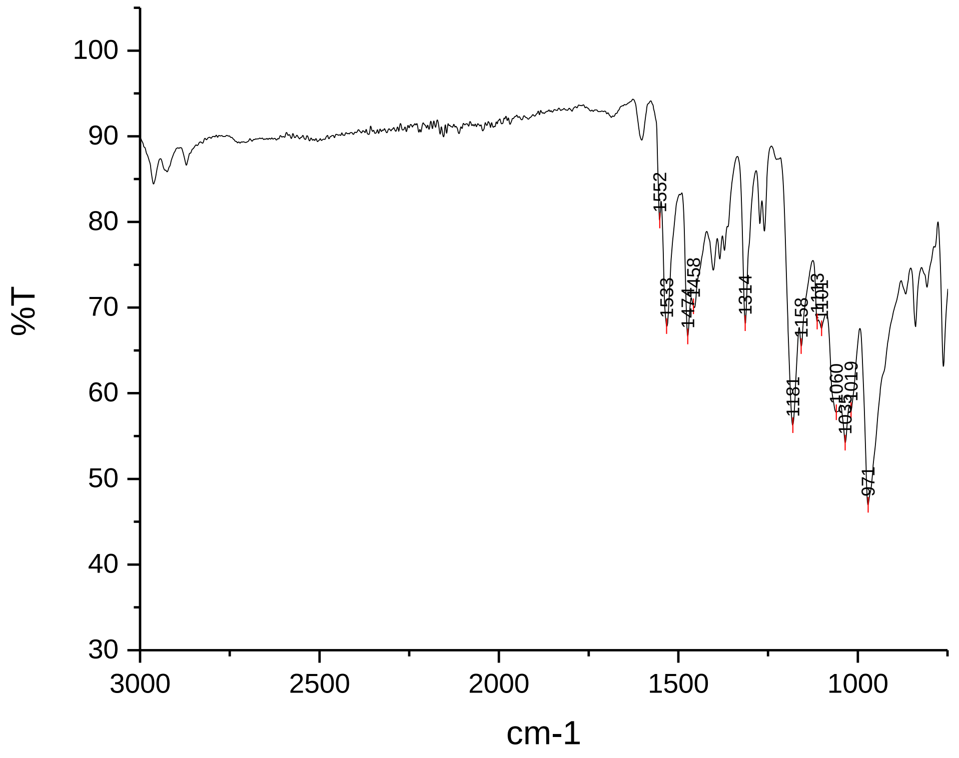

Figure S2-1. Solid state infrared spectrum of compound **ethynyl-BODIPY$^{\mathbf{Me,Et,Me}}$** (**1**).

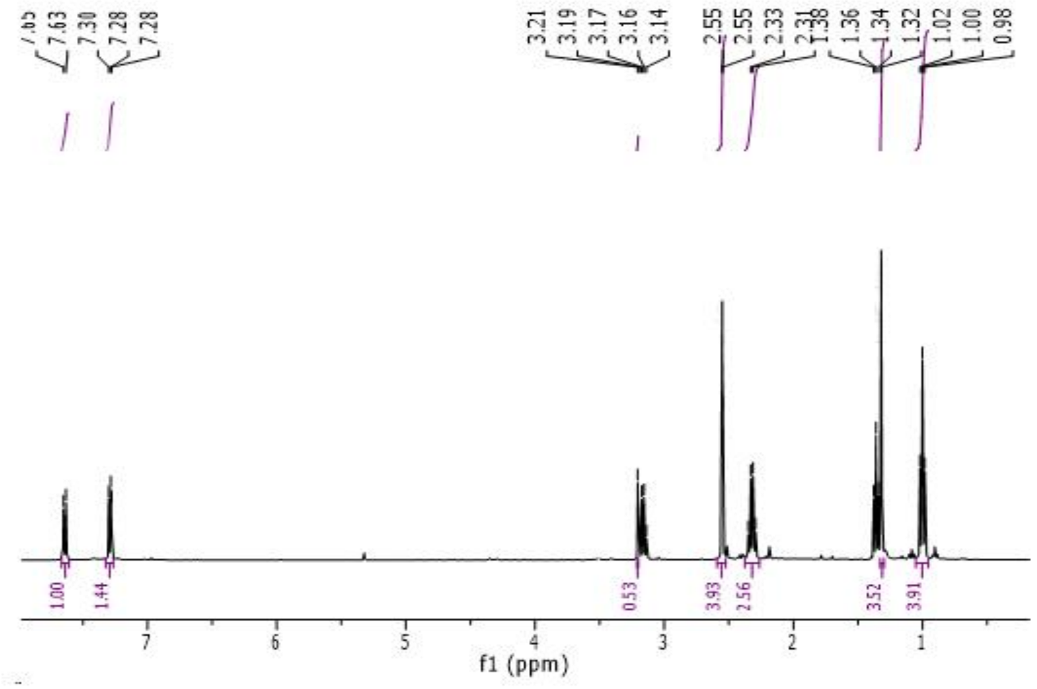

Figure S2-2. ^{1}H NMR spectrum of compound **ethynyl-BODIPY$^{\mathbf{Me,Et,Me}}$** (**1**) in CD_2Cl_2.

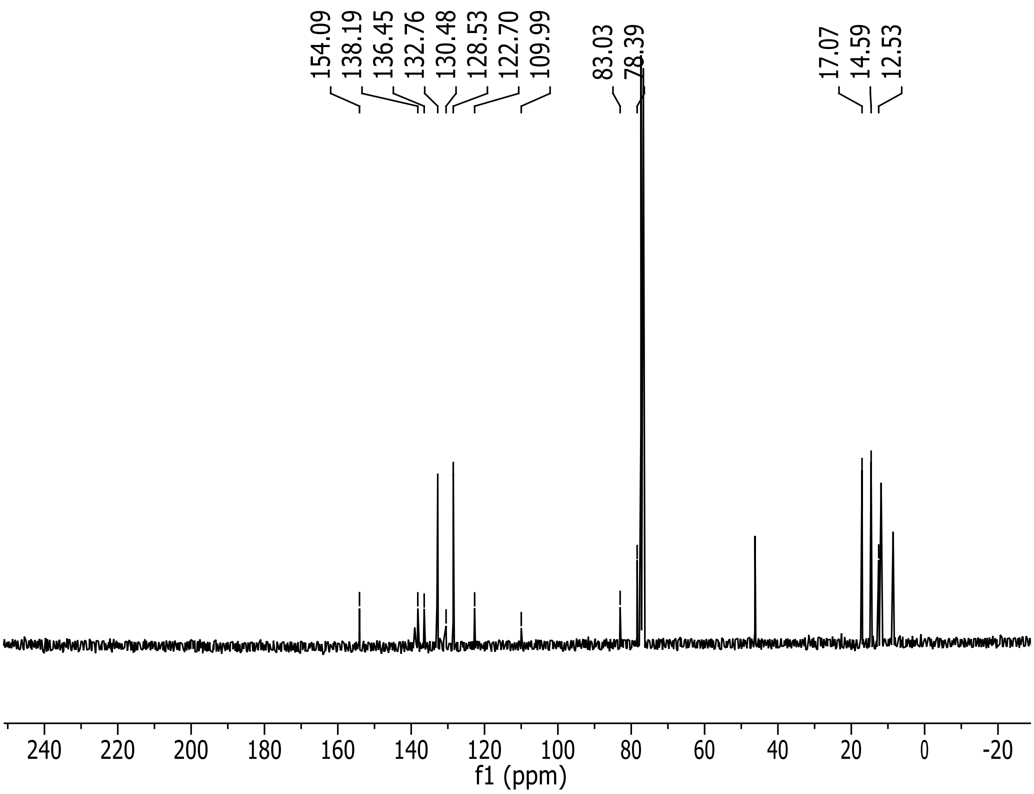

Figure S2-3. $^{13}C\{^1H\}$ NMR spectrum of compound **ethynyl-BODIPY^{Me,Et,Me}** (**1**) in $CDCl_3$.

Synthesis of ethynyl-BODIPY^{Me,Me} (2)

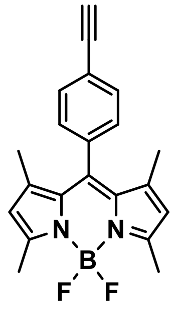

2,4-Dimethylpyrrole (970 µL, 9.40 mmol) was treated with 4-ethynylbenzaldehyde (600 mg, 4.60 mmol) according to the general procedure[S4] giving an orange powder (472 mg, 27%). IR (solid state): 1543, 1511, 1467, 1438, 1409, 1369, 1309, 1194, 1185, 1155, 1123, 1108, 1087, 1064, 1038, 1018, 1001, 971, 900 cm^{-1}. ^1H NMR (400 MHz, CD_2Cl_2): δ 7.66, 7.29 (AB, 2 x 2H, J_{HH} = 7.8 Hz, C_6H_4), 6.01 (s, 2H, pyrrole-CH), 3.20 (s, 1H, C≡CH), 2.58 (s, 6H, pyrrole-CH_3), 1.42 (s, 6H, pyrrole-CH_3) ppm. $^{11}B\{^1H\}$ NMR (128 MHz, $CDCl_3$): δ 0.75 (t, J_{BF} = 33.1 Hz) ppm. $^{13}C\{^1H\}$ NMR (101 MHz, $CDCl_3$): δ 155.8, 142.9, 140.5, 135.5, 132.8, 131.1, 128.2, 121.4, 82.8, 78.5, 14.5 ppm. $^{19}F\{^1H\}$ NMR (377 MHz, $CDCl_3$): δ -146.27 (q, J_{FB} = 32.8 Hz) ppm. HRMS (ES^+-TOF) *m/z* 349.1680 $[M+H]^+$, calculated for $C_{21}H_{20}{}^{11}BF_2N_2$: 349.1688.[S6]

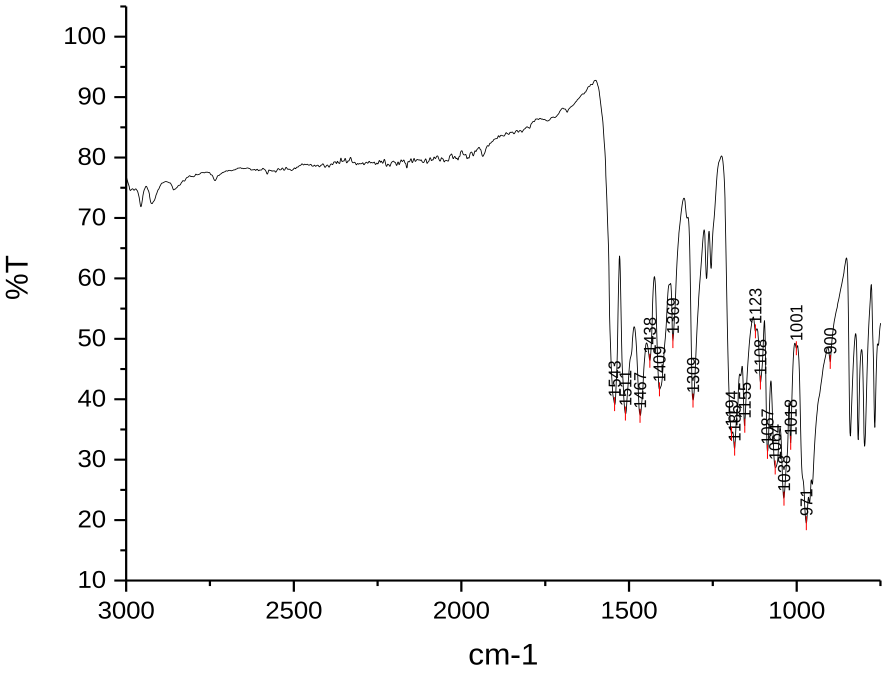

Figure S2-4. Solid-state infrared spectrum of **ethynyl-BODIPY^{Me,Me}** (**2**).

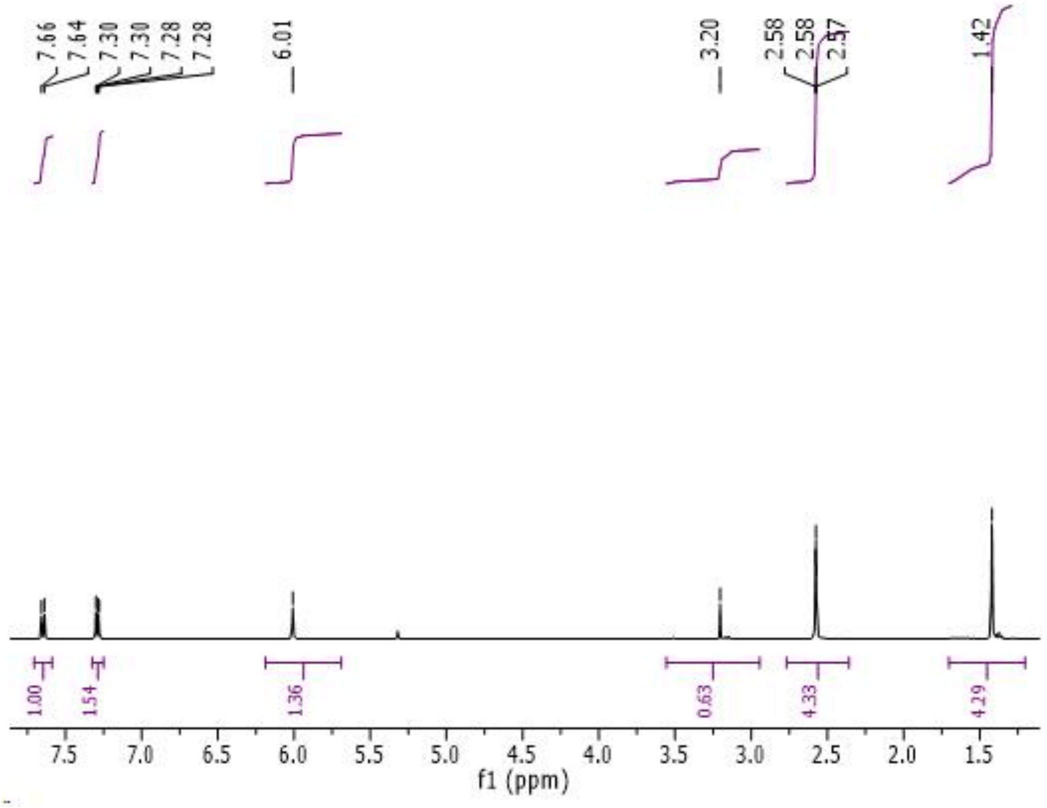

Figure S2-5. ^{1}H NMR spectrum of **ethynyl-BODIPY^{Me,Me}** (**2**) in CD_2Cl_2.

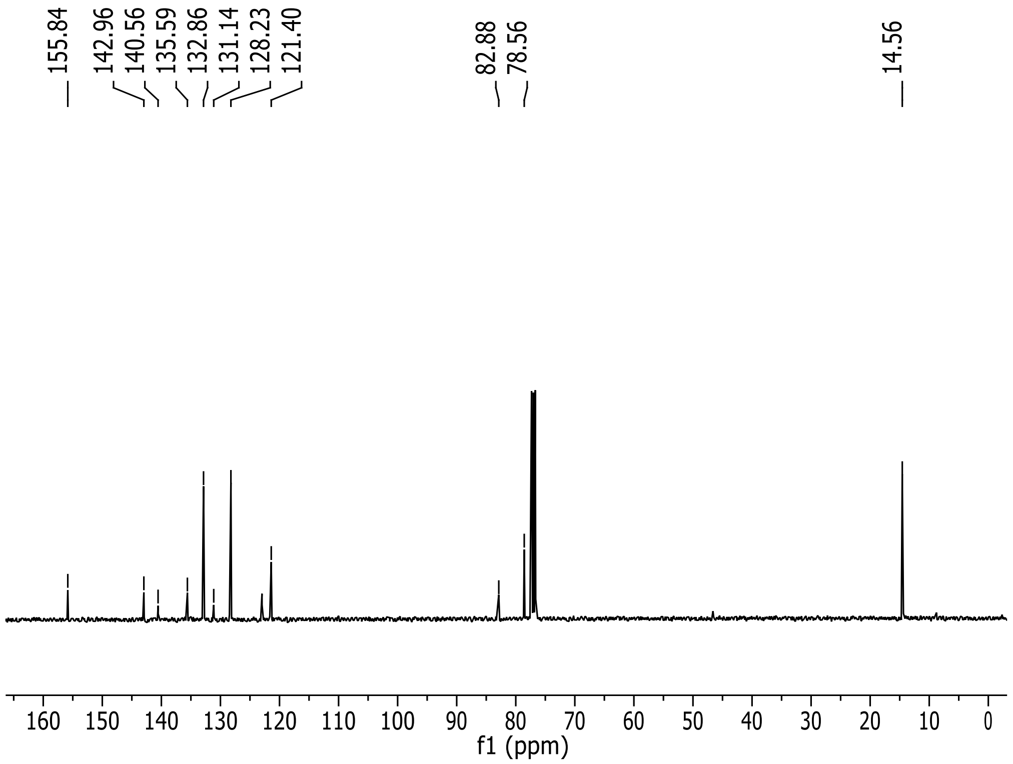

Figure S2-6. $^{13}C\{^{1}H\}$ NMR spectrum of **ethynyl-BODIPY^{Me,Me}** (**2**) in $CDCl_3$.

Synthesis of ethynyl-BODIPY^{Et} (3)

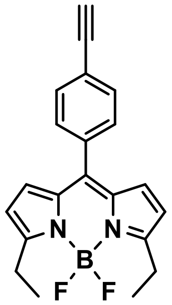

2-Ethylpyrrole (960 µL, 9.40 mmol) and 4-ethynylbenzaldehyde (600 g, 4.60 mmol) were reacted according to the general procedure[S4] giving a red powder (600 mg, 35%). IR (solid state): 1562, 1542, 1489, 1429, 1320, 1275, 1138, 1028, 1007, 999, 980, 960, 884, 843 cm^{-1}. ^{1}H NMR (400 MHz, $CDCl_3$): δ 7.65, 7.53 (AB, 2 x 2H, J_{HH} = 8.2 Hz, C_6H_4), 6.80 (d, 2H, J_{HH} = 4.3 Hz, pyrrole-CH), 6.44 (d, 2H, J_{HH} = 4.2 Hz, pyrrole-CH), 3.32 (s, 1H, C≡CH), 3.07 (q, 4H, J_{HH} = 7.6 Hz, pyrrole-CH_2), 1.36 (t, 6H, J_{HH} = 7.7 Hz, pyrrole-CH_3) ppm. $^{11}B\{^{1}H\}$ NMR (128 MHz, $CDCl_3$): δ 0.96 (t, J_{BF} = 32.7 Hz). $^{13}C\{^{1}H\}$ NMR (101 MHz, $CDCl_3$): δ 164.0, 141.6, 134.6, 134.0, 131.9, 130.3, 130.2, 123.9, 117.5, 117.5, 82.8, 79.2, 22.1, 12.8 ppm. $^{19}F\{^{1}H\}$ NMR ($CDCl_3$): δ -145.25 (q, J_{FB} = 33.0 Hz) ppm. HRMS (ES^{+}-TOF) *m/z* 349.1701 [M+H]$^{+}$, calculated for $C_{21}H_{20}{}^{11}BF_2N_2$: 349.1688.[S7]

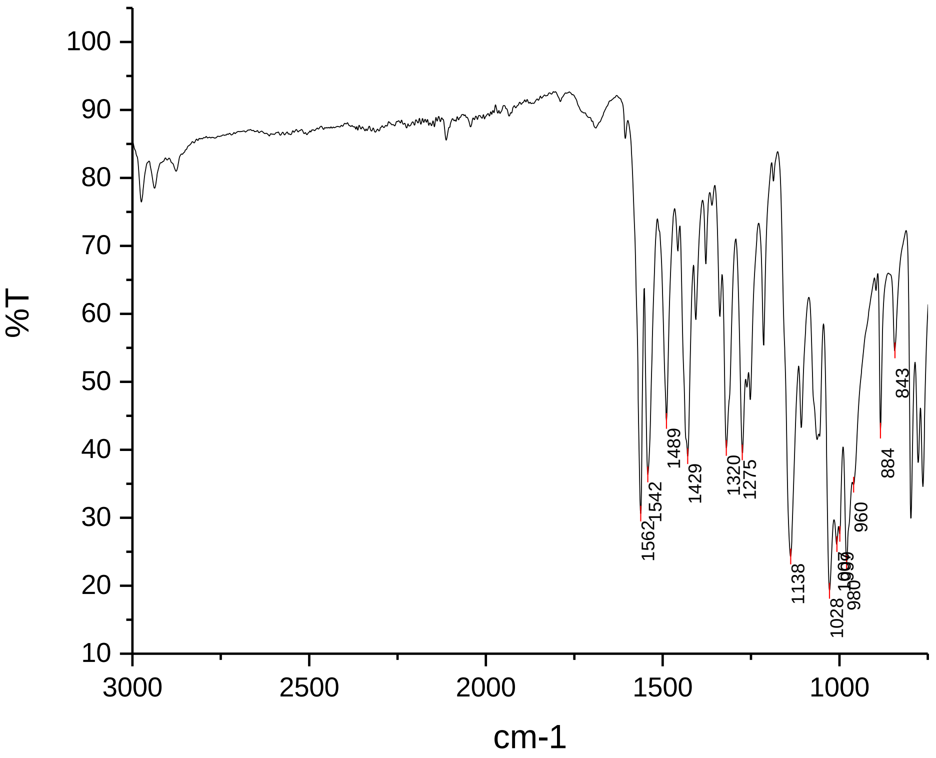

Figure S2-7. Solid-state infrared spectrum of **ethynyl-BODIPY^{Et}** (**3**).

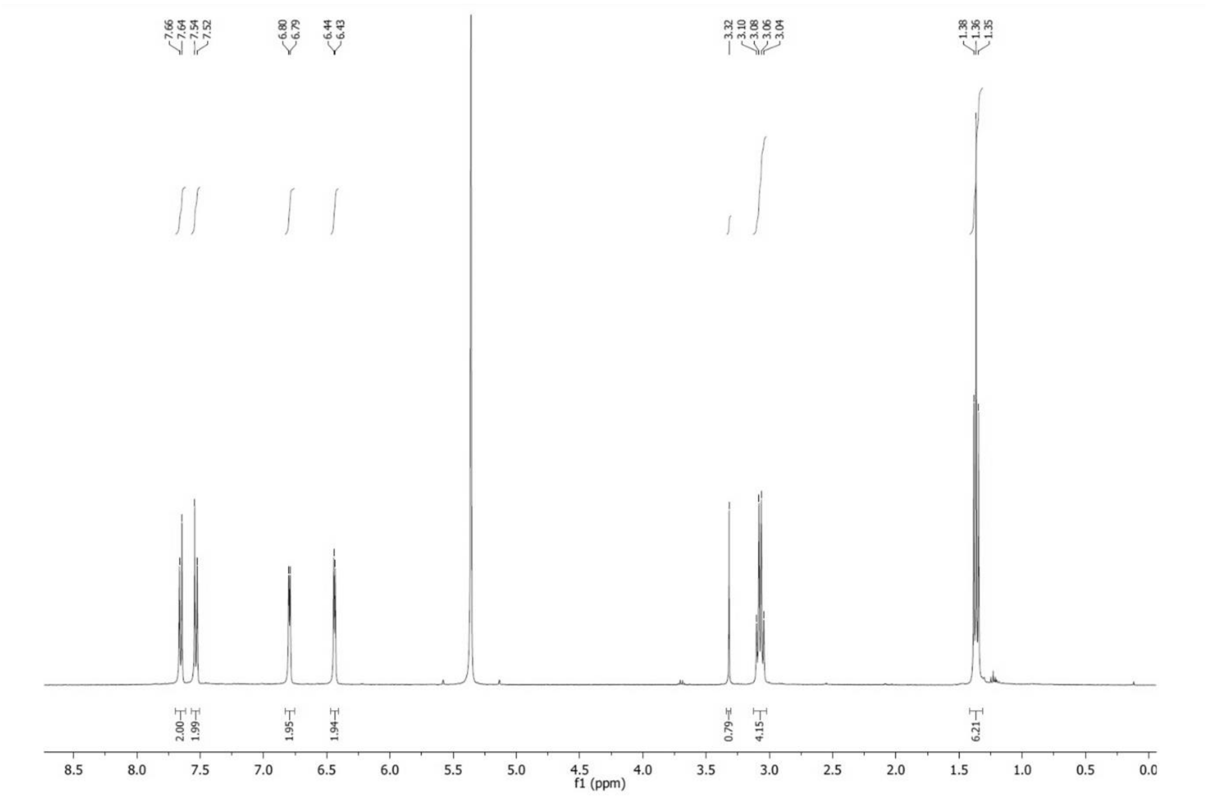

Figure S2-8. ^{1}H NMR spectrum of **ethynyl-BODIPY^{Et}** (**3**) in $CDCl_3$.

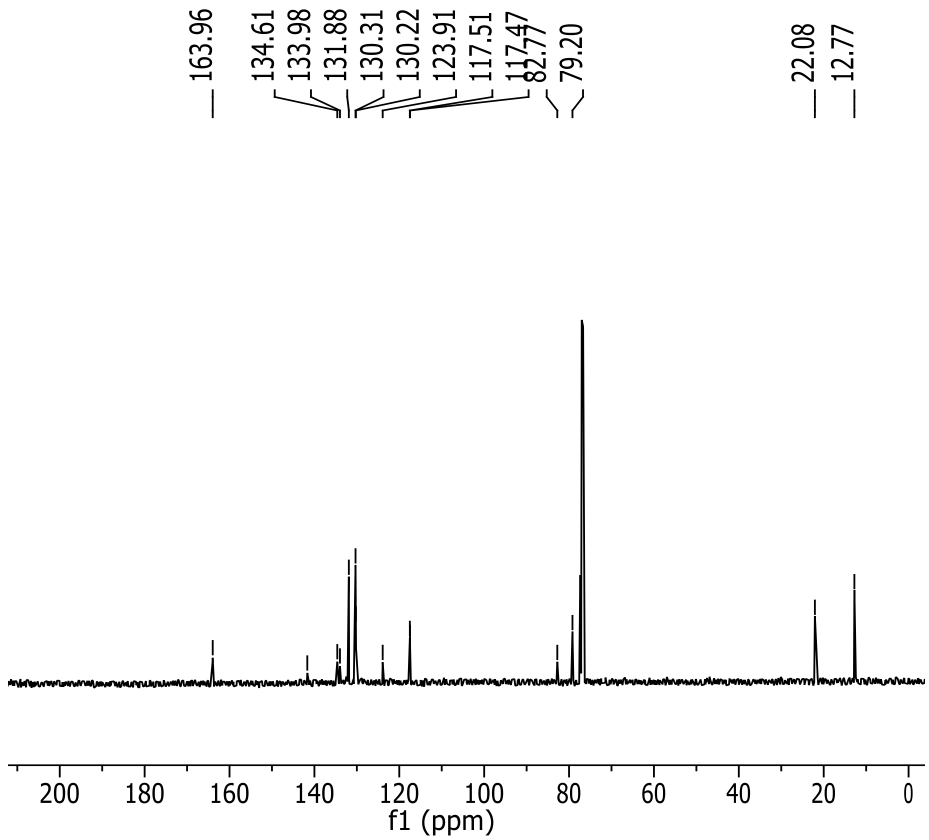

Figure S2-9. $^{13}C\{^{1}H\}$ NMR spectrum of **ethynyl-BODIPY^{Et}** (**3**) in $CDCl_3$.

S2.2 Synthetic procedure for preparation of ruthenium BTD complexes

The ruthenium hydride precursor, $[RuH(Cl)(CO)(PPh_3)_3]$ (1 eq.), was first added to dichloromethane (10 mL) to form an off-white suspension. The ethynyl-BODIPY ligand (1.1 eq.) and appropriate amine (1.1 eq.) were then added and the reaction mixture was left to stir at room temperature for 30 min. All solvent was subsequently removed and petroleum ether (40-60 °C) (10 mL) was added to the crude product. The suspension was triturated for 10 min and then filtered. All products were isolated as orange/red powders.

Synthesis of $[Ru(CH{=}CH\text{-}BODIPY^{Me,Et,Me})Cl(CO)(BTD)(PPh_3)_2]$ (4)

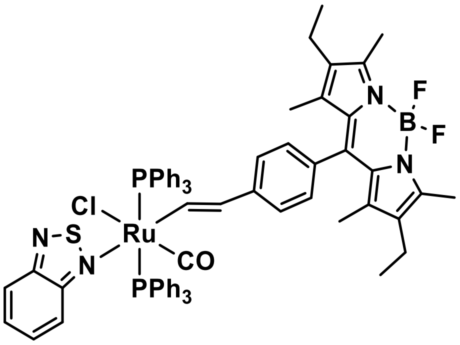

$[RuH(Cl)(CO)(CO)(PPh_3)_3]$ (110 mg, 0.12 mmol) was treated with **ethynyl-BODIPY^{Me,Et,Me}** (**1**) (52 mg, 0.13 mmol) and 2,1,3-benzothiadiazole (17 mg, 0.13 mmol) according to the general procedure, producing a red powder (138 mg, 92%). IR (solid state): 1928 (CO), 1571, 1538, 1481, 1435, 1321, 1192, 1161, 1092, 1071, 999, 979, 850 cm^{-1}. ^{1}H NMR (400 MHz, $CDCl_3$): δ 8.75 (m, 1H, H_α), 7.95 (s, 2H, BTD), 7.82 – 6.65 (m, 30H + 2H + 4H, C_6H_5 + BTD + C_6H_4), 5.85 (d, 1H, J_{HH} = 15.8 Hz, H_β), 2.56 (s, 6H, pyrrole-CH_3), 2.33 (m, 4H, pyrrole-CH_2), 1.35 (s, 6H, pyrrole-CH_3), 0.90 (m, 6H, pyrrole-CCH_3) ppm. $^{11}B\{^1H\}$ NMR (128 MHz, $CDCl_3$): δ 0.85 (t, J_{BF} = 32.8 Hz) ppm. $^{19}F\{^1H\}$ NMR (377 MHz, $CDCl_3$): δ -145.80 (q, J_{FB} = 30.4 Hz) ppm. $^{31}P\{^1H\}$ NMR (162 MHz, $CDCl_3$): δ 27.4 (s) ppm. MS (ES^+-TOF) *m/z* (abundance): 1230 (2) $[M]^+$, 1202 (22) $[M - CO]^+$, 1141 (100) $[M - Cl - BTD + 2MeCN]^+$, 1100 (100) $[M - Cl - BTD + MeCN]^+$. Elemental analysis: Calculated for $C_{68}H_{62}BClF_2N_4OP_2RuS$ (Mw = 1230.59) C, 66.4; H, 5.1; N, 4.6%. Found C, 66.6; H, 4.8; N, 5.0%.

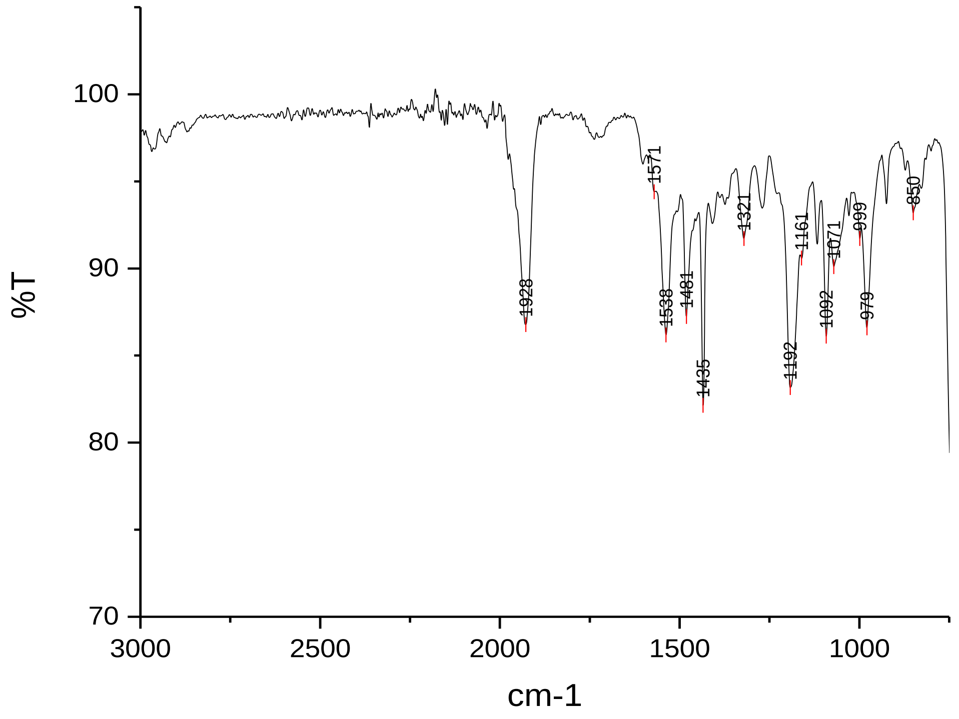

Figure S2-10. Infrared spectrum of **[Ru(CH=CH-BODIPY^{Me,Et,Me})Cl(CO)(BTD)(PPh$_3$)$_2$]** (**4**) in the solid state.

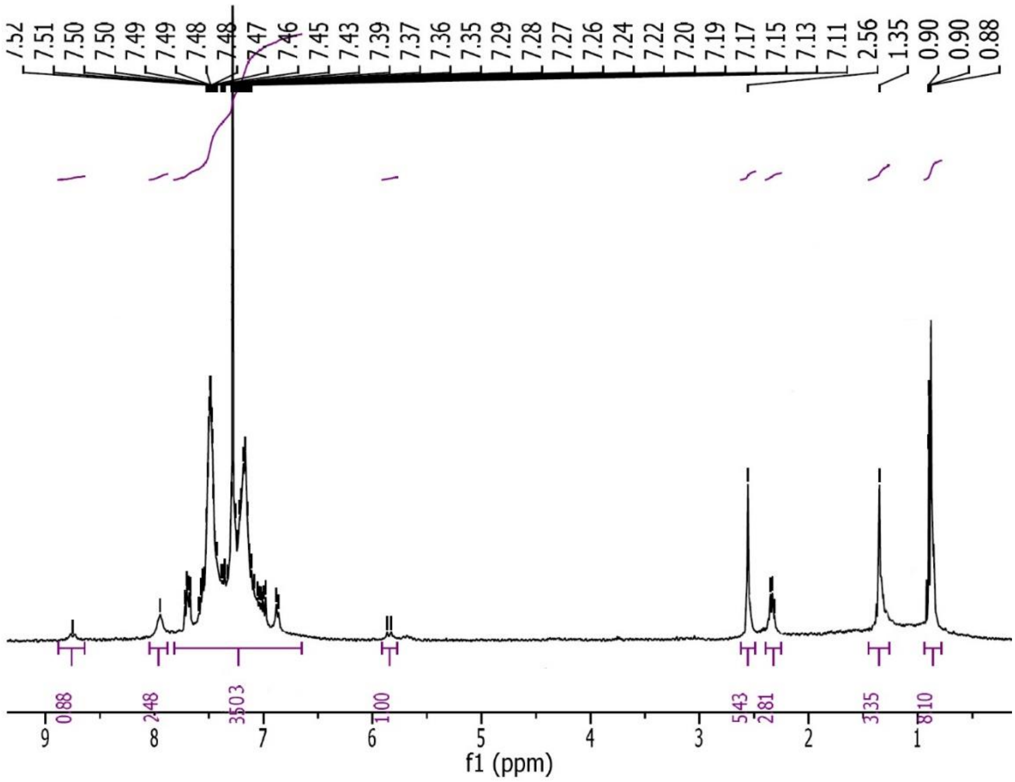

Figure S2-11. ^{1}H NMR spectrum of **[Ru(CH=CH-BODIPY^{Me,Et,Me})Cl(CO)(BTD)(PPh$_3$)$_2$]** (**4**) in $CDCl_3$.

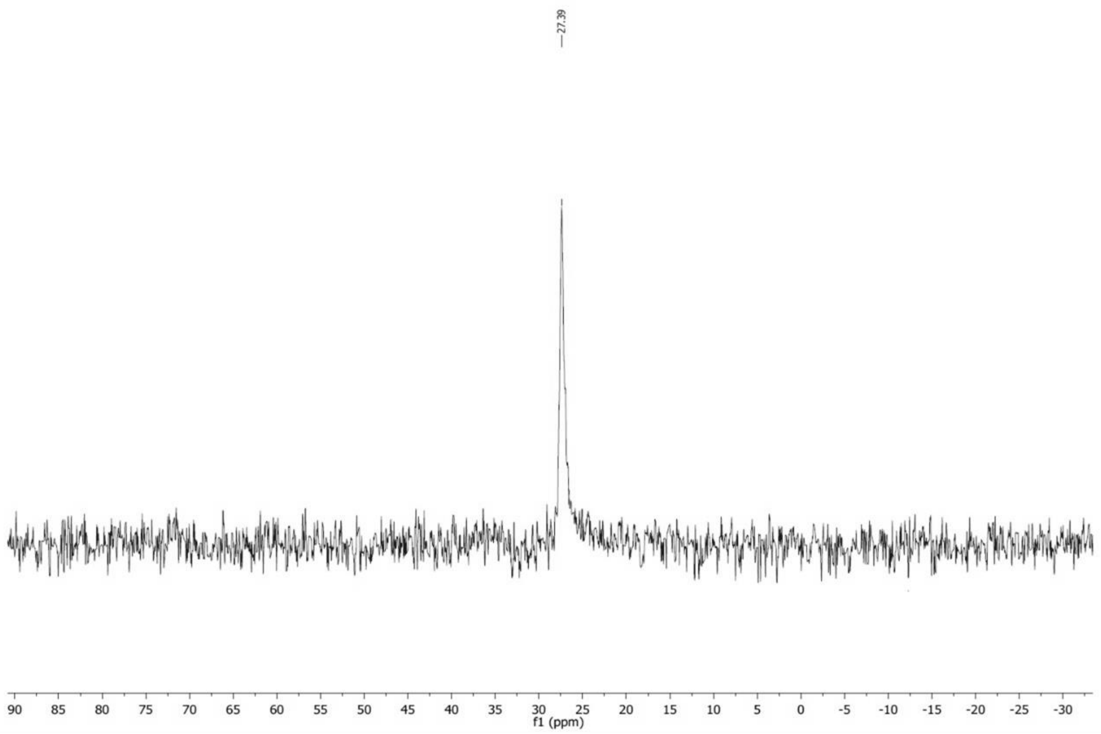

Figure S2-12. $^{31}P\{^{1}H\}$ NMR spectrum of **[Ru(CH=CH-BODIPY^{Me,Et,Me})Cl(CO)(BTD)(PPh$_3$)$_2$]** (**4**) in $CDCl_3$.

Synthesis of [Ru(CH=CH-BODIPY^{Me,Me})Cl(CO)(BTD)(PPh$_3$)$_2$] (5)

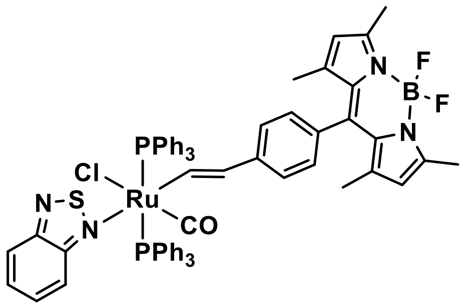

[RuH(Cl)(CO)(PPh$_3$)$_3$] (110 mg, 0.12 mmol) was treated with **ethynyl-BODIPY^{Me,Me}** (**2**) (44 mg, 0.13 mmol) and 2,1,3-benzothiadiazole (17 mg, 0.13 mmol) according to the general procedure, producing an orange powder (92 mg, 83%). IR (solid state): 1924 (CO), 1568, 1542, 1508, 1482, 1471, 1434, 1410, 1372, 1307, 1194, 1155, 1088, 1051, 980, 845 cm^{-1}. ^{1}H NMR (400 MHz, $CDCl_3$): δ 8.75 (d, 1H, J_{HH} = 15.9 Hz, H_α), 7.97 (s, 2H, BTD), 7.54 (m, 2H, BTD), 7.48, 7.28, 7.17 (m x 3, 30H, C_6H_5), 6.99, 6.87 (AB, 2 x 2H, J_{HH} = 7.9 Hz, C_6H_4), 6.00 (s, 2H, pyrrole-CH), 5.84 (d, 1H, J_{HH} = 15.9 Hz, H_β), 2.58 (s, 6H, pyrrole-CH$_3$), 1.45 (s, 6H, pyrrole-CH$_3$) ppm. $^{11}B\{^{1}H\}$ NMR (128 MHz, $CDCl_3$): δ 0.85 ppm (t, J_{BF} = 30.4 Hz). $^{19}F\{^{1}H\}$ NMR (377 MHz, $CDCl_3$): δ -145.80 (q, J_{FB} = 30.4 Hz) ppm. $^{31}P\{^{1}H\}$ NMR (162 MHz, $CDCl_3$): δ 28.2 (s) ppm. MS (ES$^+$-TOF) m/z (abundance): 1174 (2) [M]$^+$, 1085 (23) [M – CO + K]$^+$, 1042 (100) [M – Cl – BTD + K]$^+$. Elemental analysis: Calculated for $C_{64}H_{54}BClF_2N_4OP_2RuS$ (Mw = 1174.48) C, 65.5; H, 4.6; N, 4.8%. Found C, 65.3; H, 4.7; N, 4.7%.

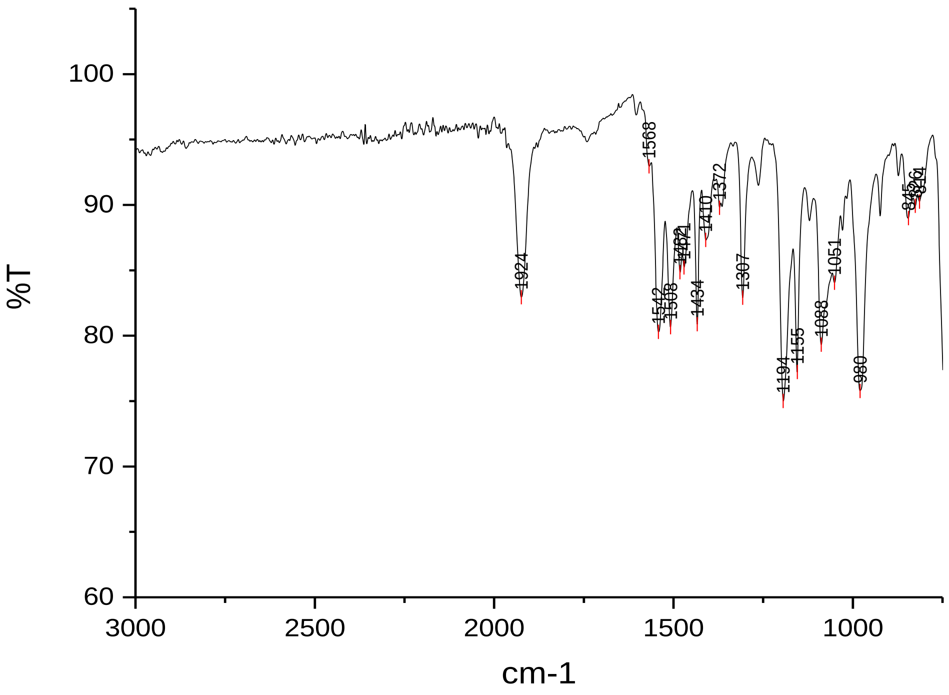

Figure S2-13. Infrared spectrum of **[Ru(CH=CH-BODIPY^{Me,Me})Cl(CO)(BTD)(PPh$_3$)$_2$]** (**5**) in the solid state.

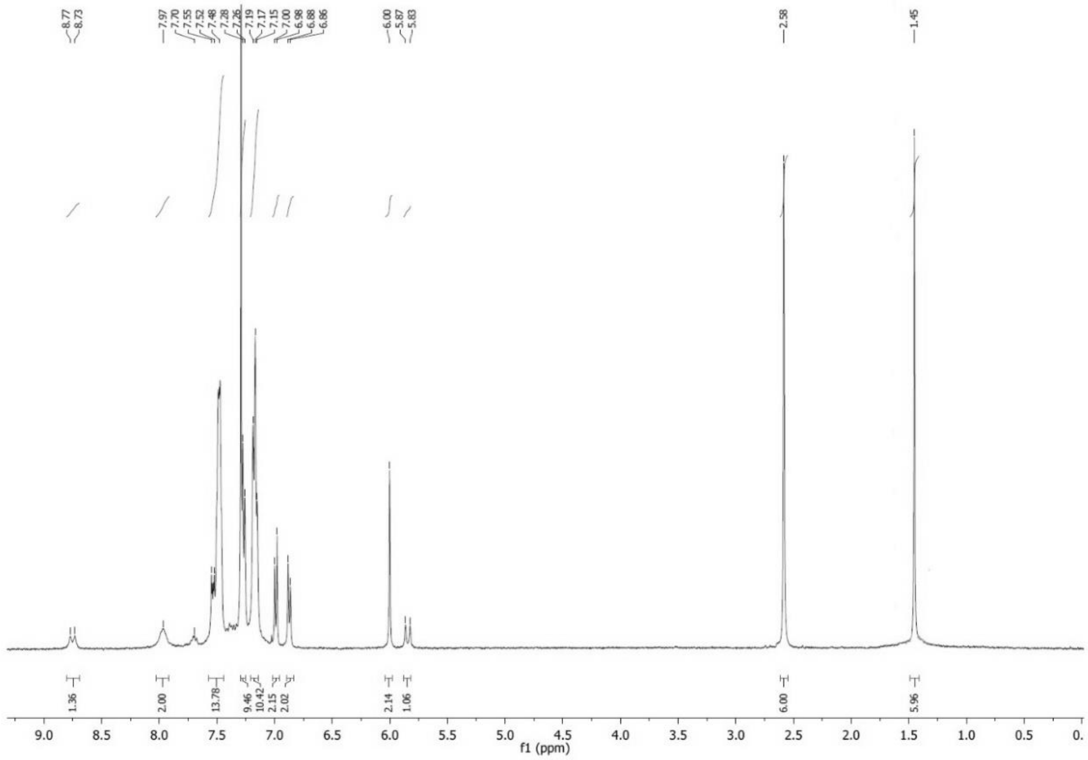

Figure S2-14. ^{1}H NMR spectrum of **[Ru(CH=CH-BODIPY^{Me,Me})Cl(CO)(BTD)(PPh$_3$)$_2$]** (**5**) in CDCl$_3$.

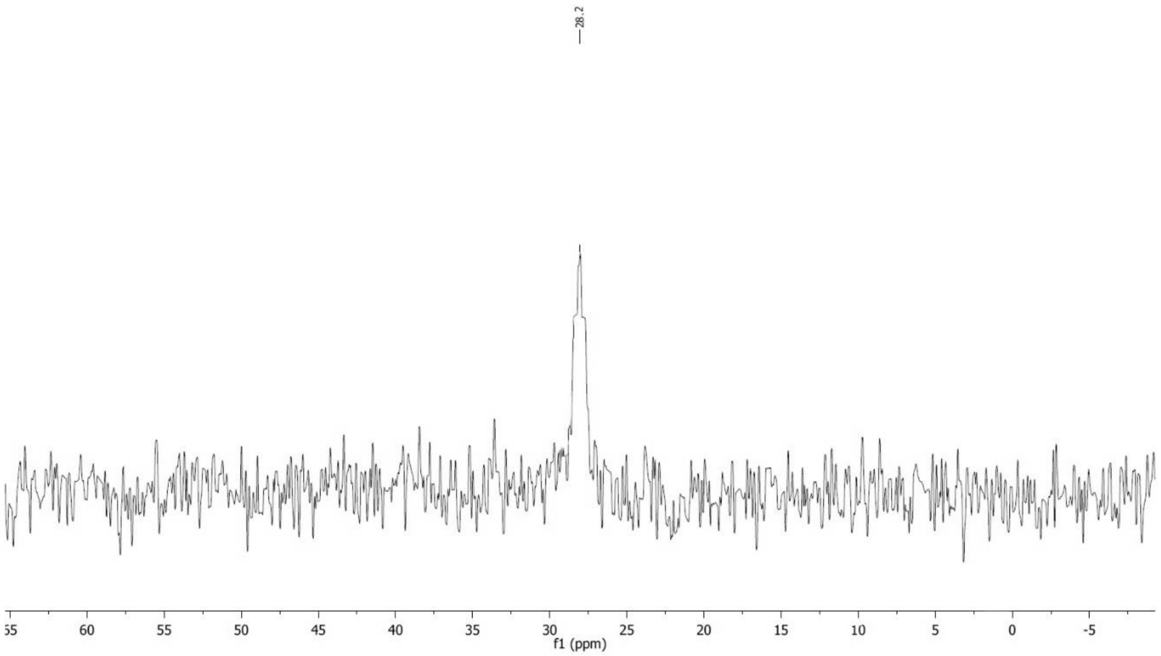

Figure S2-15. $^{31}P\{^{1}H\}$ NMR spectrum of **[Ru(CH=CH-BODIPY^{Me,Me})Cl(CO)(BTD)(PPh$_3$)$_2$]** (**5**) in $CDCl_3$.

Synthesis of [Ru(CH=CH-BODIPY^{Et})Cl(CO)(BTD)(PPh$_3$)$_2$] (6)

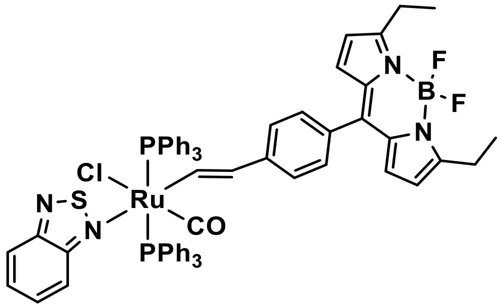

[RuH(Cl)(CO)(PPh$_3$)$_3$] (43 mg, 0.05 mmol) was treated with **3** (20 mg, 0.06 mmol) and 2,1,3-benzothiadiazole (8 mg, 0.06 mmol) according to the general procedure, producing a red powder (44 mg, 72%). IR (solid state): 1919 (CO), 1562, 1545, 1516, 1485, 1433, 1399, 1316, 1260, 1135, 1084, 1064, 1046, 1012, 999, 978 cm^{-1}. ^{1}H NMR (400 MHz, CD_2Cl_2): δ 9.15 (dt, 1H, J_{HH} = 16.3, J_{HP} = 3.2 Hz, H_α), 7.95 (m, 2H, BTD), 7.54 (m, 2H, BTD), 7.49 - 7.25 (m, 30H + 2H, C_6H_5 + C_6H_4), 7.00 (d, 2H, J_{HH} = 8.1 Hz, C_6H_4), 6.91 (d, 2H, J_{HH} = 4.3 Hz, pyrrole-CH), 6.44 (d, 2H, J_{HH} = 4.3 Hz, pyrrole-CH), 5.94 (d, 1H, J_{HH} = 16.3 Hz, H_β), 3.07 (q, 4H, J_{HH} = 7.6 Hz, pyrrole-CH_2), 1.37 (t, 6H, J_{HH} = 7.6 Hz, pyrrole-CCH_3) ppm. $^{11}B\{^{1}H\}$ NMR (128 MHz, CD_2Cl_2): δ 0.95 (t, J_{BF} = 32.9 Hz) ppm. $^{19}F\{^{1}H\}$ NMR (377 MHz, CD_2Cl_2): δ -145.24 (q, J_{FB} = 32.5 Hz) ppm. $^{13}C\{^{1}H\}$ NMR (101 MHz, CD_2Cl_2): δ 162.4 (s, BODIPY-C8), 159.4 (t, J_{CP} = 14.2 Hz, CO), 154.4 (s, BTD), 144.0 (s, C_6H_4), 142.1 (s, BODIPY), 137.4 (s(br), Cα), 134.2 (t^v, J_{CP} = 5.1 Hz, *o/m*-C_6H_5), 134.1 (s, BODIPY), 134.0 (s, C_6H_4), 131.9 (t^v, J_{CP} = 21.9 Hz, *ipso*-C_6H_5),

131.0 (s, *o/m*-C_6H_4), 130.4 (s, BODIPY), 129.7 (*p*-C_6H_5), 129.3 (t^v, J_{CP} = 4.9 Hz, *o/m*-C_6H_5), 127.6 (s, BTD), 123.8 (s, *o/m*-C_6H_4), 121.2 (s(br), Cβ), 116.8 (s, BODIPY), 21.9 (s, BODIPY-CH_2), 12.6 (s, BODIPY-CH_3) ppm. $^{31}P\{^1H\}$ NMR (162 MHz, CD_2Cl_2): δ 26.5 (s) ppm. MS (ES^+-TOF) *m/z* (abundance): 1174 (1) $[M]^+$, 1085 (100) $[M – CO + K]^+$, 1044 (100) $[M – Cl – BTD + MeCN]^+$. Calculated for $C_{64}H_{54}BClF_2N_4OP_2RuS$ (Mw = 1174.48) C, 65.5; H, 4.6; N, 4.8%. Found: C, 65.7; H, 4.6; N, 4.9%.

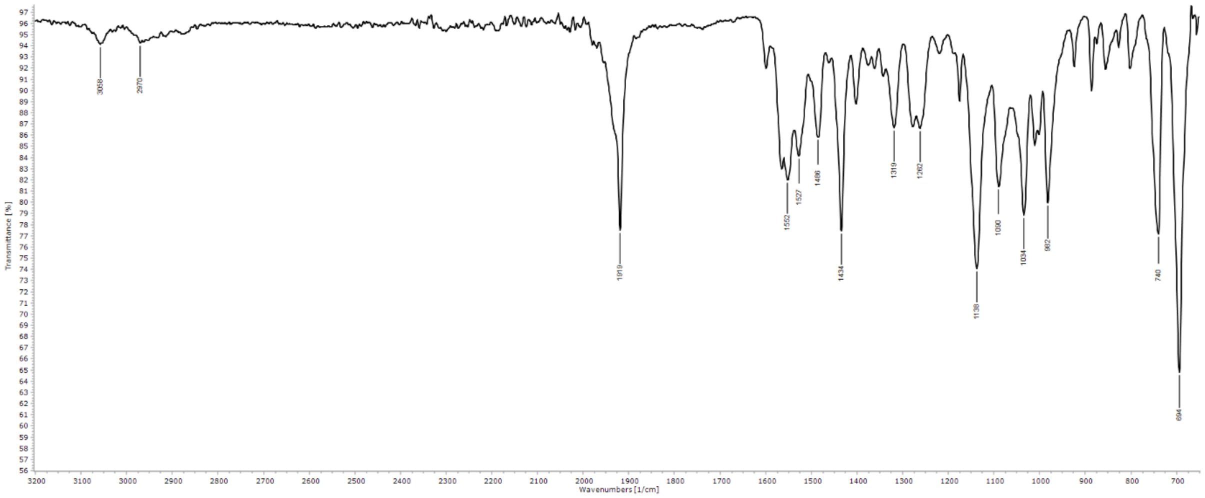

Figure S2-16. Infrared spectrum of **[Ru(CH=CH-BODIPY^{Et})Cl(CO)(BTD)(PPh$_3$)$_2$]** (**6**) in the solid state.

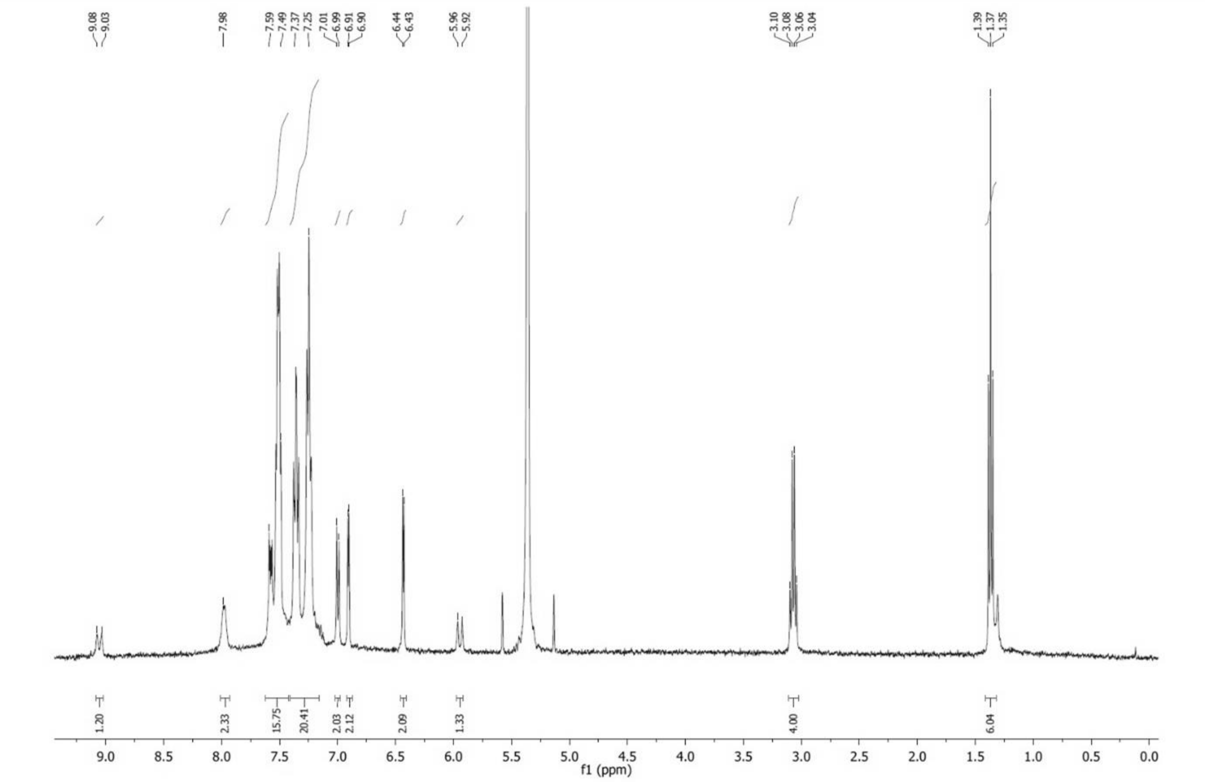

Figure S2-17. ^{1}H NMR spectrum of **[Ru(CH=CH-BODIPY^{Et})Cl(CO)(BTD)(PPh$_3$)$_2$]** (**6**) in CD_2Cl_2.

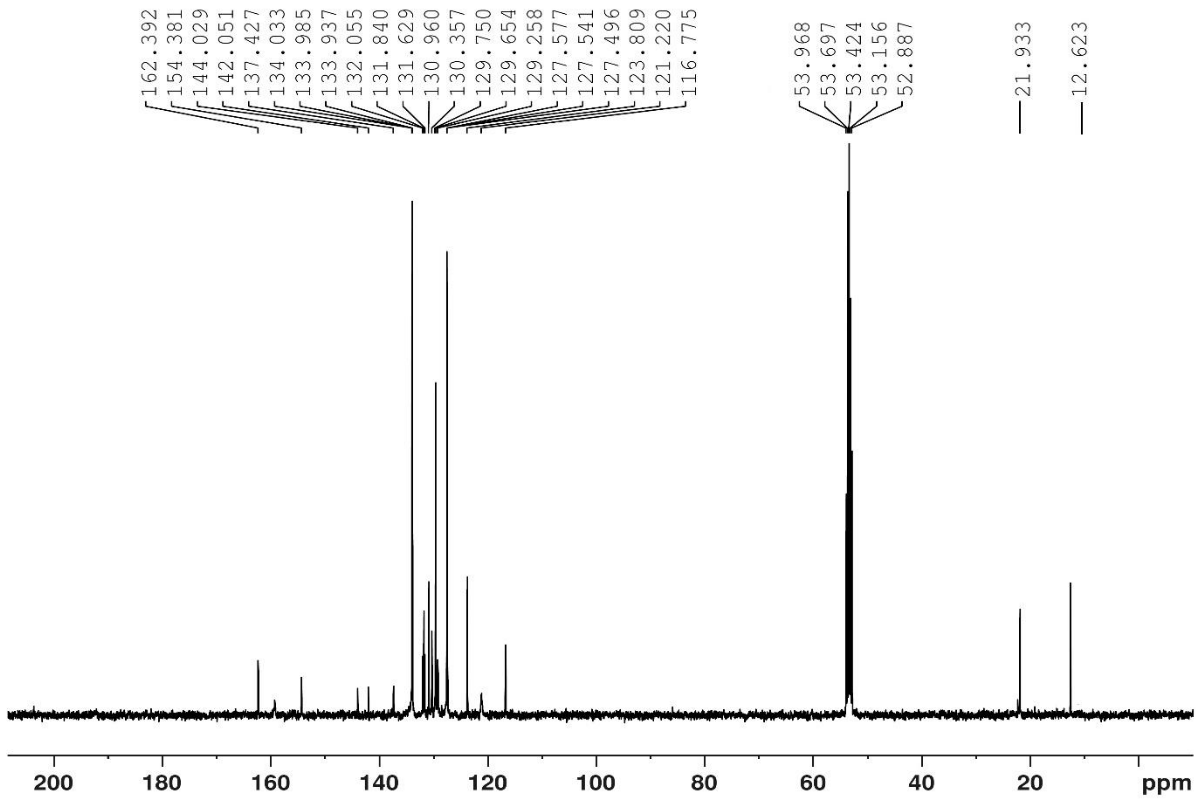

Figure S2-18. $^{13}C\{^1H\}$ NMR spectrum of **[Ru(CH=CH-BODIPY^{Et})Cl(CO)(BTD)(PPh$_3$)$_2$]** (**6**) in CD_2Cl_2.

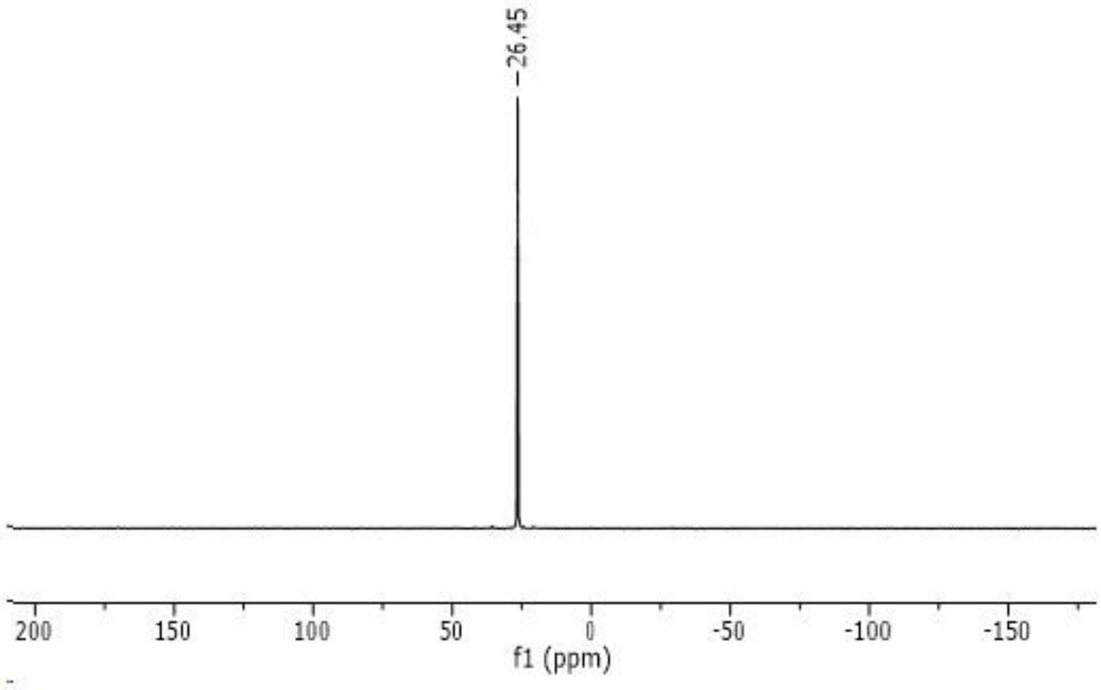

Figure S2-19. $^{31}P\{^1H\}$ NMR spectrum of **[Ru(CH=CH-BODIPY^{Et})Cl(CO)(BTD)(PPh$_3$)$_2$]** (**6**) in CD_2Cl_2.

S2.3 Synthetic procedure for preparation of ruthenium carbonyl adducts

Synthesis of [Ru(CH=CH-BODIPY^{Me,Et,Me})Cl(CO)$_2$(PPh$_3$)$_2$] (4·CO)

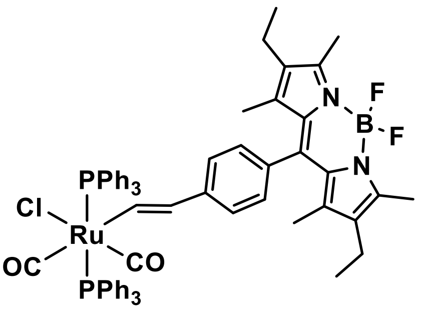

Carbon monoxide was bubbled through a dichloromethane solution (10 mL) of **4** (30 mg, 0.03 mmol) for 30 seconds causing no noticeable color change. All solvent was removed and petroleum ether (40 – 60 °C) (10 mL) was added to precipitate the crude product. The suspension was triturated ultrasonically for 10 min and then filtered. The product was isolated as a bright pink powder (24 mg, 77%). IR (solid state): 2028 (CO), 1968 (CO), 1536, 1476, 1185, 976 cm^{-1}. ^{1}H NMR (400 MHz, CD_2Cl_2): δ 7.71 (m, 12H, C_6H_5), 7.64 (d, 1H, J_{HH} = 18.0 Hz, H_α), 7.01 (m, 18H, C_6H_5), 7.07, 6.96 (AB, 2 x 2H, J_{AB} = 7.9 Hz, C_6H_4), 6.04 (d, 1H, J_{HH} = 18.0 Hz, H_β), 2.53 (s, 6H, pyrrole-CH_3), 2.39 (q, 4H, J_{HH} = 7.5 Hz, pyrrole-CH_2), 1.40 (s, 6H, pyrrole-CH_3), 1.06 (t, 6H, J_{HH} = 7.5 Hz, pyrrole-CCH_3) ppm. ^{11}B{^{1}H} NMR (128 MHz, CD_2Cl_2): δ 0.77 (t, J_{BF} = 33.8 Hz) ppm. ^{13}C{^{1}H} NMR (101 MHz, CD_2Cl_2): δ 161.0 (t, J_{CP} = 13.8 Hz, CO), 153.2 (s, BODIPY-C8), 141.5 (s, BODIPY), 141.0 (s, BODIPY), 138.9 (s, C_6H_4), 136.7 (s(br), Cα), 134.3 (t^{v}, J_{CP} = 5.1 Hz, *o/m*-C_6H_5), 133.6 (s, BODIPY), 132.7 (s, C_6H_4), 132.5 (t^{v}, J_{CP} = 23.5 Hz, *ipso*-C_6H_5), 131.7 (s, *o/m*-C_6H_4), 130.9 (s, BODIPY), 130.2 (s, *p*-C_6H_5), 128.5 (s(br), Cβ), 128.1 (s, BODIPY), 128.0 (t^{v}, J_{CP} = 4.8 Hz, *o/m*-C_6H_5), 127.7 (s, *o/m*-C_6H_4), 125.2 (s, *o/m*-C_6H_4), 17.0 (s, BODIPY-CH_2), 14.4 (s, BODIPY-CH_3), 12.2 (s, BODIPY-CCH_3), 11.6 (s, BODIPY-CH_3) ppm. ^{19}F{^{1}H} NMR (377 MHz, CD_2Cl_2): δ -145.65 (q, J_{FB} = 32.5 Hz) ppm. ^{31}P{^{1}H} NMR (162 MHz, CD_2Cl_2): δ 25.1 (s) ppm. MS (ES^{+}-TOF) *m/z* (abundance): 1128 (100) [M – Cl + MeCN]$^{+}$, 1122 (4) [M]$^{+}$, 1100 (49) [M – Cl – CO + MeCN]$^{+}$. Elemental analysis: Calculated for $C_{63}H_{58}BClF_2N_2O_2P_2Ru\cdot0.5CH_2Cl_2$ (Mw = 1164.89) C, 65.5; H, 5.1; N, 2.4%. Found: C, 65.8; H, 5.1; N, 2.5%.

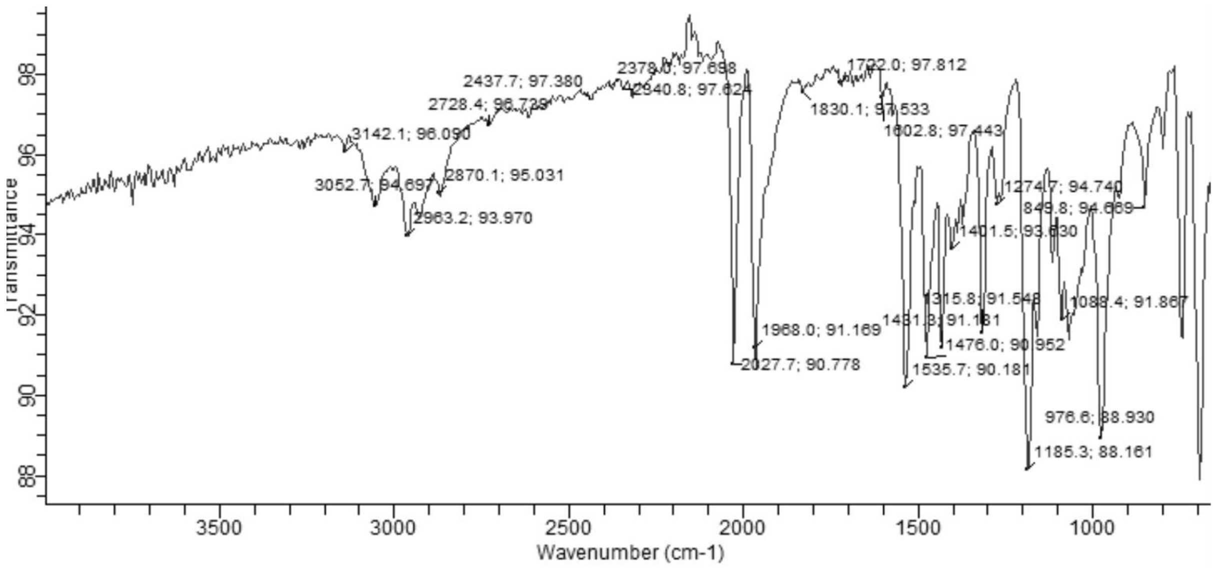

Figure S2-20. Infrared spectrum of **[Ru(CH=CH-BODIPY^{Me,Et,Me})Cl(CO)$_2$(PPh$_3$)$_2$]** (**4·CO**) in the solid state.

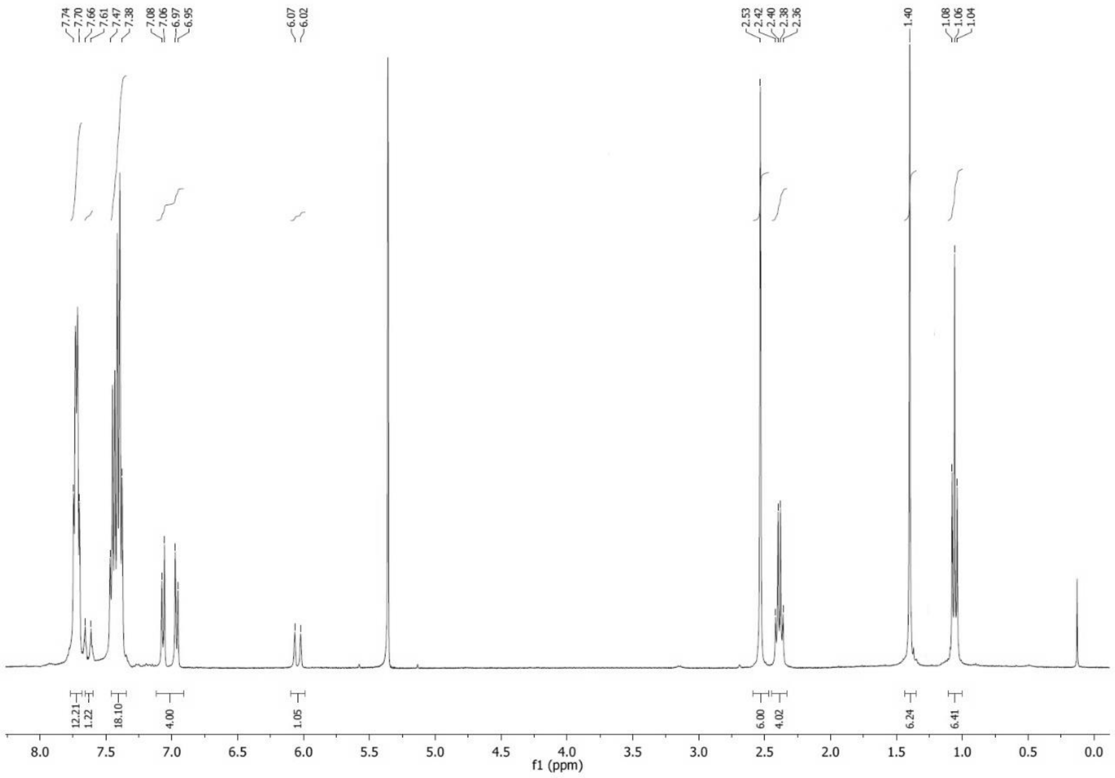

Figure S2-21. ^{1}H NMR spectrum of **[Ru(CH=CH-BODIPY^{Me,Et,Me})Cl(CO)$_2$(PPh$_3$)$_2$]** (**4·CO**) in CD_2Cl_2.

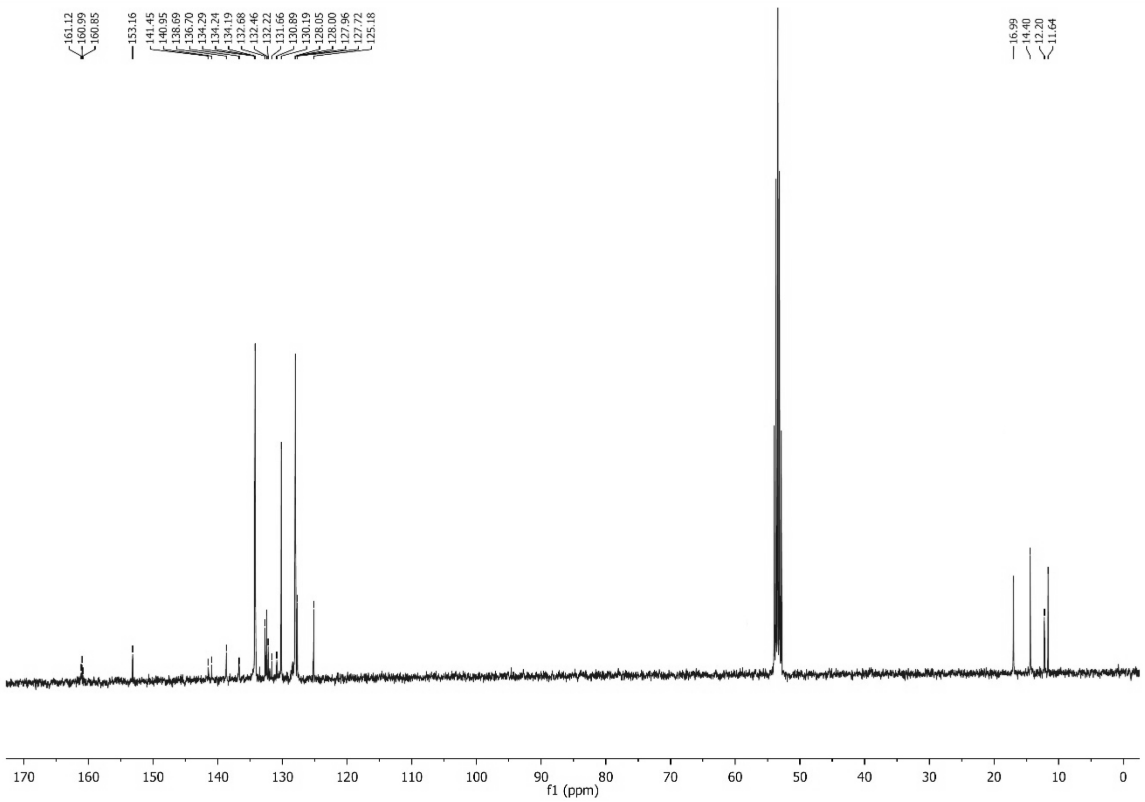

Figure S2-22. $^{13}C\{^1H\}$ NMR spectrum of **[Ru(CH=CH-BODIPY^{Me,Et,Me})Cl(CO)$_2$(PPh$_3$)$_2$]** (**4·CO**) in CD_2Cl_2.

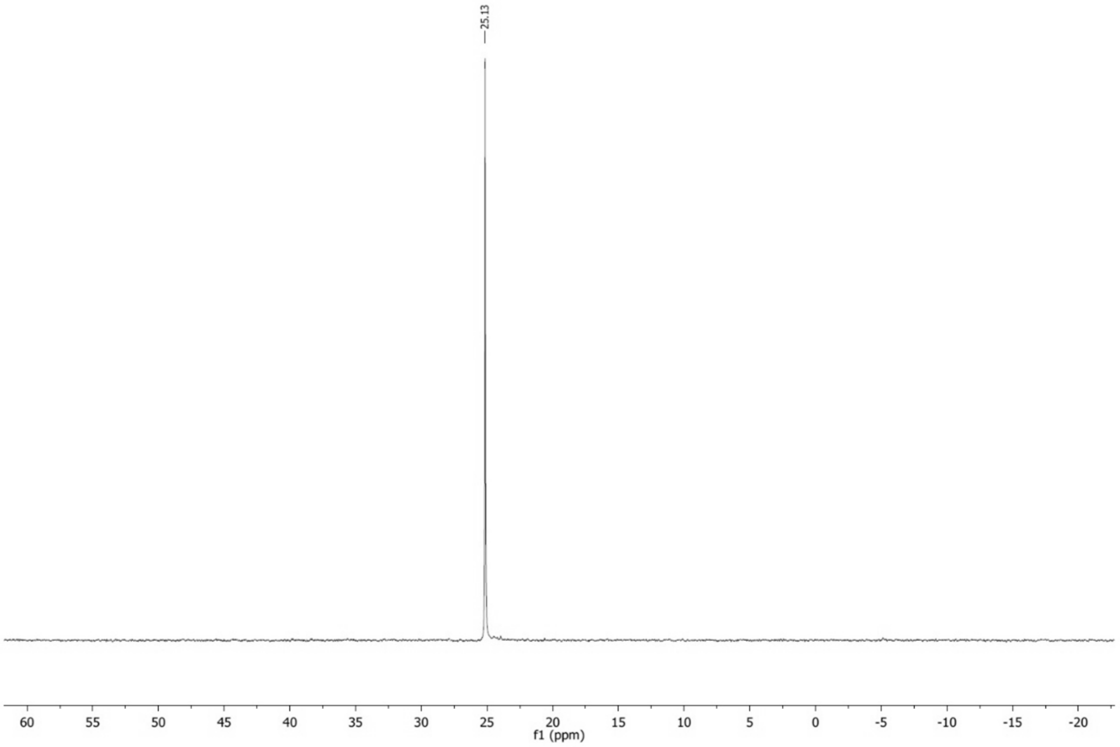

Figure S2-23. $^{31}P\{^1H\}$ NMR spectrum of **[Ru(CH=CH-BODIPY^{Me,Et,Me})Cl(CO)$_2$(PPh$_3$)$_2$]** (**4·CO**) in CD_2Cl_2.

Synthesis of [Ru(CH=CH-BODIPY^{Me,Me})Cl(CO)$_2$(PPh$_3$)$_2$] (5·CO)

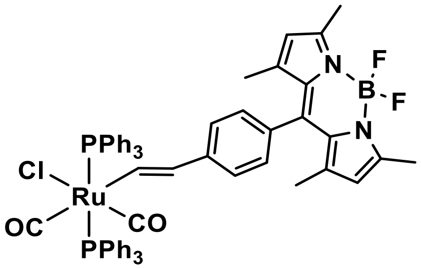

Carbon monoxide was bubbled through a dichloromethane solution (10 mL) of **5** (30 mg, 0.03 mmol) for 30 seconds without a change in color. All solvent was removed and petroleum ether (40-60 °C) (10 mL) was added to the crude product. The suspension was triturated ultrasonically for 10 min and then filtered. The product was isolated as a red powder (26 mg, 92%). IR (solid state): 2028 (CO), 1964 (CO), 1542, 1510, 1434, 1408, 1364, 1308, 1194, 1156, 1090, 980 cm^{-1}. ^{1}H NMR (400 MHz, CD_2Cl_2): δ 7.75 – 7.28 (m, 30H, C_6H_5), 7.40 (d, 1H, J_{HH} = 16.6 Hz, H$_\alpha$), 7.12, 6.98 (AB, 2 x 2H, J_{AB} = 7.9 Hz, C_6H_4), 6.71 (s, 2H, pyrrole-CH), 6.05 (d, J_{HH} = 16.6 Hz, 1H, H$_\beta$), 3.90 (s, 6H, pyrrole-CH$_3$), 1.56 (s, 6H, pyrrole-CH$_3$) ppm. ^{11}B{^{1}H} NMR (128 MHz, CD_2Cl_2): δ 1.14 (t, J_{BF} = 33.3 Hz) ppm. ^{19}F{^{1}H} NMR (377 MHz, CD_2Cl_2): δ -138.3 (q, J_{FB} = 33.3 Hz) ppm. ^{31}P{^{1}H} NMR (162 MHz, CD_2Cl_2): δ 25.0 ppm. MS (ES^{+}-TOF) m/z 1072 (abundance): 1072 (100) [M – Cl + MeCN]$^{+}$, 1066 (5) M^{+}, 1044 (51) [M – Cl – CO + MeCN]$^{+}$. Elemental Analysis: Calculated for $C_{59}H_{50}BClF_2N_2O_2P_2Ru$ (Mw = 1066.32) C, 66.5; H, 4.7; N, 2.6%. Found: C, 66.3; H, 4.8; N, 2.6%.

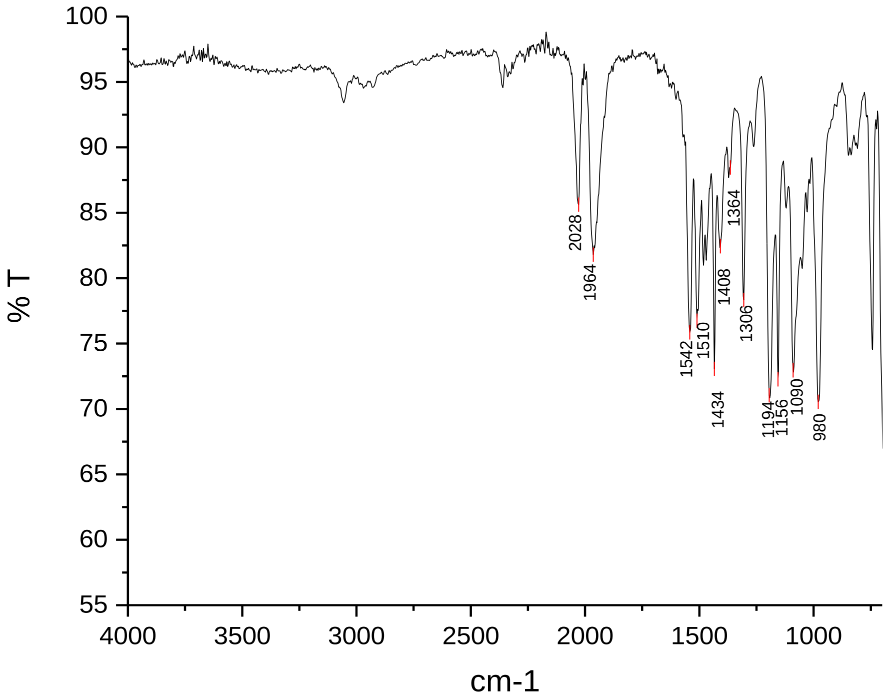

Figure S2-24. Infrared spectrum of **[Ru(CH=CH-BODIPY^{Me,Me})Cl(CO)$_2$(PPh$_3$)$_2$]** (**5·CO**) in the solid state.

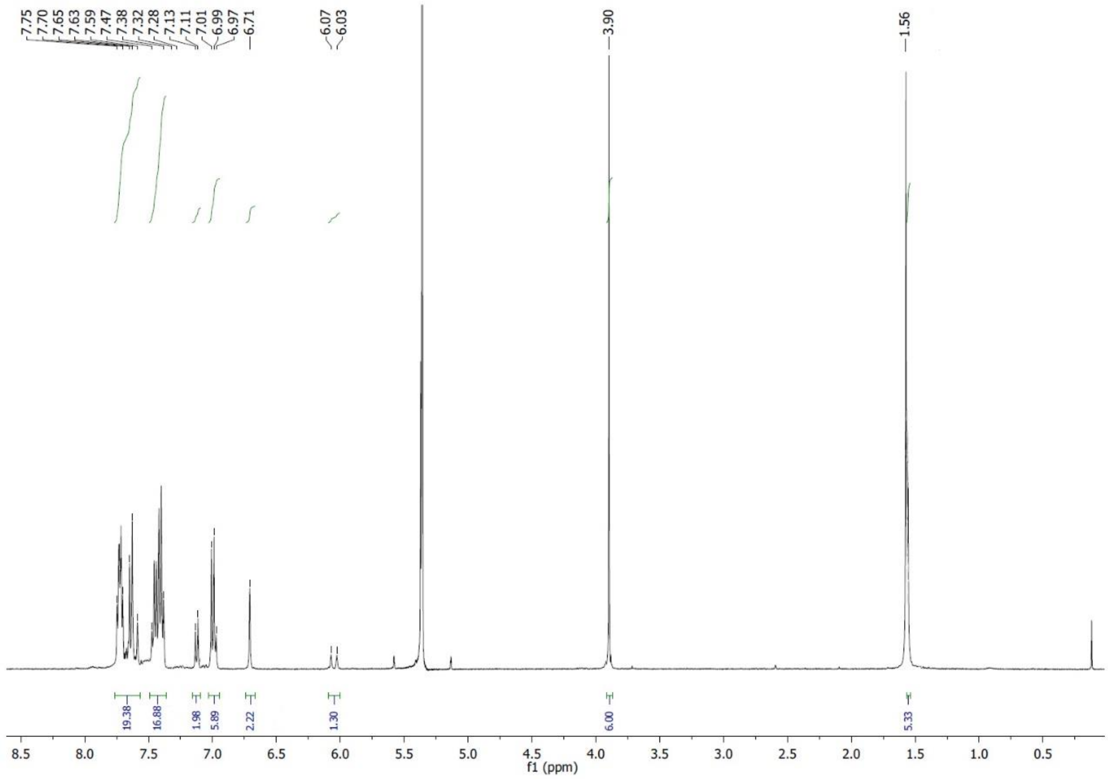

Figure S2-25. ^{1}H NMR spectrum of **[Ru(CH=CH-BODIPY^{Me,Me})Cl(CO)$_2$(PPh$_3$)$_2$]** (**5·CO**) in CD_2Cl_2.

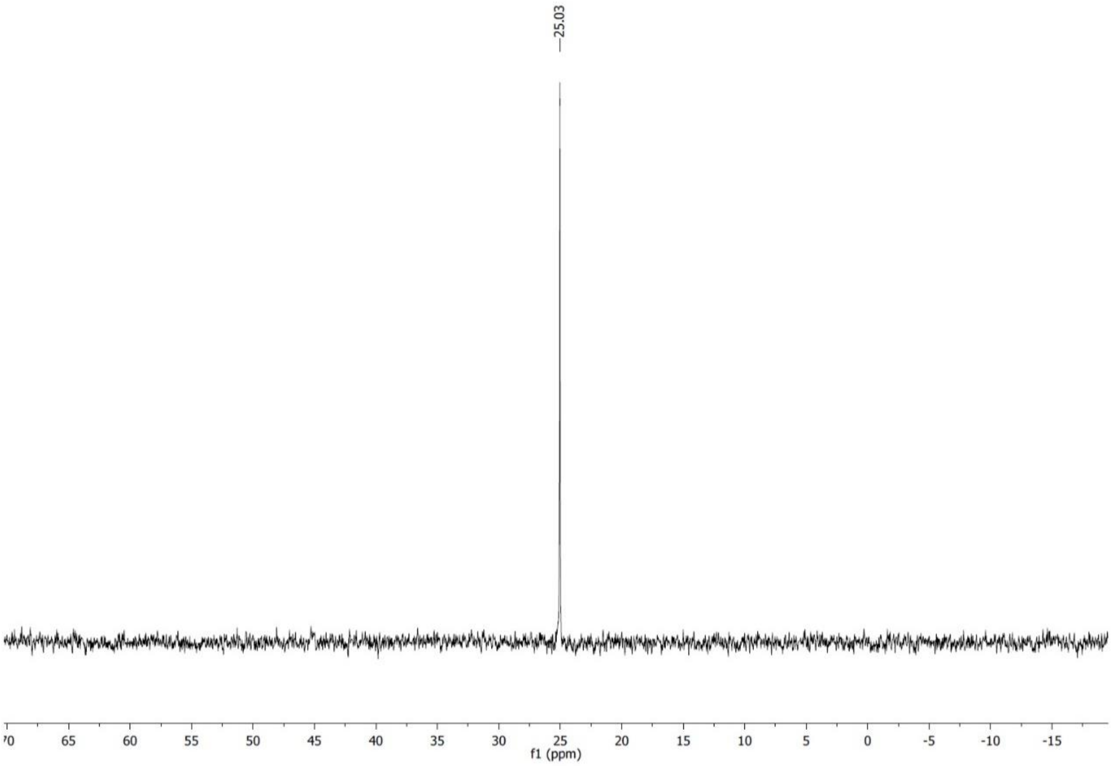

Figure S2-26. $^{31}P\{^{1}H\}$ NMR spectrum of **[Ru(CH=CH-BODIPY^{Me,Me})Cl(CO)$_2$(PPh$_3$)$_2$]** (**5·CO**) in CD_2Cl_2.

Synthesis of [Ru(CH=CH-BODIPY^{Et})Cl(CO)$_2$(PPh$_3$)$_2$] (6·CO)

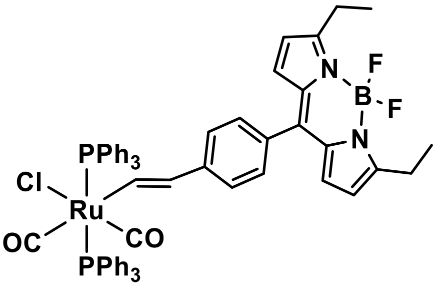

Carbon monoxide was bubbled through a dichloromethane solution (10 mL) of **6** (50 mg, 0.048 mmol) until the solution turned from red to orange (20 seconds). All solvent was removed and petroleum ether (40-60 °C) (10 mL) was added to the crude product. The suspension was triturated ultrasonically for 10 minutes and then filtered. The product was isolated as a red powder (48 mg, 94%). IR (solid state): 2031 (CO), 1969 (CO), 1556, 1484, 1434, 1319, 1194, 1138, 1033, 740 cm^{-1}. ^{1}H NMR (400 MHz, CD_2Cl_2): δ 7.87 (dt, 1H, J_{HH} = 18.0, J_{HP} = 3.5 Hz, H$_\alpha$), 7.73, 7.42 (m x 2, 30H + 2H, C_6H_5 + C_6H_4), 6.99 (d, 2H, J_{HH} = 8.0 Hz, C_6H_4), 6.90 (d, 2H, J_{HH} = 4.2 Hz, pyrrole-CH), 6.46 (d, 2H, J_{HH} = 4.2 Hz, pyrrole-CH), 6.06 (dt, 1H, J_{HH} = 18.1, J_{HP} = 2.1 Hz, H$_\beta$), 3.09 (q, 4H, J_{HH} = 7.6 Hz, pyrrole-CH_2), 1.38 (t, 6H, J_{HH} = 7.6 Hz, pyrrole-CH_3) ppm. ^{11}B{^{1}H} NMR (128 MHz, CD_2Cl_2): δ 0.95 (t, J_{BF} = 32.9 Hz) ppm. ^{13}C{^{1}H} NMR (101 MHz, CD_2Cl_2): δ 163.6 (t, J_{CP} = 14.2 Hz, CO), 162.7 (s, BODIPY-C8), 143.8 (s, C_6H_4), 142.4 (s, BODIPY), 136.8 (s(br), Cα), 134.2 (t^v, J_{CP} = 5.2 Hz, *o/m*-C_6H_5), 134.1 (s, BODIPY), 134.0 (s, C_6H_4), 132.5 (t^v, J_{CP} = 23.5 Hz, *ipso*-C_6H_5), 130.7 (s, *o/m*-C_6H_4), 130.5 (s, BODIPY), 130.3 (s, *p*-C_6H_5), 128.1 (t^v, J_{CP} = 4.8 Hz, *o/m*-C_6H_5), 128.0 (s(br), Cβ), 124.3 (s, *o/m*-C_6H_4), 116.9 (s, BODIPY), 22.0 (s, BODIPY-CH_2), 12.6 (s, BODIPY-CH_3) ppm. ^{19}F{^{1}H} NMR (377 MHz, CD_2Cl_2): δ -145.24 (q, J_{FB} = 32.5 Hz) ppm. ^{31}P{^{1}H} NMR (162 MHz, CD_2Cl_2): δ 24.4 ppm. MS (ES$^+$-TOF) *m/z* (abundance): 1066 (6) [M]$^+$, 1044 (51) [M – Cl – CO + MeCN]$^+$. Elemental analysis: Calculated for $C_{59}H_{50}BClF_2N_2O_2P_2Ru{\cdot}0.5\ CH_2Cl_2$ (Mw = 1108.79) C, 64.5; H, 4.6; N, 2.5%. Found: C, 64.9; H, 5.0; N, 2.5%.

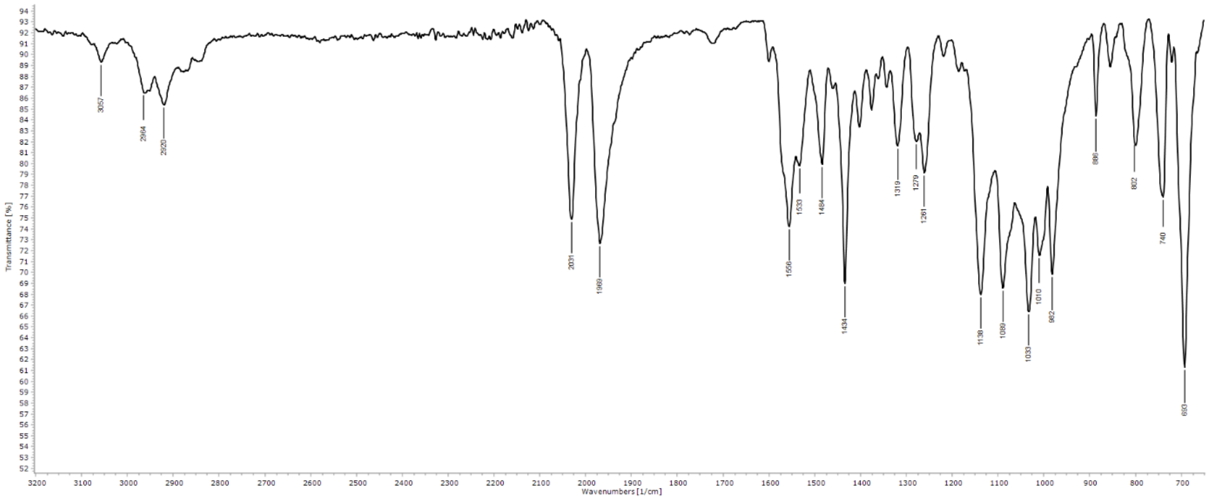

Figure S2-27. Infrared spectrum of **[Ru(CH=CH-BODIPY^{Et})Cl(CO)$_2$(PPh$_3$)$_2$]** (**6·CO**) in the solid state.

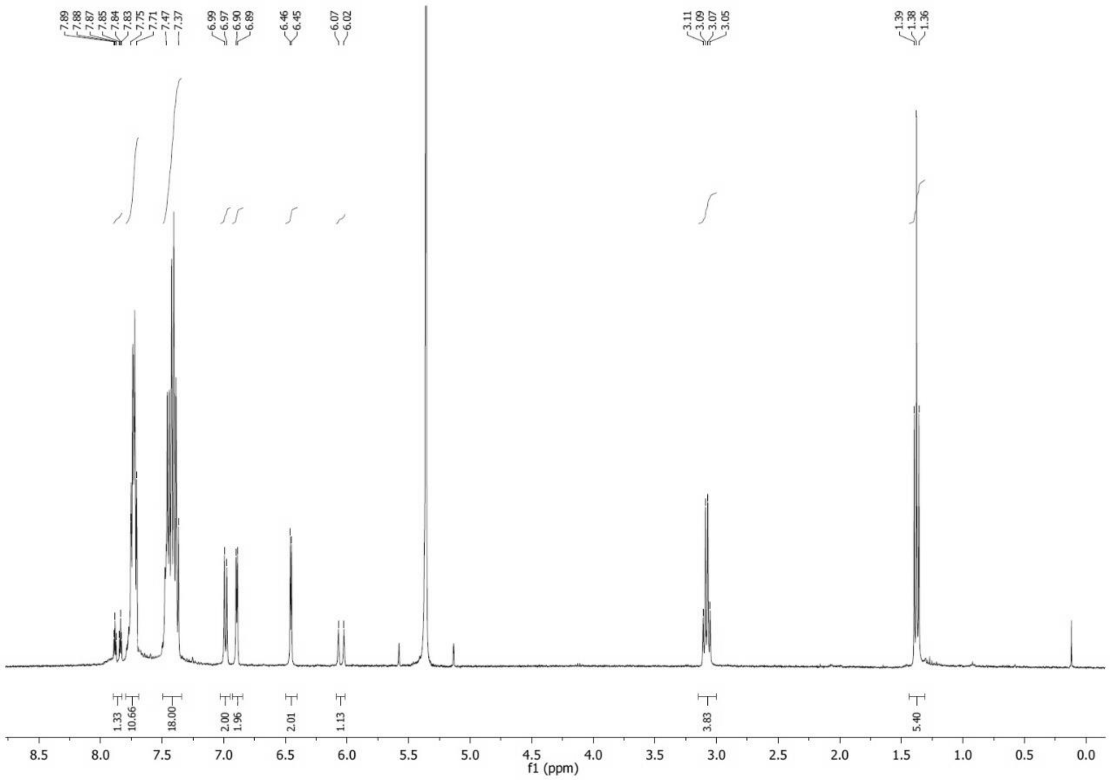

Figure S2-28. ^{1}H NMR spectrum of **[Ru(CH=CH-BODIPY^{Et})Cl(CO)$_2$(PPh$_3$)$_2$]** (**6·CO**) in CD_2Cl_2.

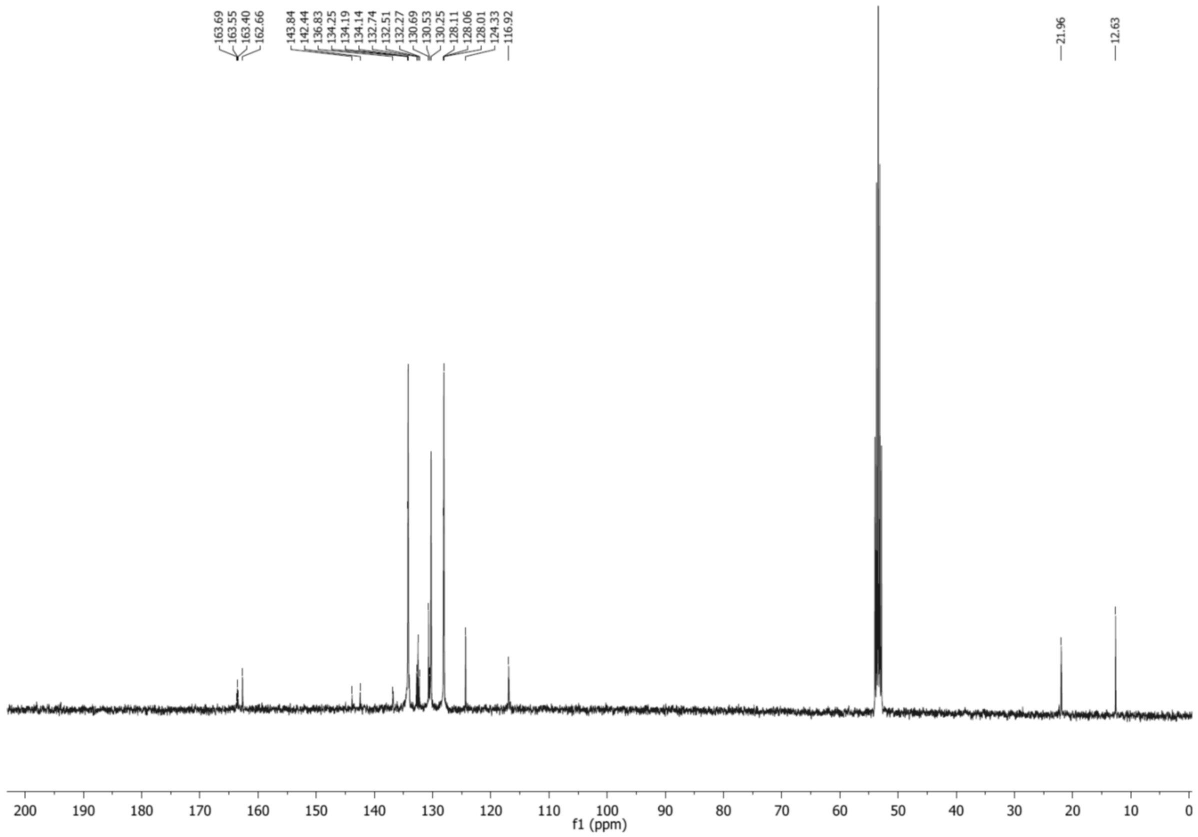

Figure S2-29. ^{13}C{^{1}H} NMR spectrum of **[Ru(CH=CH-BODIPY^{Et})Cl(CO)$_2$(PPh$_3$)$_2$]** (**6·CO**) in CD_2Cl_2.

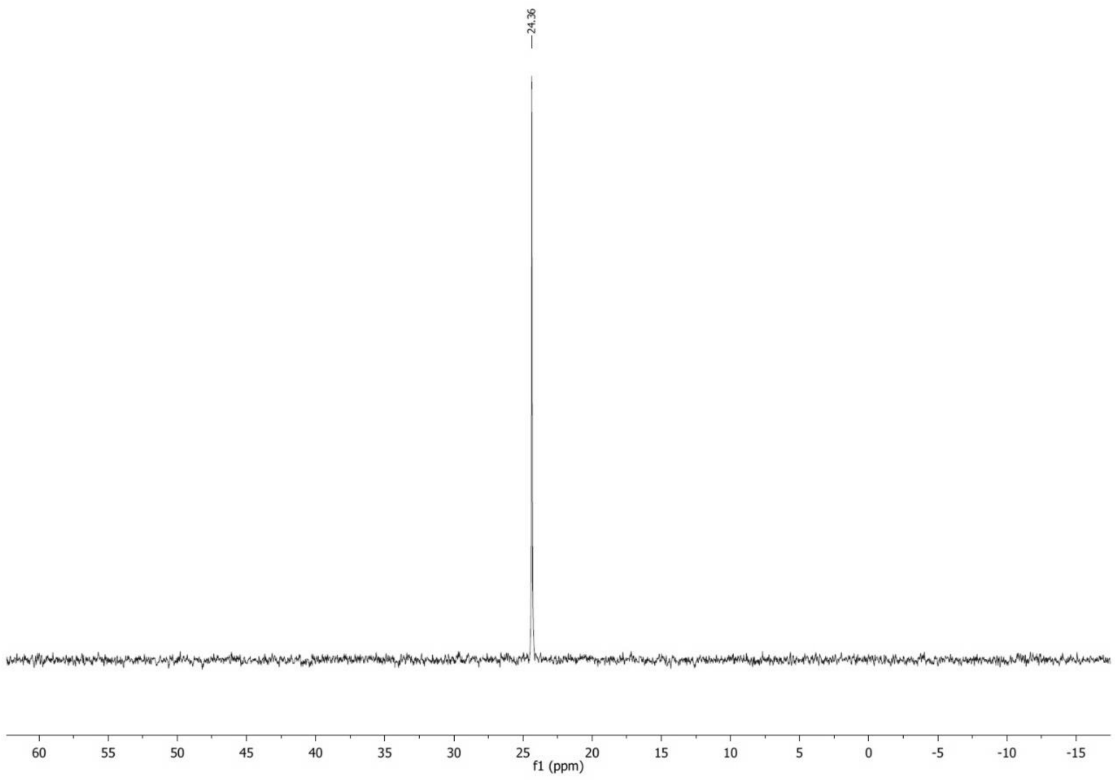

Figure S2-30. $^{31}P\{^{1}H\}$ NMR spectrum of **[Ru(CH=CH-BODIPY^{Et})Cl(CO)$_2$(PPh$_3$)$_2$]** (**6·CO**) in CD_2Cl_2.

S3 Photophysical measurements of ethynyl-BODIPY compounds

General Observations

Fluorophores **1 – 3** have different emission properties based on the substitution of the BODIPY core. The most substituted fluorophore **1** has the highest quantum yield (QY) and **3** has the lowest QY due to free rotation of the phenyl group. The quantum yield of the probes **4 – 6** is substantially reduced compared to free fluorophores, showing quenching of the fluorescence. With the addition of CO, **4·CO** - **6·CO** produced similar quantum yield to that of the free fluorophore, restoring the emission.

It is evident from our data that emission intensity of all the CO adducts is significantly enhanced compared to the parent complexes. This enhancement does not appear to affect the fluorescence lifetime (see Figure S8-1). We conclude that the radiative and non-radiative *decay* rates from the fluorescent excited state are not affected by the metal centre (with or without CO), however, *the population* of this excited state is significantly affected. On the basis of the fluorescent lifetime of all complexes being very close to that of the free fluorophores,[S8] we hypothesise that the BODIPY ligand essentially acts as an independent organic fluorophore. This conclusion is in line with the work of Winter and co-workers, who reported ligand-dominated HOMO for related ruthenium vinyl complexes.[S9]

The quantum yields measured for the CO adducts are also very similar to what would be expected for the respective free BODIPY ligands,[S10] while in **4**-**6** these values are significantly reduced (Figures S3-4 and S3-5). Without detailed DFT calculations, this effect cannot be attributed confidently, however, we hypothesise that it may be due to a structural *trans*-influence of the CO ligand, which changes the balance in populations between the bright organic fluorophore excited state and the d-d metal states, similar to the situation reported by Winter et al.[S9]

Quantum Yield data could be used as a guide to show that the addition of CO increases the quantum yield but that this does not surpass the quantum yield of the free fluorophore. It is noted that the quantum yield values of **4**-**6** are very low and significantly lower than that of the standard used. The technique used to calculate quantum yields can be inconsistent under such conditions. Strictly quantifiable enhancement of fluorescence with addition of CO is detailed in Section S4 (detection limit and fluorescence response).

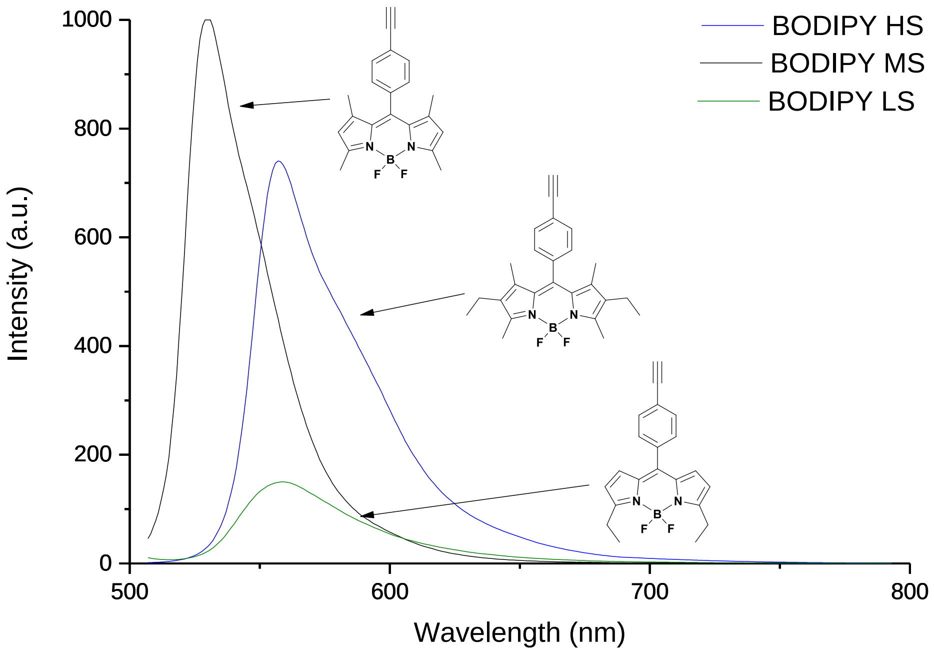

Figure S3-1. Emission spectra of three ethynyl-BODIPY fluorophores **1** – **3** (5 µM) in dichloromethane (HS = high substitution, MS = medium substitution, LS = low substitution).

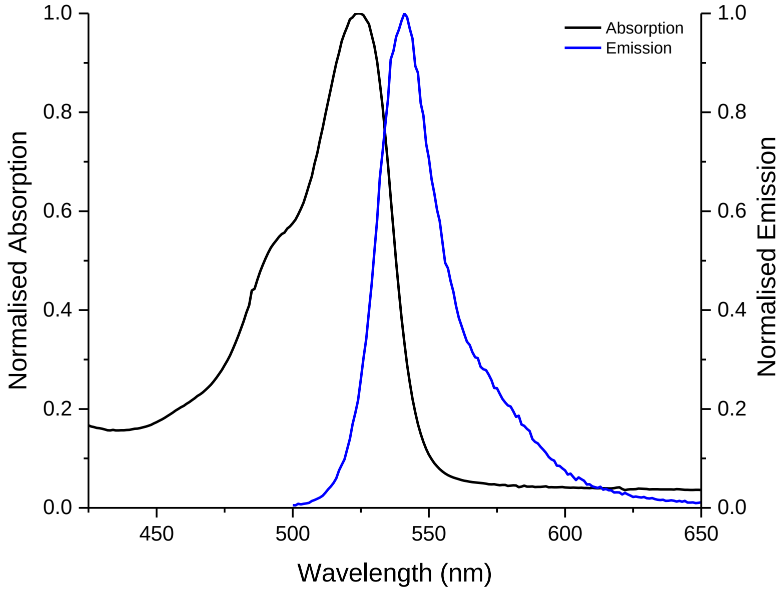

Figure S3-2. Absorption and Emission spectra of **4** (5 µM) in dichloromethane, excitation at 480 nm. Note that two absorption peaks are often observed for such BODIPY fluorophores.[S11]

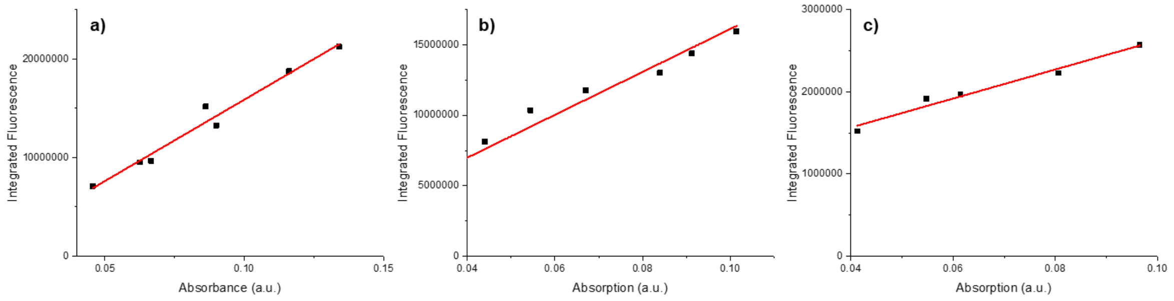

Figure S3-3. Integrated fluorescence *vs.* absorbance: a) Compound **1**, Grad = 1.65 x 10^8, Φ = 0.86; b) Compound **2**, Grad = 1.52 x 10^8, Φ = 0.79; c) Compound **3**, Grad = 1.77 x 10^7, Φ = 0.06.

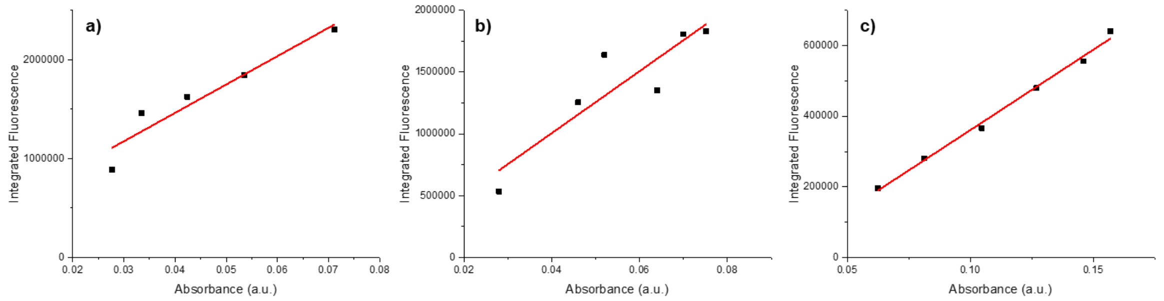

Figure S3-4. Integrated fluorescence *vs.* absorbance: a) Compound **4**, Grad = 3.39 x 10^7, Φ = 0.10; b) Compound **5**, Grad = 2.58 x 10^7, Φ = 0.08; c) **6** – Grad = 3.25 x 10^6, Φ = 0.009.

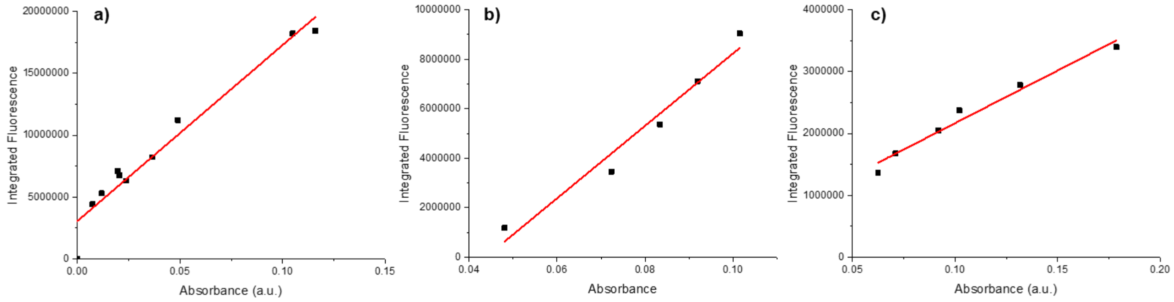

Figure S3-5. Integrated fluorescence *vs.* absorbance: a) Compound **4•CO**, Grad = 1.48 x 10^8, Φ = 0.77; b) Compound **5•CO**, Grad = 1.45 x 10^8, Φ = 0.76; c) Compound **6•CO**, Grad = 1.70 x 10^7, Φ = 0.06.

S4 Carbon monoxide detection in solution

General Observations

Probes **4 – 6** all provided sensitive and selective detection of CO in solution. Due to the low detection limit of **4** and the high quantum yield of **4·CO**, this was the preferred probe for *in vitro* studies, allowing changes in cellular CO concentration to be easily visualised. Although probe **5** also provided an enhancement in fluorescence with CO, it was outperformed by **4** and therefore not taken forward into biological studies. Neither **4** nor **5** could detect changes in viscosity therefore **6** was used to monitor both cellular viscosity and CO in biological studies.

In solution studies, the change in fluorescence with addition of CO is labelled F/F_0 where F is the fluorescence intensity upon treatment with CO and F_0 is the fluorescence intensity of the control sample without CO.

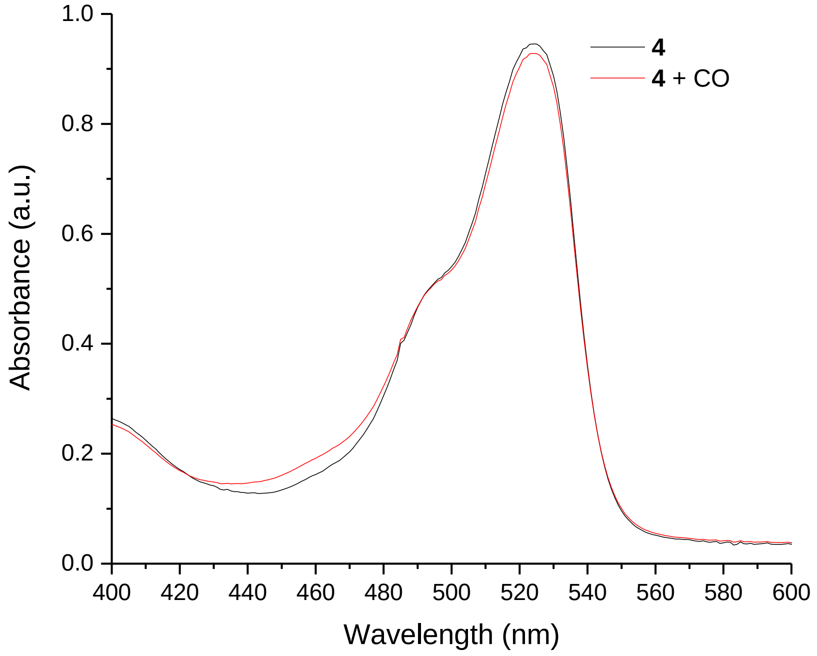

Figure S4-1. Absorption spectra of **4** (5 µM) and **4** (5 µM) saturated with CO in dichloromethane. The fact that there is no spectral change following addition of CO is typical for all complexes studied.

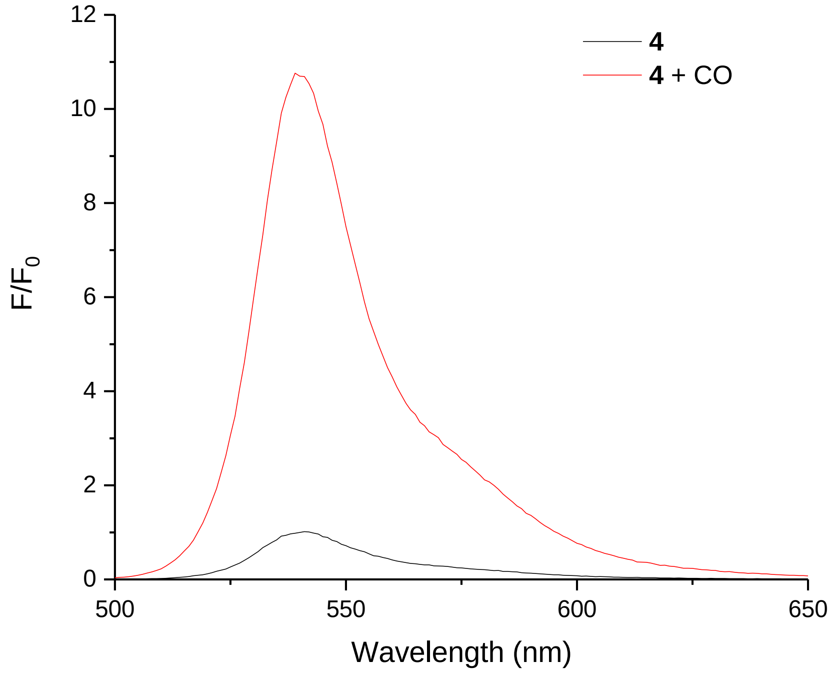

Figure S4-2. Emission spectra of **4** (10 µM) and **4** (10 µM) saturated with CO in dichloromethane, excitation at 480 nm.

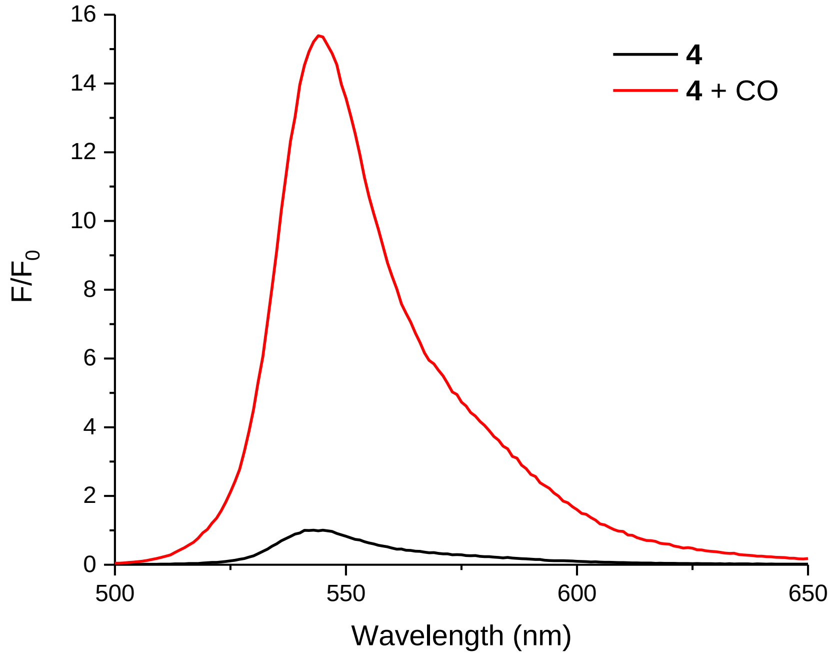

Figure S4-3. Emission spectra of **4** (10 µM) and **4** (10 µM) saturated with CO in pH 7.4, 25 mM PBS–DMSO (9:1 v/v) solution with excitation at 480 nm.

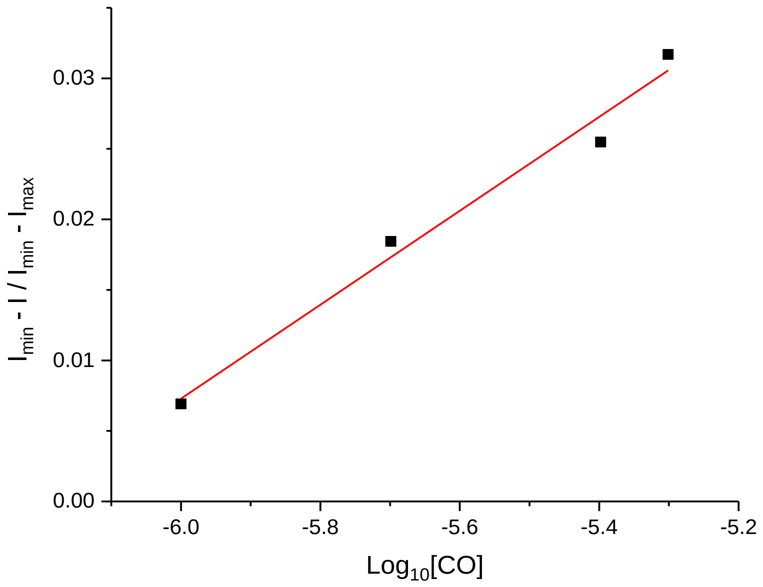

Figure S4-4. Detection limit: Probe **4** (10 µM in pH 7.4, 25 mM PBS–DMSO (9:1 v/v) solution) exposed to low concentrations of CORM-2 (0 – 5 µM). Normalised fluorescence intensity demonstrates a linear relationship with log_{10}[CO], therefore this was fitted to a line with the equation y = mx + c, where m = 0.033 and c = 0.207; R^2 = 0.98.

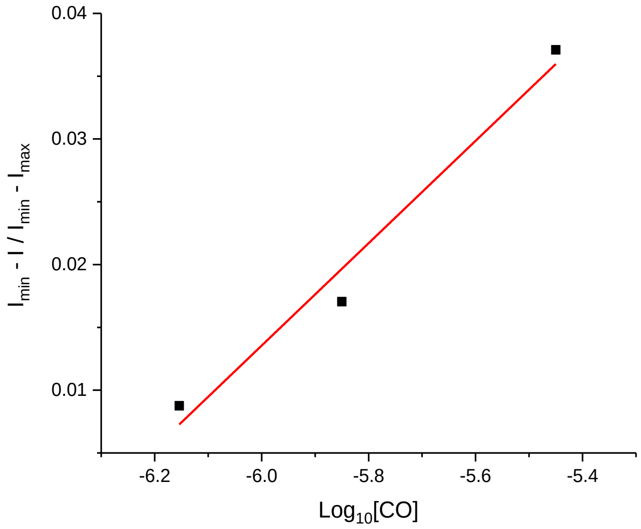

Figure S4-5. Detection limit: Probe **6** (10 µM in pH 7.4, 25 mM PBS–DMSO (9 : 1 v/v)) incubated with small concentrations of CORM-2 (0 – 5 µM). Normalised fluorescence intensity demonstrates a linear relationship with log_{10}[CO], therefore fitted to a line with the equation y = mx + c, where m = 0.040 and c = 0.258; R^2 = 0.98.

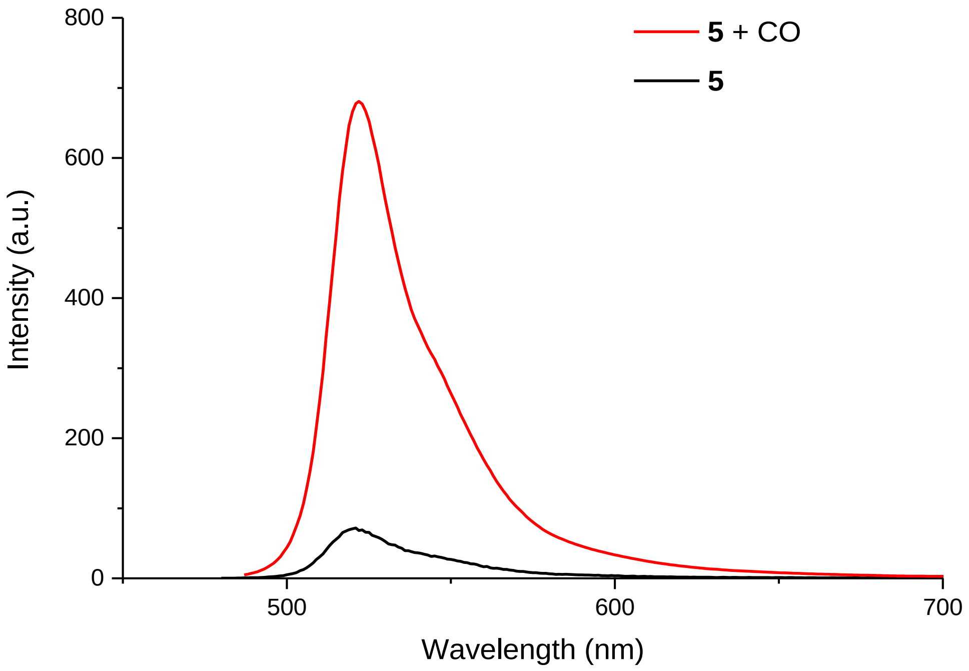

Figure S4-6. Emission spectra of **5** (10 μM) and **5** (10 μM) saturated with CO in dichloromethane (excitation at 480 nm).

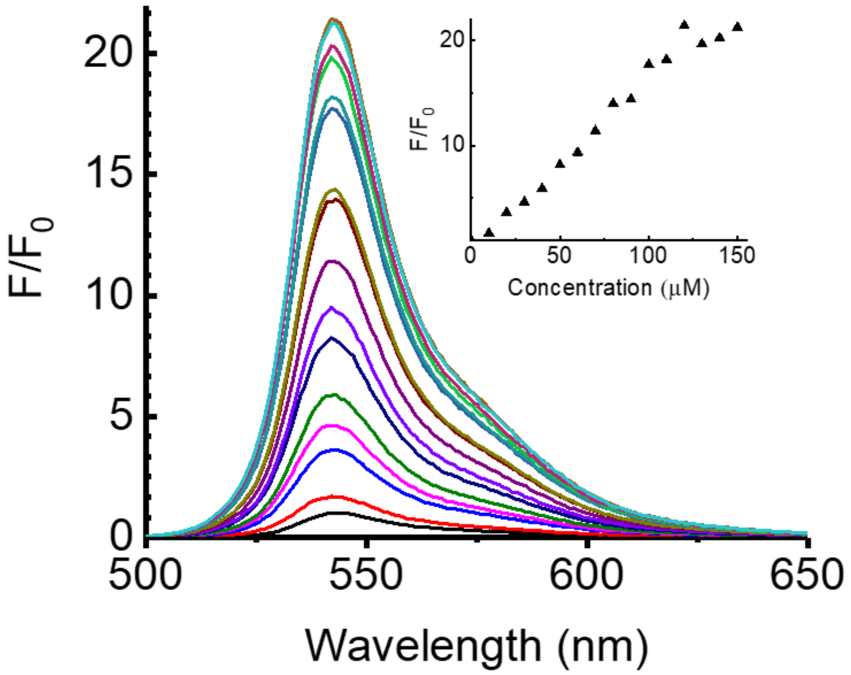

Figure S4-7. The fluorescence response of **4** (10 μM in pH 7.4, 25 μM PBS–DMSO (9:1 v/v) solution) upon addition of 0 - 150 μM of CORM-2; λ_{ex} = 480 nm; insert shows change in fluorescence intensity at 550 nm.

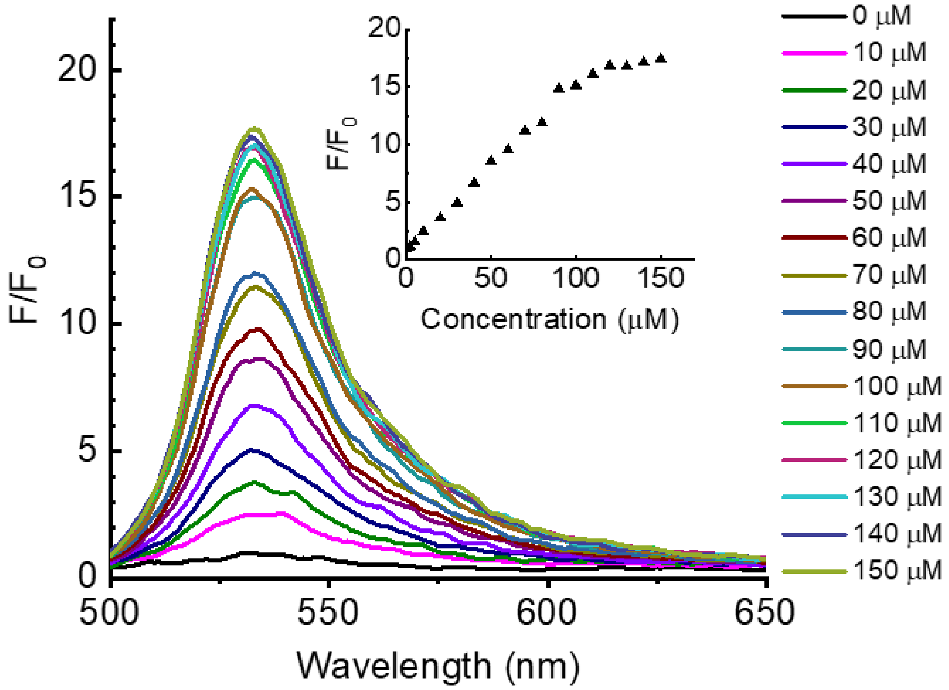

Figure S4-8. Fluorescence response of **6** (10 µM, pH 7.4, 25 mM PBS–DMSO (9:1 v/v) solution) with 0 - 150 µM CORM-2; λ_{ex} = 480 nm; insert shows change in fluorescence intensity at 535 nm.

S5 Carbon monoxide detection in cells

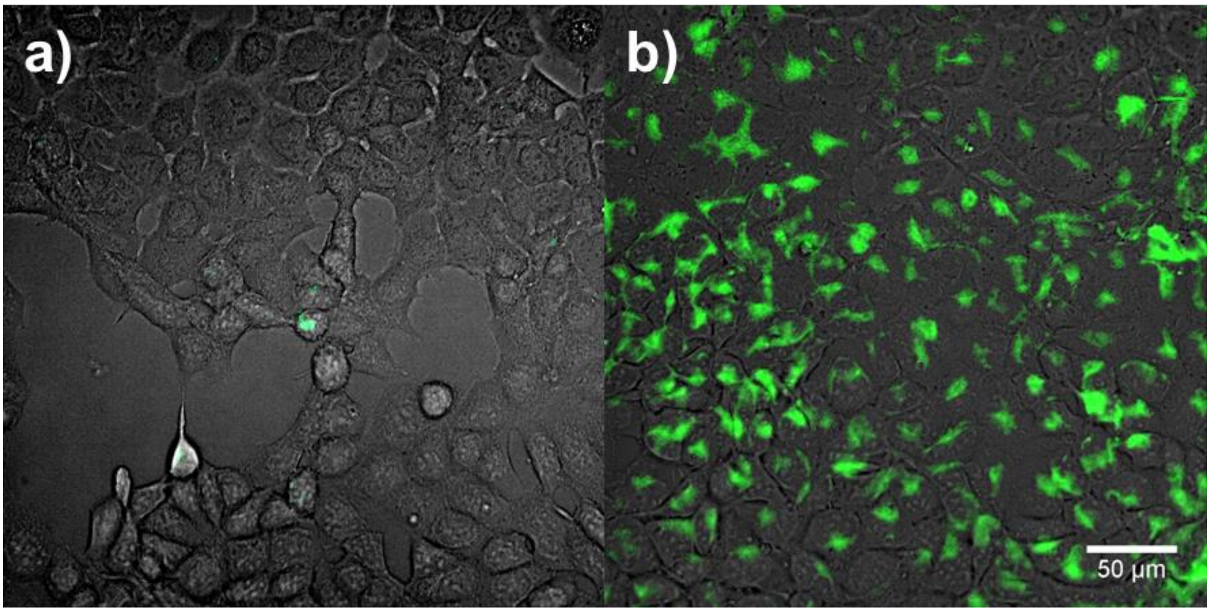

Figure S5-1. Fluorescence and brightfield images of live HEK293 cells incubated with **4** (10 µM, λ_{ex} = 465, λ_{em} = 520 - 560 nm) for 10 min and then (a) 0 µM, (b) 100 µM CORM-2 for 30 min. Scale bar representative for both images.

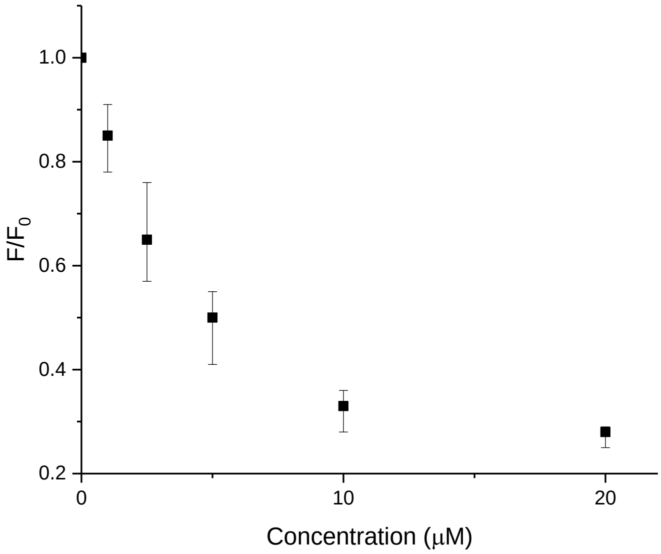

Figure S5-2. Change in fluorescence intensity of cells with ZnPP (0 - 20 µM) compared to control cells using ImageJ software. Data is expressed as mean ±SEM of at least three independent experiments. Excitation at 465 nm.

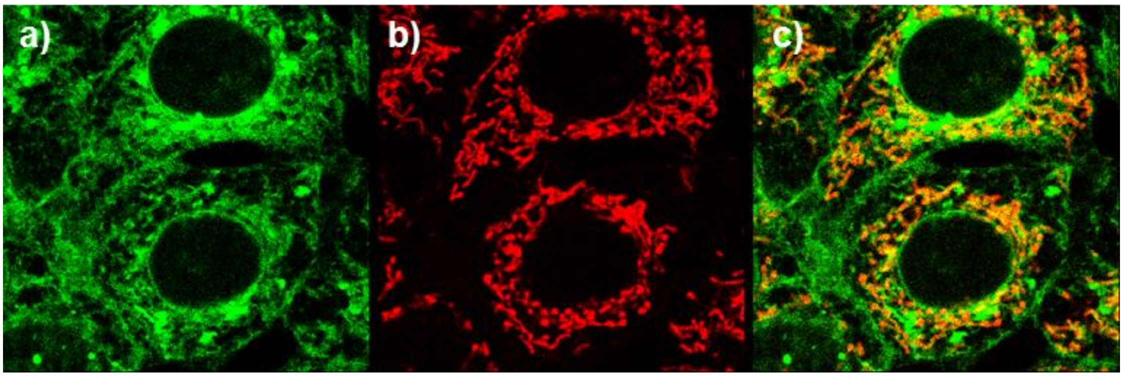

Figure S5-3. Confocal fluorescence image of live MCF-7 cells: (a) **4** (10 µM, λ_{ex} = 465, λ_{em} = 520 - 560 nm), (b) MitoTracker Deep Red^{TM} (15 µM, λ_{ex} = 640, λ_{em} = 665 – 800 nm), (c) overlay of (a)-(b). Fluorophores were incubated in MCF-7 cells with **4** (10 µM) for 10 min.

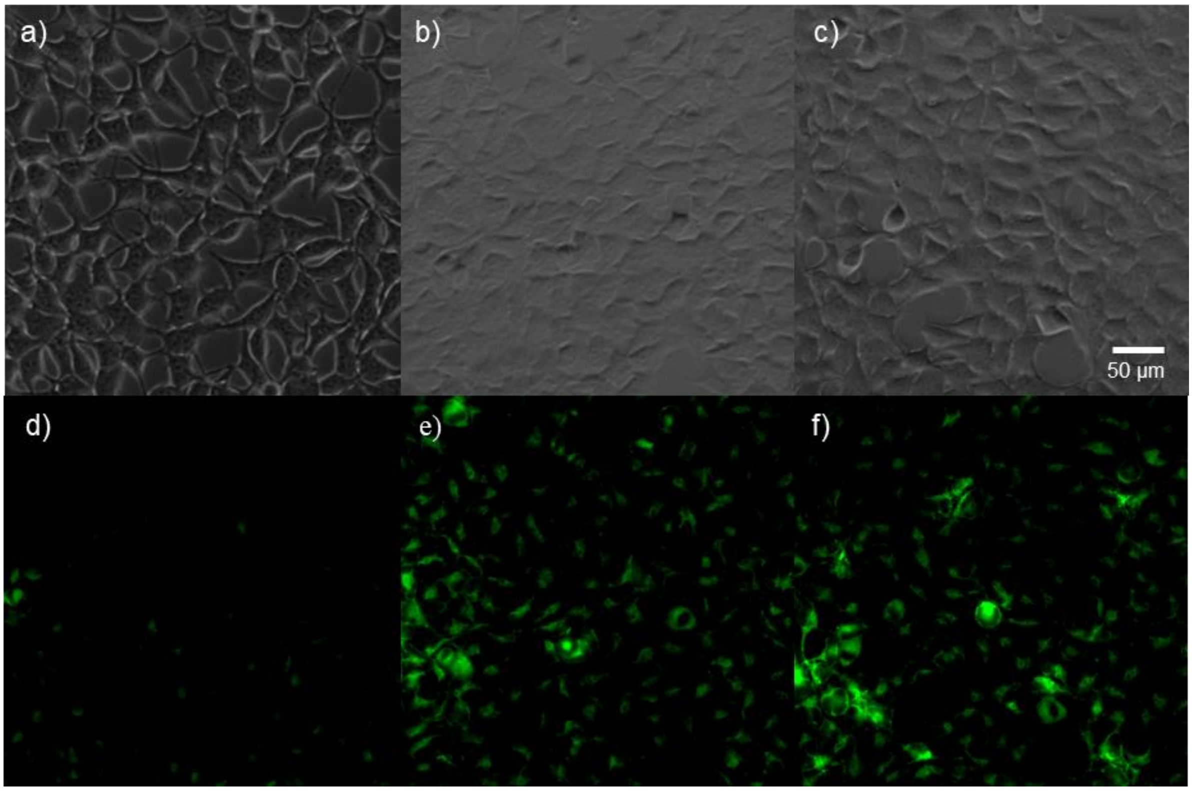

Figure S5-4. Single-photon fluorescent images: (a) bright-field image of live HEK293 cells incubated with **4** (10 µM, λ_{ex} = 465, λ_{em} = 520 - 560 nm) for 10 min; (d) fluorescence image of (a); (b) bright-field image of live HEK293 cells incubated with CORM-3 (50 µM) for 30 min, then with **4** (10 µM) for 10 min; (e) fluorescence image of (b); (c) bright-field image of live HEK293 cells incubated with CORM-3 (100 µM) for 30 min, then with **4** (10 µM) for 10 min; (f) fluorescence image of (c). Scale bar representative for all images.

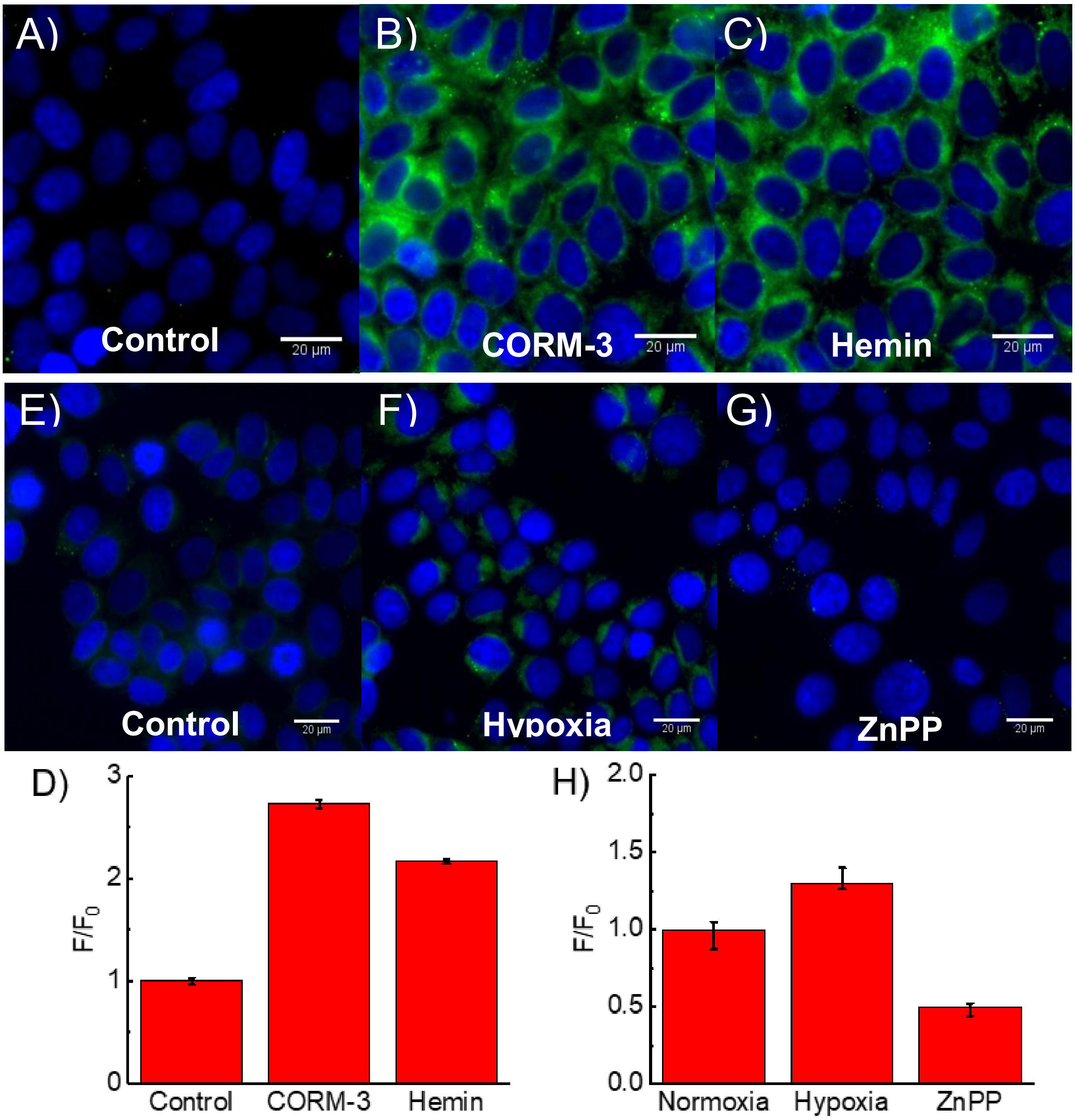

Figure S5-5. Fluorescence images of **4** (10 µM) (green) and Hoechst 33342 (blue) in MCF-7 cells: A) control - untreated, B) 100 µM CORM-3 for 30 min, C) 100 µM hemin for 5 h, E) Control - pre-incubated in a normoxia incubator (37 °C, 5% CO_2, 95% air) for 24 h, F) pre-incubated in a hypoxia incubator (37 °C, 5% CO_2, 1% O_2 / 99% N_2) for 24 h, G) pre-incubated with ZnPP (20 µM) in a hypoxia incubator for 12 h. Integrated change in fluorescence intensity for A, B, C is shown in D and for E, F, G in H (F_0 is fluorescence intensity in control or normoxia and F is fluorescence intensity upon treatment), data expressed as mean ±SEM of at least three independent experiments. A-D live cells; E-H fixed cells.

S6 Stability and competition measurements

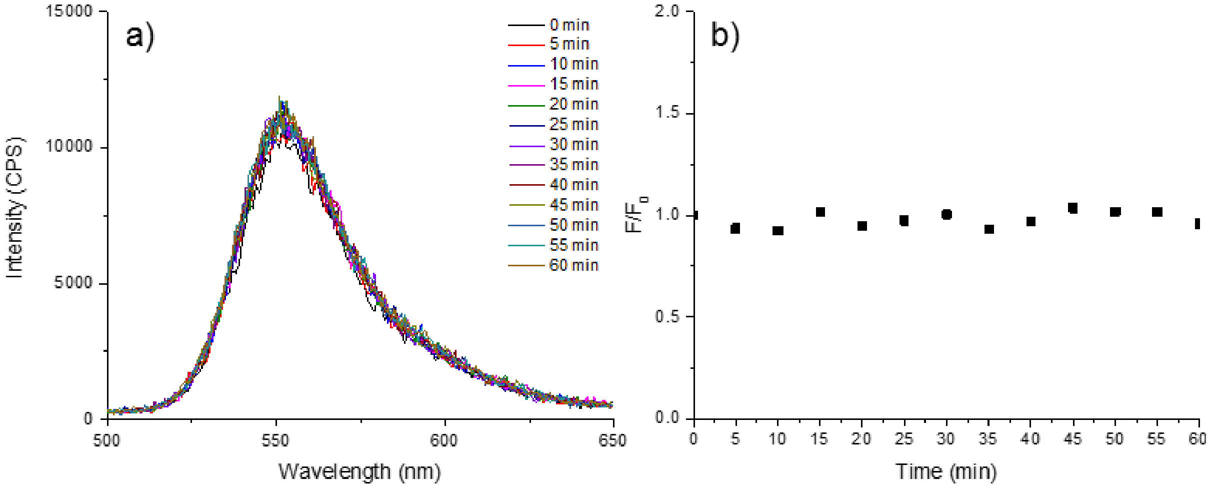

Figure S6-1. Stability study in solution: a) emission spectra of **4** (10 µM) taken in 5 minute intervals for 60 min, in pH 7.4, 25 mM PBS–DMSO (9:1 v/v) solution with excitation at 480 nm; b) change in maximum fluorescence intensity at 550 nm.

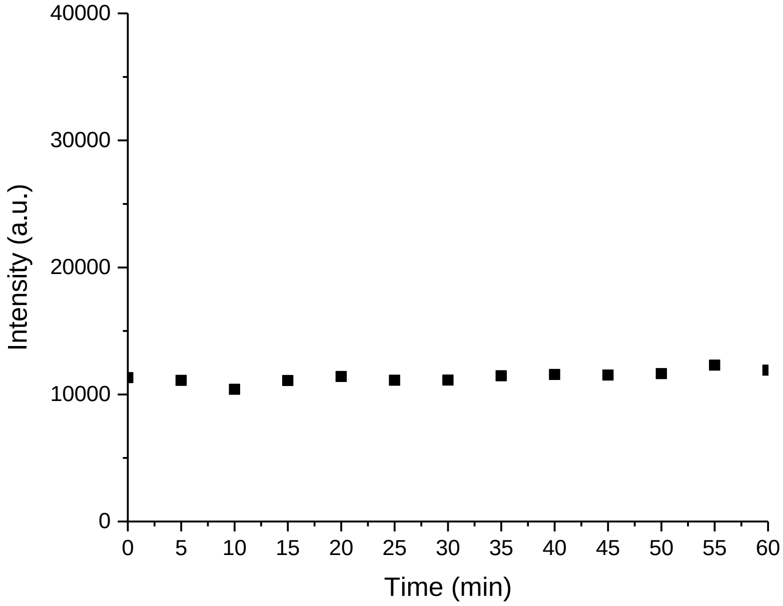

Figure S6-2. UV stability study showing maximum fluorescence intensity of **4** (10 µM) in the presence of UV-irradiation (365 nm) taken at 5 minute intervals for 60 min, in pH 7.4, 25 mM PBS–DMSO (9:1 v/v) with excitation at 480 nm.

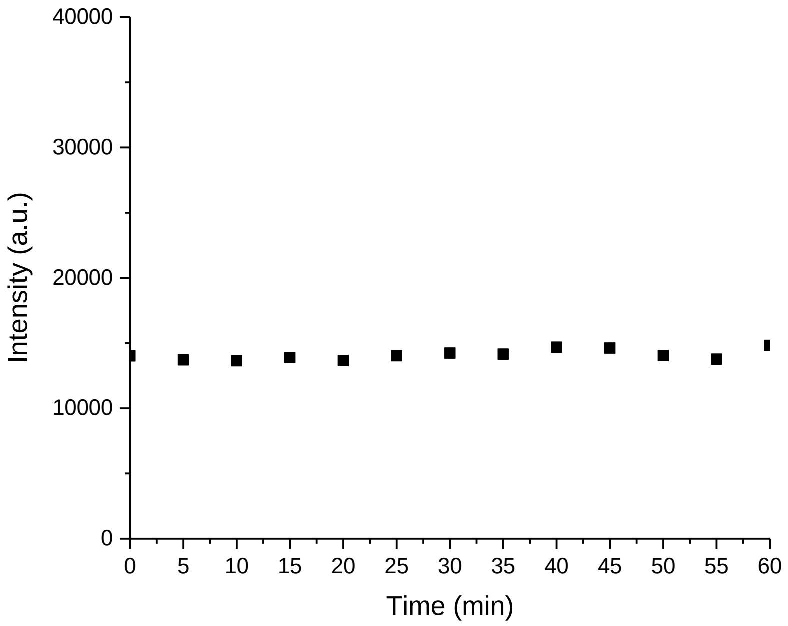

Figure S6-3. ROS stability study showing the maximum fluorescence intensity of **4** (10 µM) in the presence of H_2O_2 (100 µM) taken at 5 minute intervals for 60 min, in pH 7.4, 25 mM PBS–DMSO (9:1 v/v) solution with excitation at 480 nm.

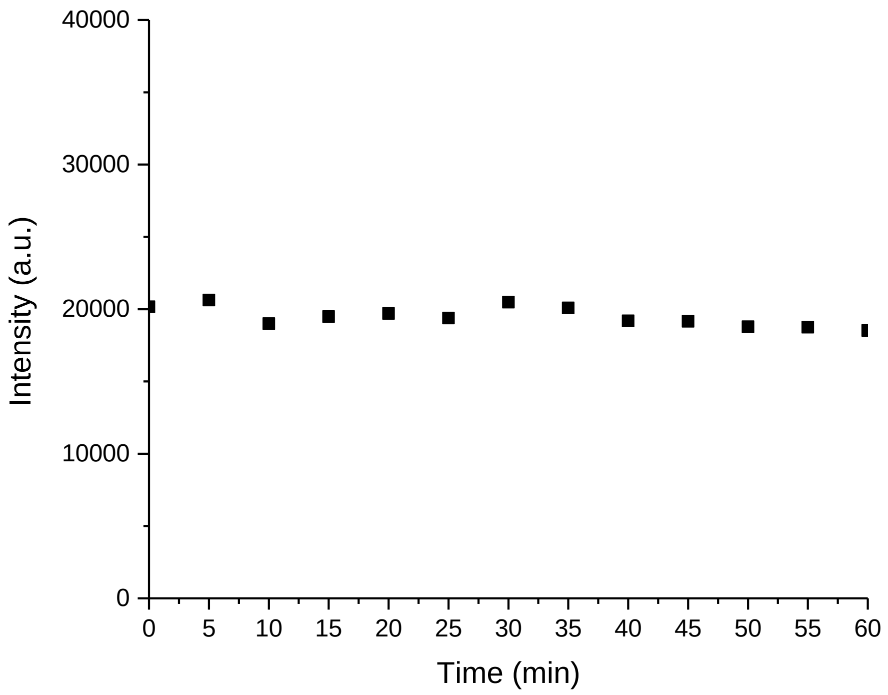

Figure S6-4. Serum stability study showing maximum fluorescence intensity of **4** (10 µM) in the presence of BSA (100 µM) taken at 5 minute intervals for 60 min, in pH 7.4, 25 mM PBS–DMSO (9:1 v/v) solution with excitation at 480 nm.

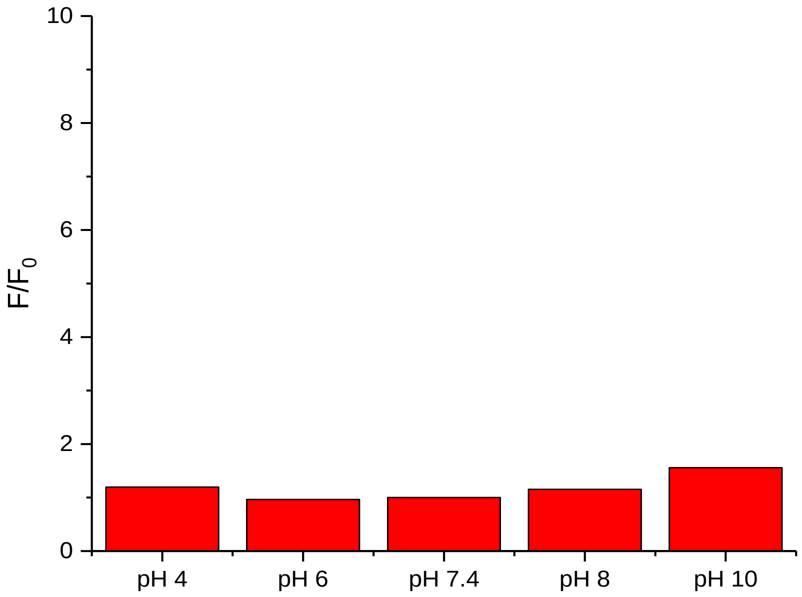

Figure S6-5. pH stability study demonstrating the change in maximum fluorescence intensity of **4** (10 µM) at different pH values in 25 mM PBS–DMSO (9:1 v/v) solution with excitation at 480 nm.

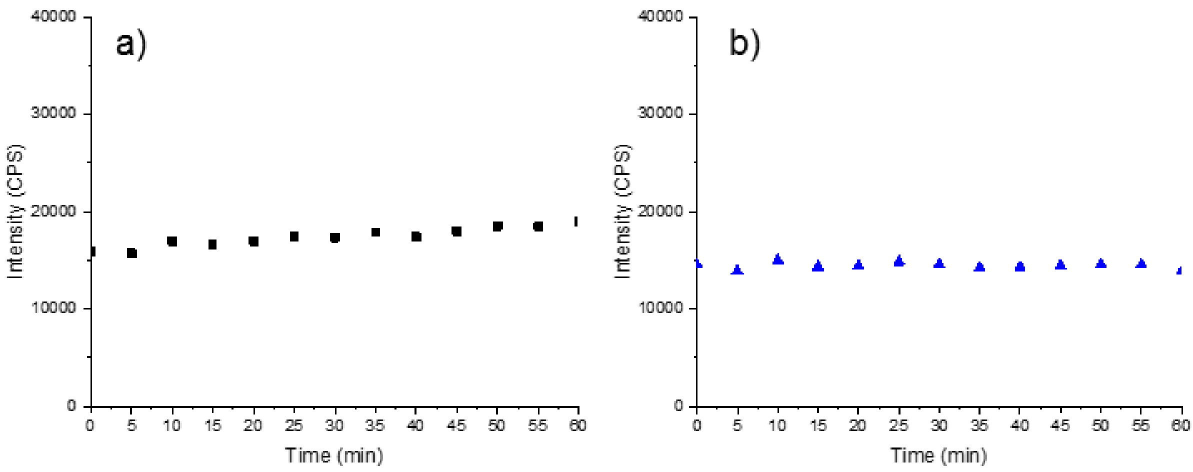

Figure S6-6. Photo stability profiles for **6** (10 µM in pH 7.4, 25 mM PBS–DMSO (9:1 v/v) solution) in the absence (■) or presence (▲) of UV-irradiation at 365 nm; the fluorescence intensities at 550 nm were monitored at 5 minute intervals.

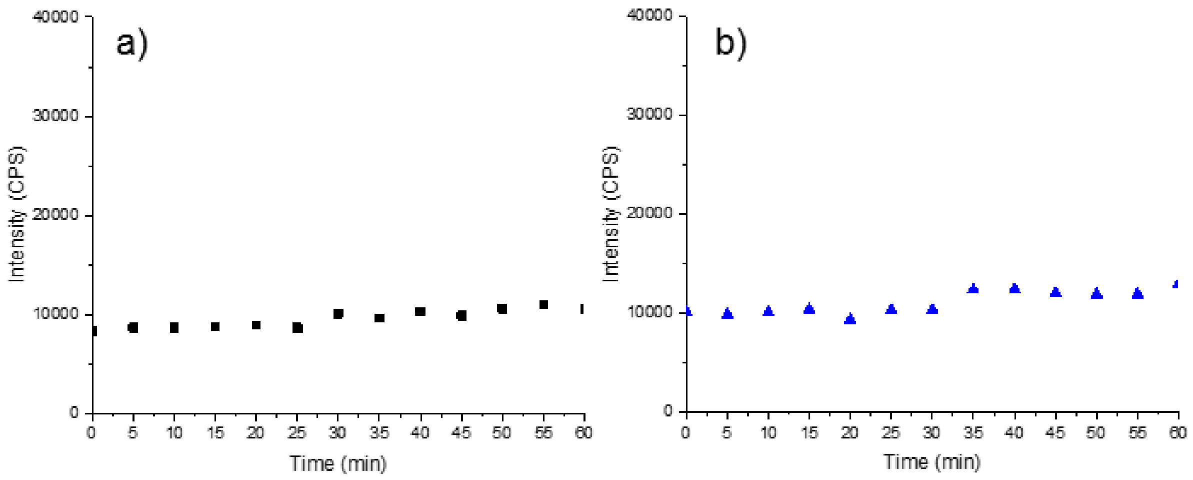

Figure S6-7. Stability profiles of **6** (10 μM in pH 7.4, 25 mM PBS–DMSO (9:1 v/v) solution) in the presence of H_2O_2 (100 μM) (■) or presence of BSA (100 μM) (▲); the fluorescence intensities at 550 nm were monitored at 5 minute intervals.

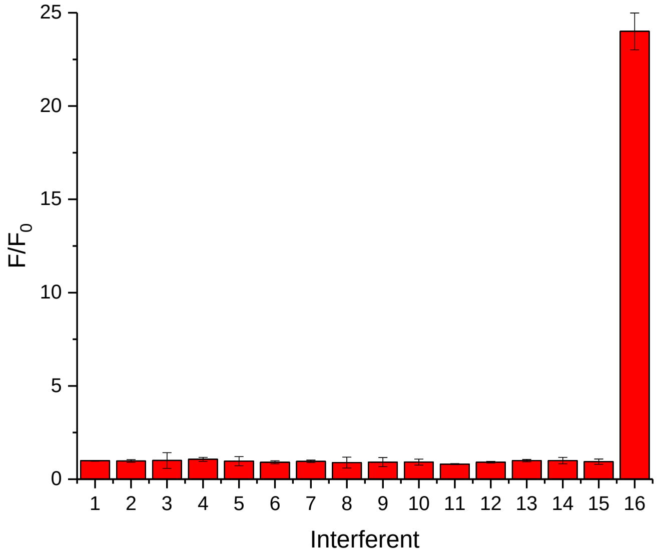

Figure S6-8. The fluorescence response of **4** (10 μM in pH 7.4, 25 mM PBS–DMSO (9:1 v/v) solution) in the presence of various small molecules and biologically relevant species (100 μM): 1. glutathione; 2. cysteine; 3. hemin; 4. BSA; 5. $O_2^{\bullet -}$; 6. OMe^-; 7. imidazole; 8. H_2O_2; 9. SO_3^-; 10. citrate; 11. ClO^-; 12. OH^-; 13. NO_x; 14. ZnPP; 15. sodium dithionite; 16. CO.

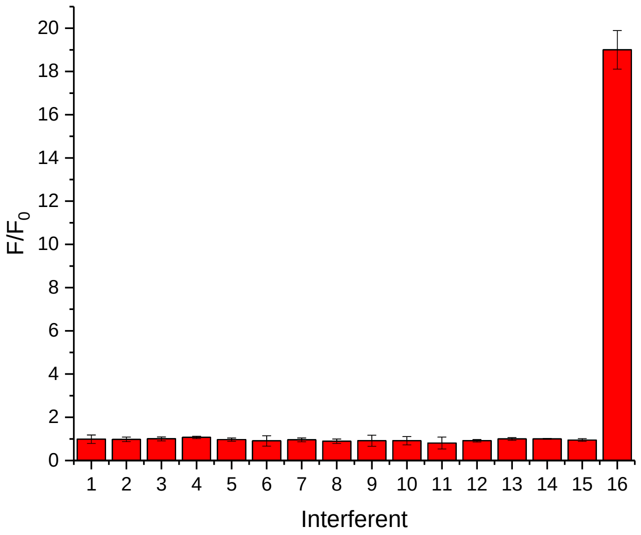

Figure S6-9. The fluorescent response of **6** (10 μM in pH 7.4, 25 mM PBS–DMSO (9 : 1 v/v)) in the presence of various small molecules and biologically relevant species (100 μM): 1. glutathione; 2. cysteine; 3. hemin; 4. BSA; 5. $O_2^{\bullet -}$; 6. OMe^-; 7. imidazole; 8. H_2O_2; 9. SO_3^-; 10. citrate; 11. ClO^-; 12. OH^-; 13. NO_x; 14. ZnPP; 15. sodium dithionite; 16. CO.

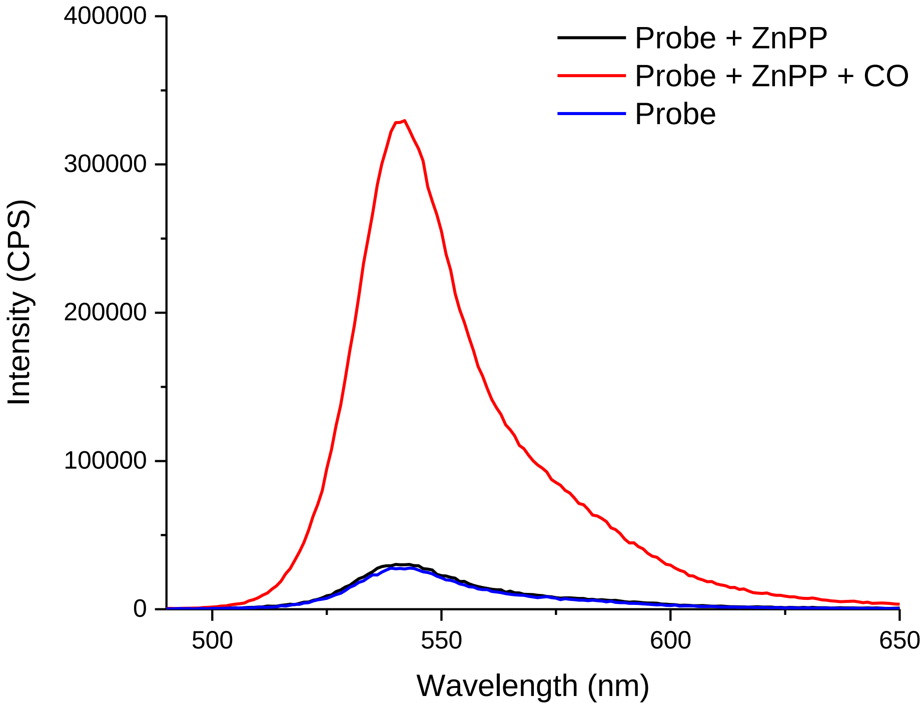

Figure S6-10. The fluorescence response of **4** (10 μM in pH 7.4, 25 mM PBS–DMSO (9:1 v/v) solution) in the presence of ZnPP (100 μM) and CORM-3 (50 μM).

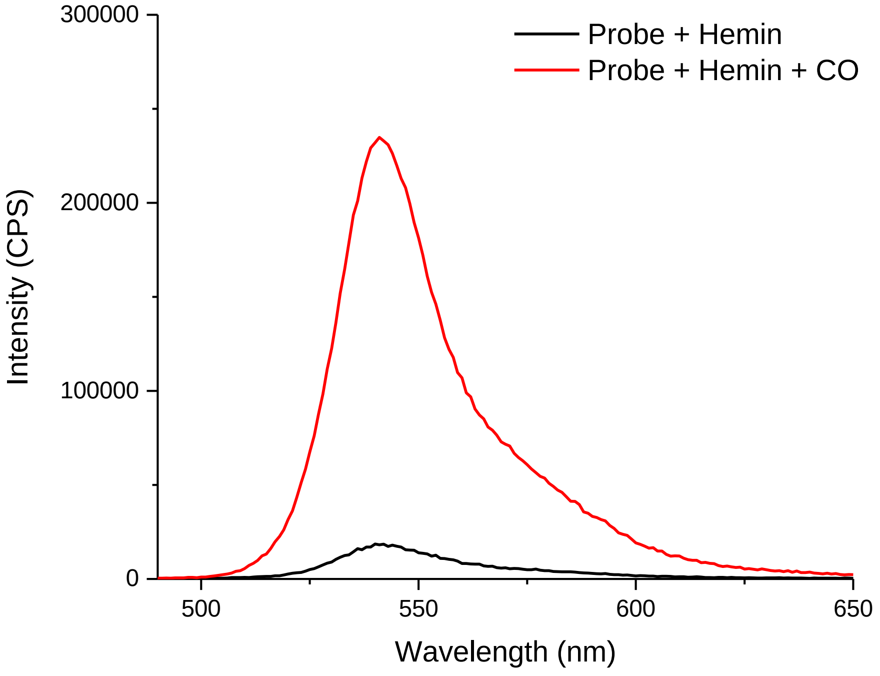

Figure S6-11. The fluorescence response of **4** (10 µM in pH 7.4, 25 mM PBS–DMSO (9:1 v/v) solution) in the presence of Hemin (100 µM) and CORM-3 (50 µM).

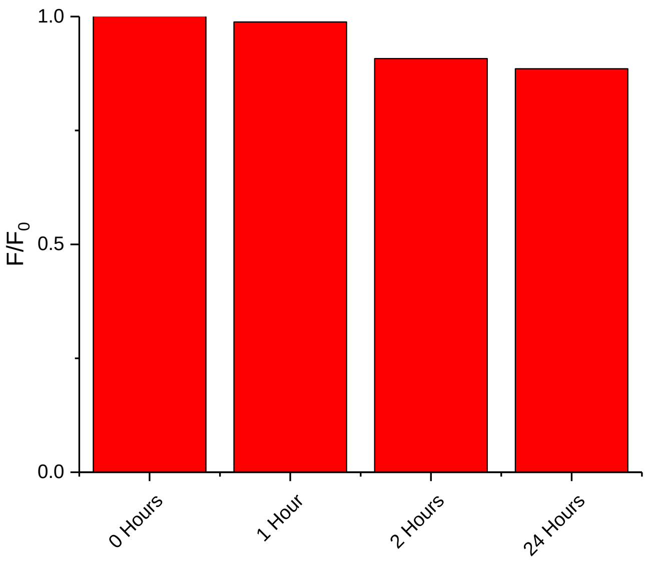

Figure S6-12. The stability profile of **4** (2 µM). The fluorescence intensity at 550 nm was continuously monitored at time intervals in pH 7.4, 1xPBS buffer–DMSO (9 : 1, v/v). Time points represent 0, 1, 2 and 24 hours.

S7 Cytotoxicity assays

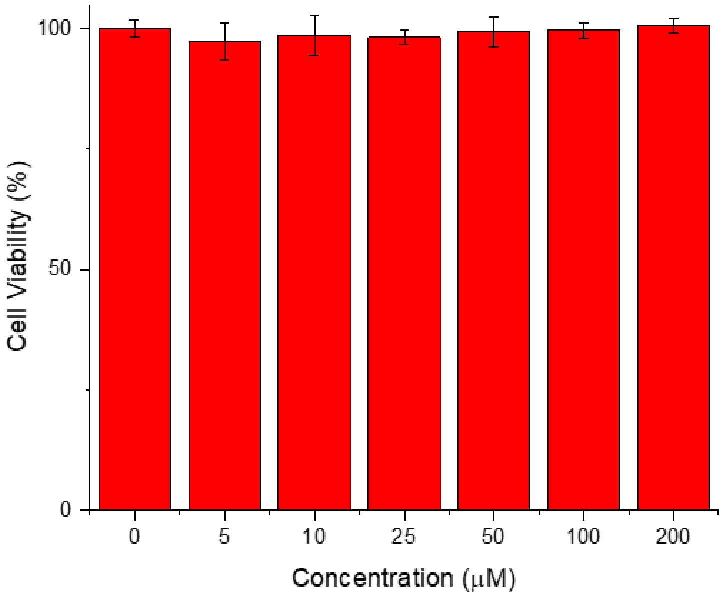

Figure S7-1. MTT cytotoxicity assay with different concentrations of **4** (0 - 200 μM) incubated in MCF-7 cells for 24 h.

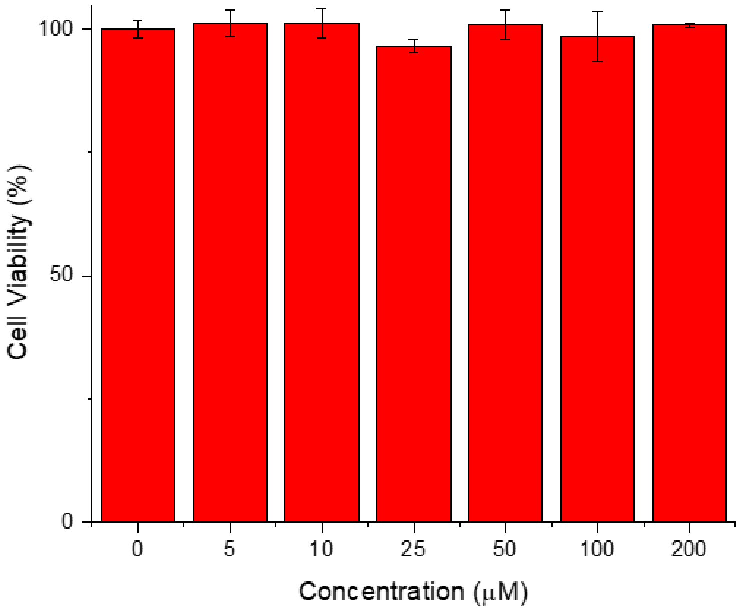

Figure S7-2. MTT cytotoxicity assay with different concentrations of **4·CO** (0 - 200 μM) incubated in MCF-7 cells for 24 h.

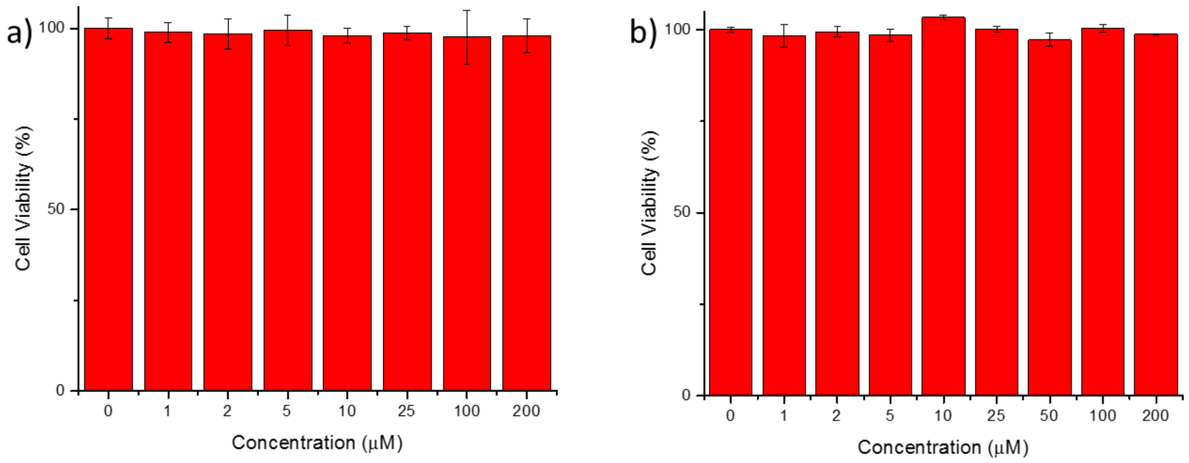

Figure S7-3. MTT cytotoxicity assay: a) different concentrations of **6** (0 - 200 μM) incubated in MCF-7 cells for 24 h b) different concentrations of **6·CO** (0 - 200 μM) incubated in MCF-7 cells for 24 h.

S8 Viscosity measurements

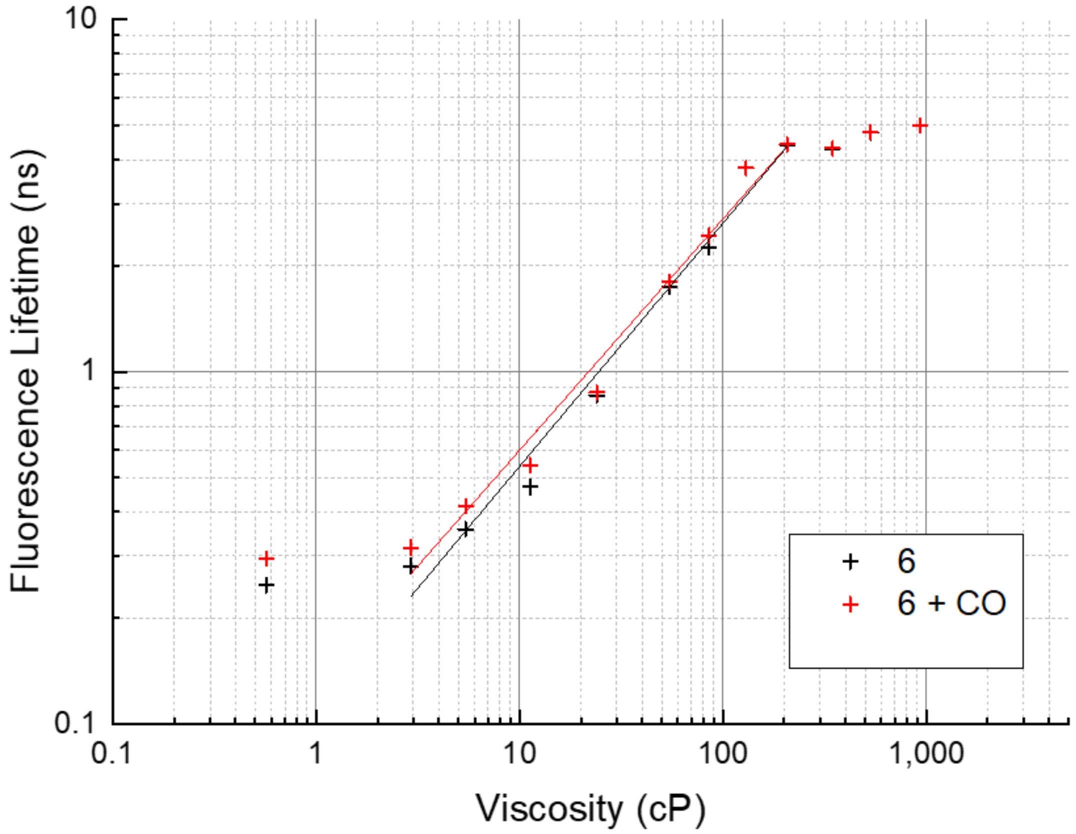

Figure S8-1. The logarithmic calibration plot of the dependence of average lifetime on viscosity, measured in methanol/glycerol mixtures of varying viscosities for **6** (5 µM) and **6·CO** (5 µM). The linear fit (blue) was performed in the linear range of the calibration plot according to the Förster-Hoffmann model. Equation: log (lifetime) = -10.16 + 0.79 * log(viscosity).

The values and the dynamic range of fluorescence lifetimes of BODIPY rotor attached to the ruthenium centres, above, matches closely with what was previously observed for a stand-alone organic BODIPY fluorophore of a very similar structure.[S8] This allows us to conclude that in both cases of complexes **6** and **6·CO**, where the lifetime response to viscosity was identical, irrespective of whether CO was attached to the metal centre of not, BODIPY ligand essentially acts as an independent organic fluorophore.

This conclusion is in line with the published work of Winter and co-workers, that reported ligand-dominated HOMO for some of the Ru vinyl complexes.[S9]

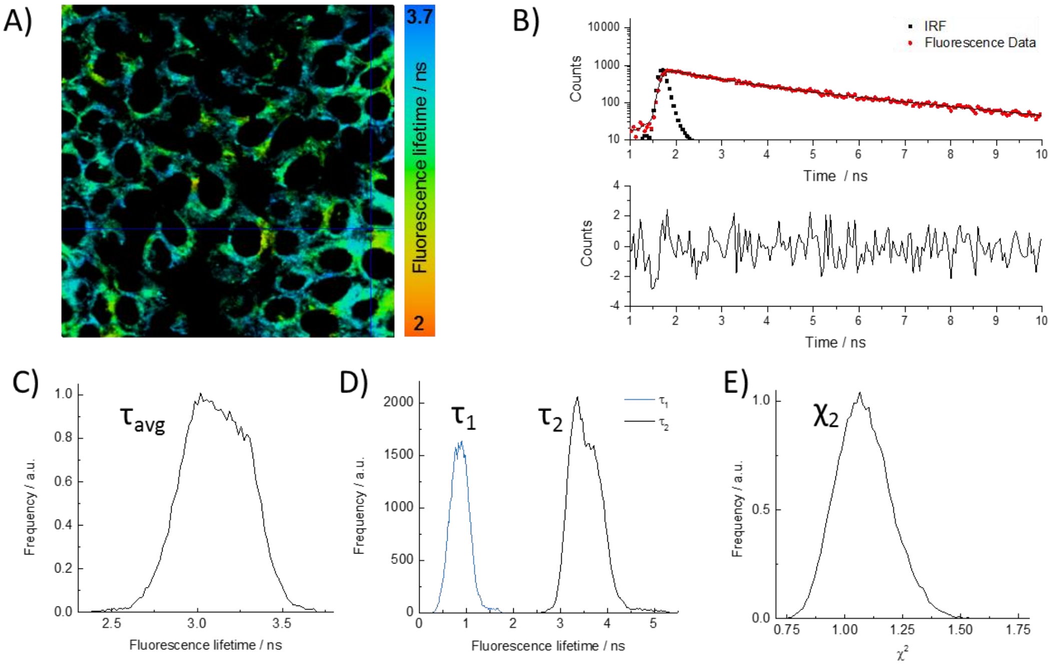

Figure S8-2. Bi-exponential fitting of fluorescence lifetime images. A) FLIM image of **6** in live MCF-7 cells with the average fluorescence lifetime colour coded; B) Fluorescence decay corresponding to a pixel in image A - the raw fluorescence decay was fitted using a bi-exponential function (red) and the instrument response function is shown in black with the match between the raw data and the fit assessed by the χ^2 'goodness of fit' parameter (in the case of the selected pixel, $\chi^2 = 1.05$) and a random distribution of residuals (shown underneath the decay); C) Distribution of average lifetime in image A; D) Distribution of the two fitted decay components in image A; E) Distribution of χ^2 in image A, showing the goodness of fit.

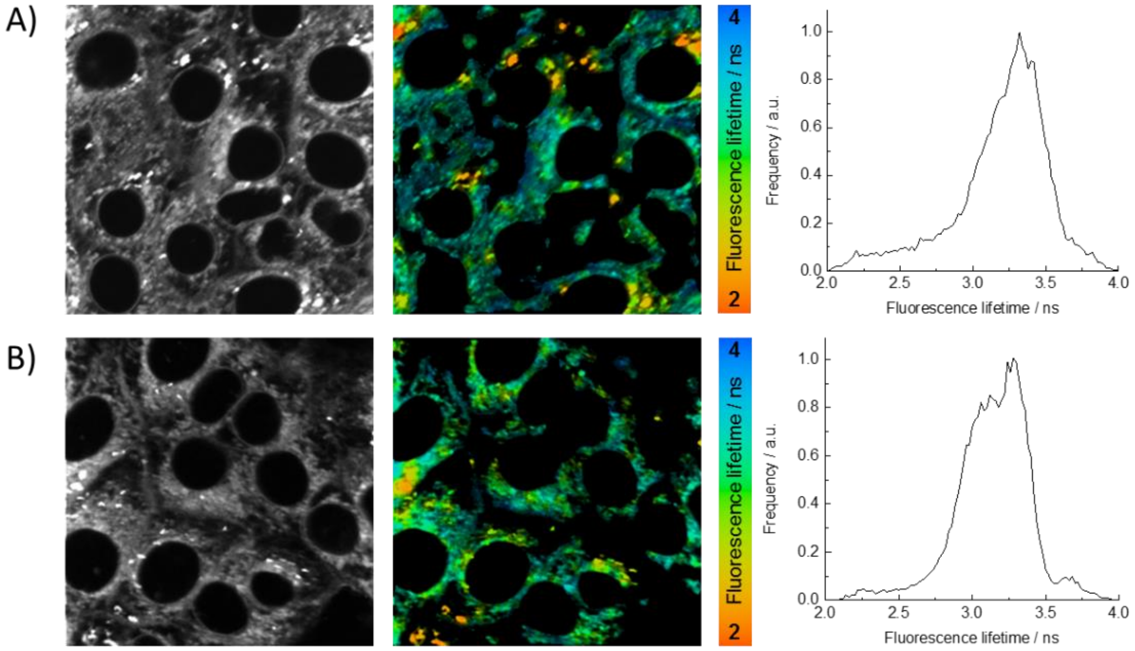

Figure S8-3. Brightfield and two-photon fluorescence lifetime image of live MCF-7 cells incubated with (a) 0 µM and (b) 100 µM CORM-2 for 30 min followed by **6** (10 µM) for 10 min; Excitation at 930 nm; Corresponding histograms are also provided, showing the distribution of fluorescence lifetimes.

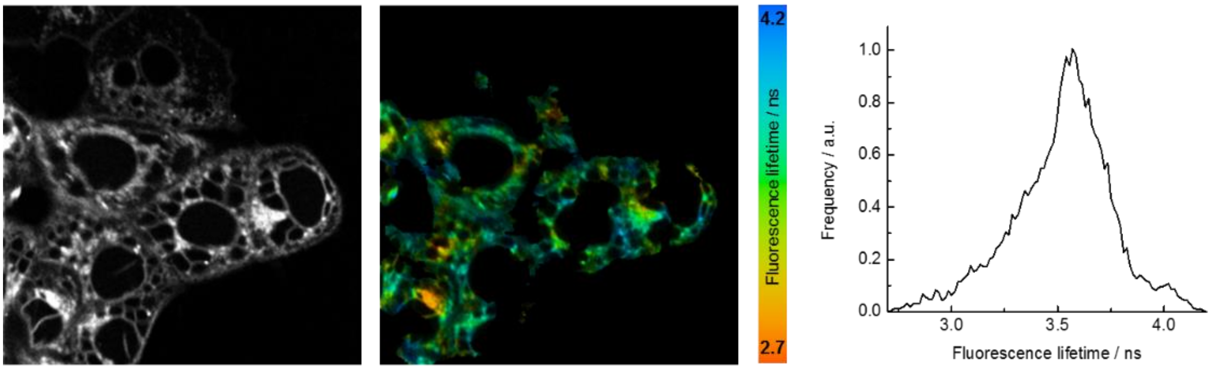

Figure S8-4. Brightfield fluorescence and FLIM of cells following incubation with 200 µM CORM and **6** (10 µM), with the corresponding histogram showing the distribution of fluorescence lifetime.

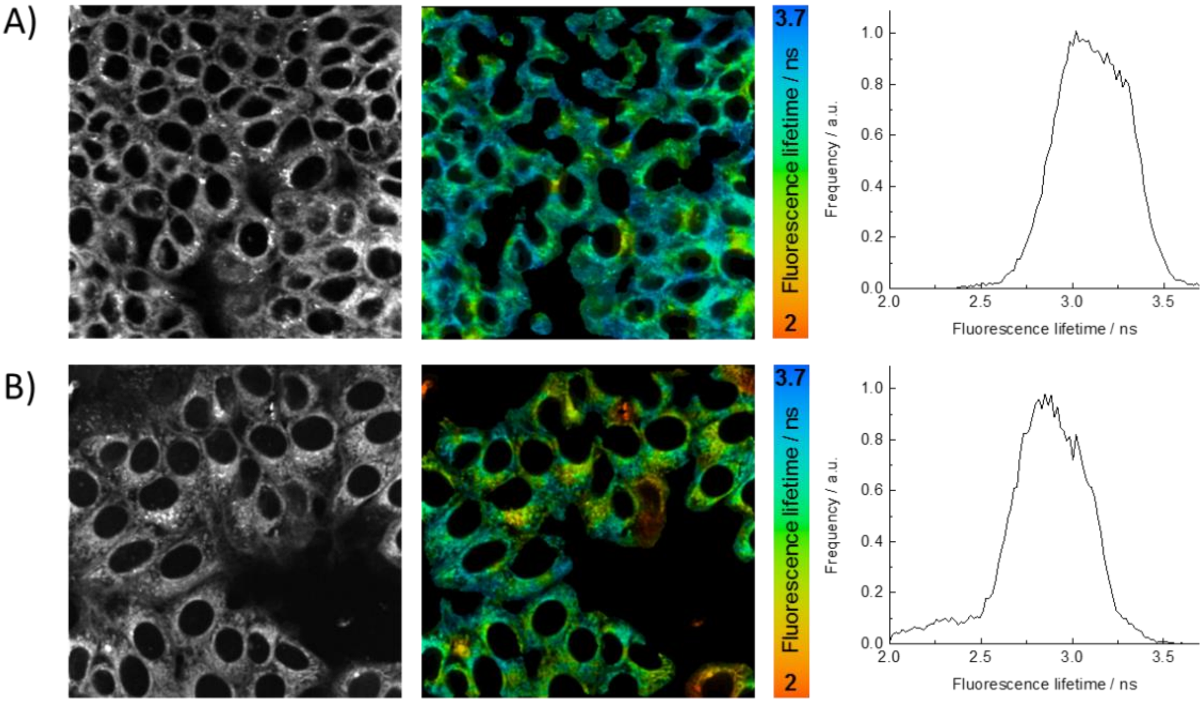

Figure S8-5. Brightfield and two-photon fluorescence lifetime images of live MCF-7 cells incubated with (a) 0 µM and (b) 100 µM hemin for 5 h followed by **6** (20 µM) for 10 min; Excitation at 930 nm; Corresponding histograms are also provided, showing the distribution of fluorescence lifetime.

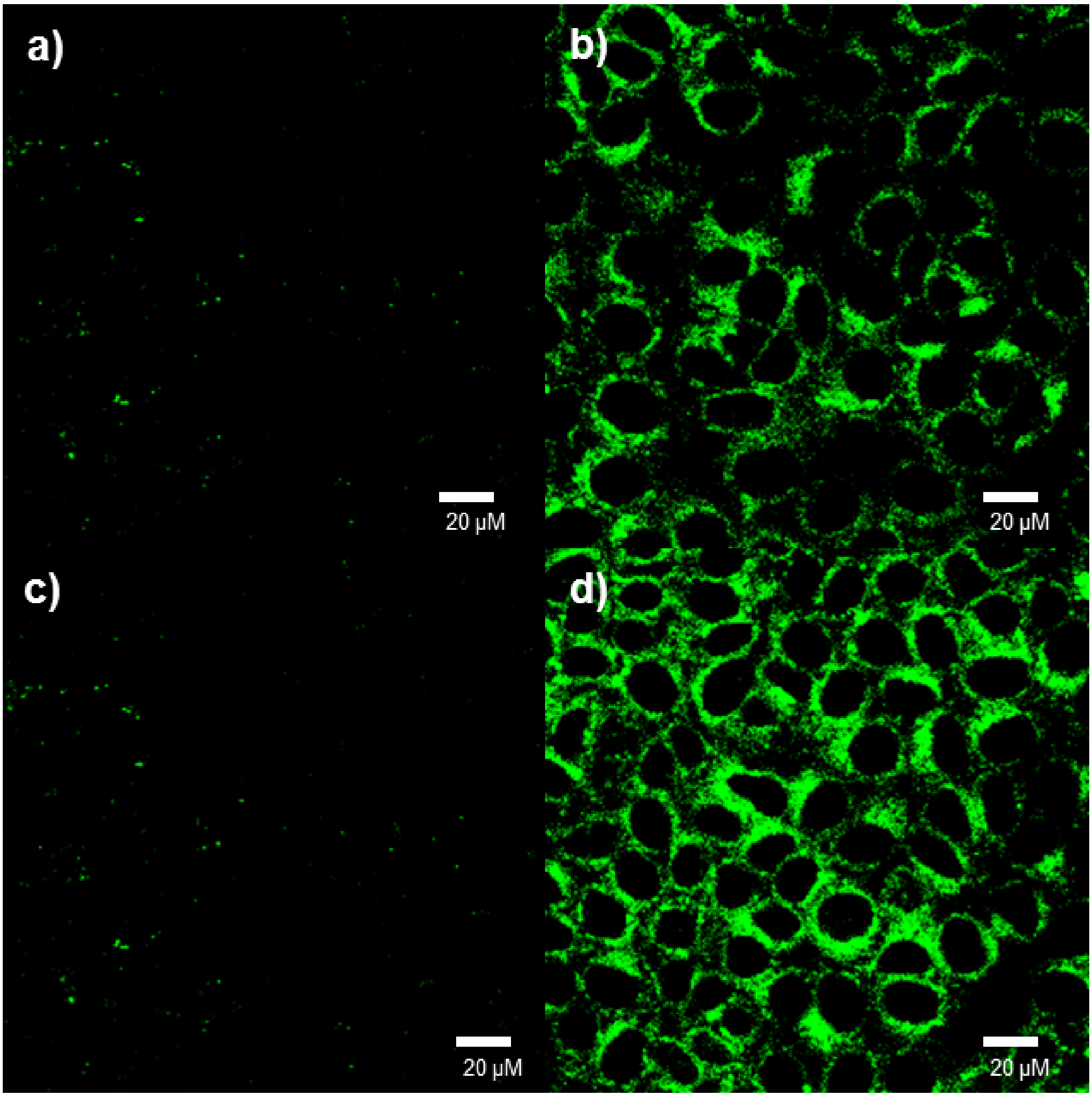

Figure S8-6. Confocal fluorescence images of of **6** in live MCF-7 cells incubated with (a) 0 µM, (b) 100 µM CORM-2 for 30 min followed by **6** (10 µM, λ_{ex} = 465, λ_{em} = 520 - 560 nm) for 30 min; Cells incubated with (c) 0 µM, (d) 100 µM hemin for 5 hours followed by **6** (10 µM) for 30 min.

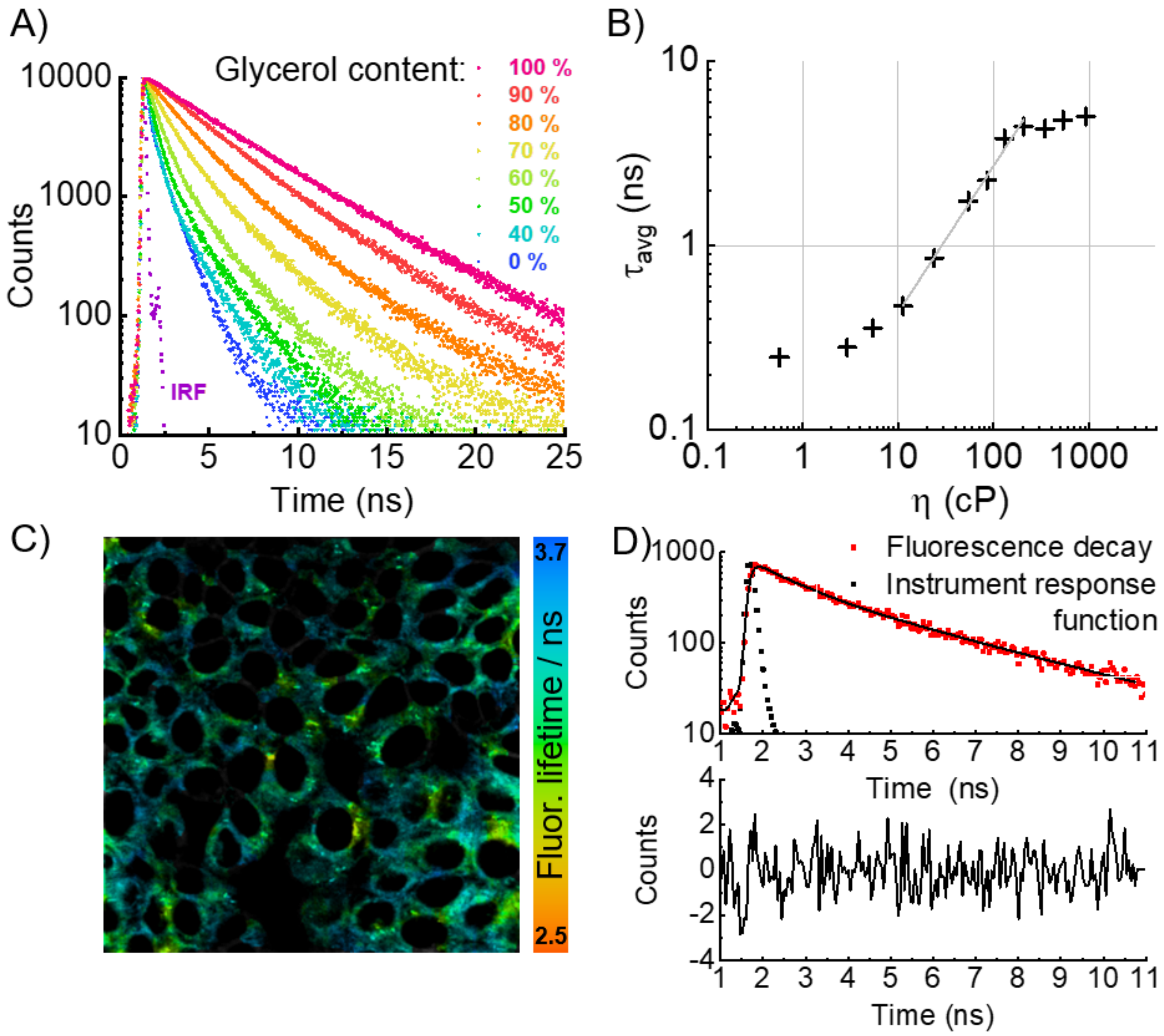

Figure S8-7. Fluorescence lifetime calibration of probe **6**. A) Time-resolved fluorescence decays recorded in methanol-glycerol mixtures of different viscosities; B) Double-logarithmic calibration plot of average lifetime as a function of viscosity. The linear fit (grey) according to Equation 1 was applicable between 10 and 200 cP; C) FLIM of probe 6 (20 μM) in live MCF-7 cells. D) Bi-exponential fit of a typical fluorescence decay from a pixel in C).

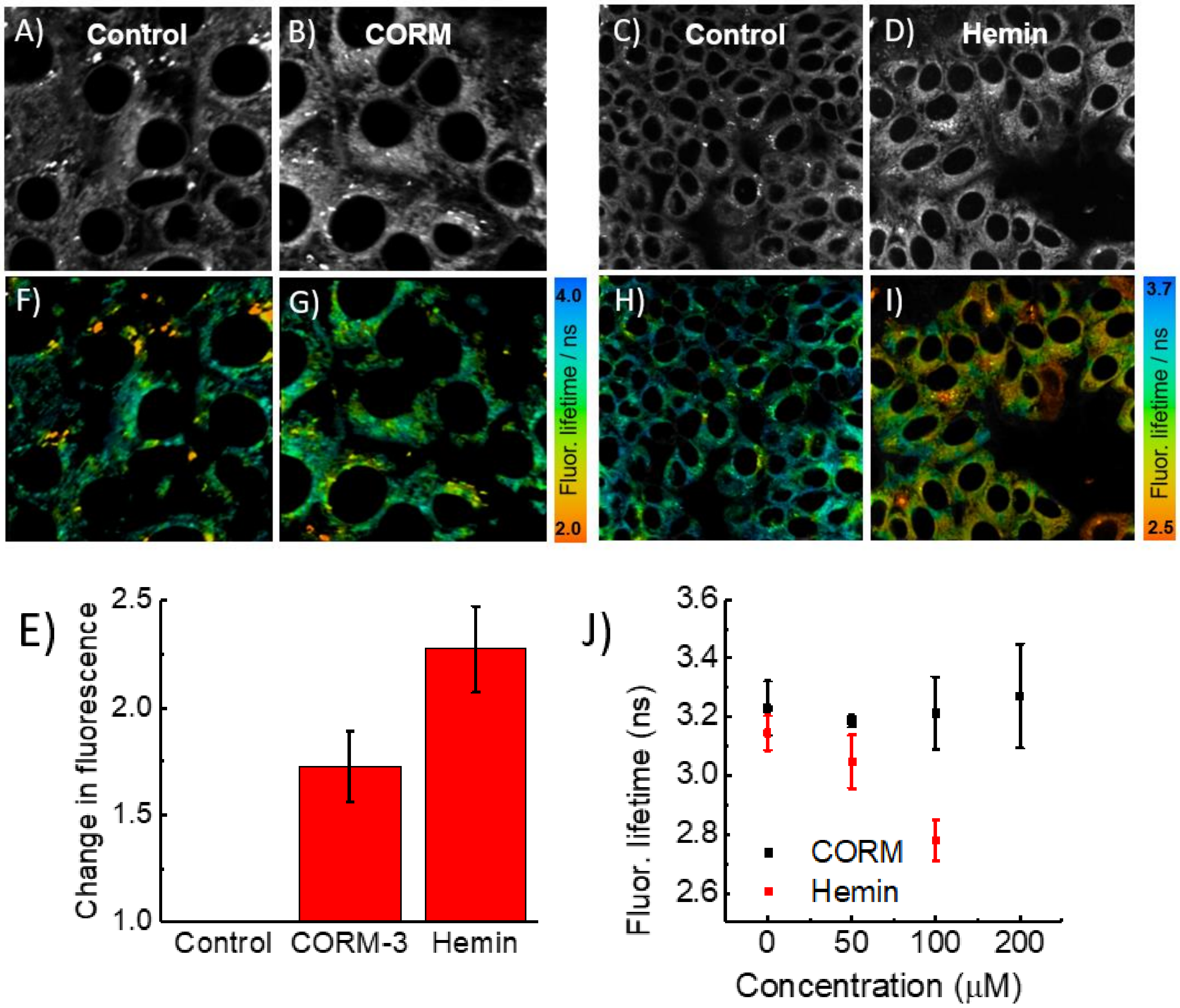

Figure S8-8. Fluorescence lifetime imaging of **6** (20 μM) in live MCF-7 cells showing intensity (A-E) and lifetime (F-J); A) and C) controls; B) incubated with 100 μM CORM-2; D) incubated with 100 μM hemin. E) Change in fluorescence intensity of cells with CORM-2 and hemin compared to controls. Underneath are the corresponding FLIM images; Data expressed as mean ±SEM of at least three independent experiments, and at least four FLIM images.

S9 Crystallography

S9.1 The X-ray crystal structure of 5·CO

Crystal data for **5·CO**: $C_{59}H_{50}BClF_2N_2O_2P_2Ru \cdot CH_2Cl_2$, *M* = 1151.20, triclinic, *P*-1 (no. 2), *a* = 10.1803(5), *b* = 12.2009(6), *c* = 23.9940(8) Å, α = 93.215(3), β = 100.327(3), γ = 111.007(5)°, *V* = 2713.5(2) Å^3, *Z* = 2, D_c = 1.409 g cm^{-3}, μ(Mo-Kα) = 0.548 mm^{-1}, *T* = 173 K, orange-brown blocks, Agilent Xcalibur 3 E diffractometer; 10742 independent measured reflections (R_{int} = 0.0222), F^2 refinement,[S12,S13] R_1(obs) = 0.0381, wR_2(all) = 0.0878, 9041 independent observed absorption-corrected reflections [$|F_o| > 4\sigma(|F_o|)$, completeness to θ_{full}(25.2°) = 98.6%], 675 parameters. CCDC 1964271.

The included dichloromethane solvent molecule in the structure of **5·CO** was found to be disordered. Two orientations were identified of *ca*. 74 and 26% occupancy, their geometries were optimized, the thermal parameters of adjacent atoms were restrained to be similar, and only the non-hydrogen atoms of the major occupancy orientation were refined anisotropically (those of the minor occupancy orientation were refined isotropically).

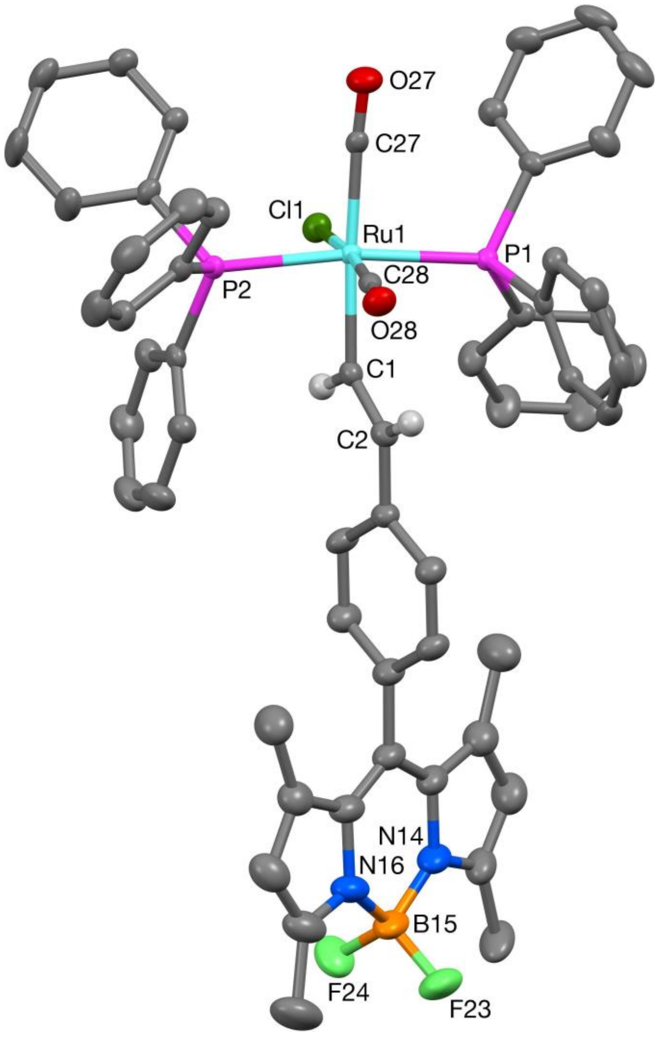

Figure S9-1. The crystal structure of **5·CO** (50% probability ellipsoids).

S10 References

S1 K. R. Laing, W. R. Roper, *J. Chem. Soc. A* **1970**, 2149-2153.

S2 A. Vázquez-Romero, N. Kielland, M. J. Arévalo, S. Preciado,R. J. Mellanby, Y. Feng, R. Lavilla, M. Vendrell, *J. Am. Chem. Soc.* **2013**, *43*, 16018-16021.

S3 M. Klein, U. Neugebauer, A. Gheisari, A. Malassa, T. M. A. Jazzazi, F. Froehlich, M. Westerhausen, M. Schmitt, J. Popp, *J. Phys. Chem. A.* **2014**, 118, 5381-5390.

S4 S. Erbas-Cakmak, F. Pir Cakmak, S. Demirel Topel, T. Bilal Uya, E. U. Akkaya, *Chem. Commun.* **2015**, 51, 12258-12261.

S5 A. Haefele, G. Ulrich, P. Retailleau, R. Ziessel, *Tetrahedron Lett.* **2008**, 49, 3716-3721.

S6 T. Bura, F. Nastasi, F. Puntoriero, S. Campagna, R. Ziessel, *Chem. Eur. J.* **2003**, 19, 8900-8912.

S7 S. L. Raut, J. D. Kimball, Z. Gryczynski, *Phys. Chem. Chem. Phys*. **2016**,18, 4535-4540.

S8 M. K. Kuimova, G. Yahioglu, J. A. Levitt, K. Suhling, *J. Am. Chem. Soc.* **2008**, *130*, 6672–6673.

S9 J. Maurer, M. Linseis, B. Sarkar, B. Schwederski, M. Niemeyer, W. Kaim, S. Záliš, C. Anson, M. Zabel, R. F. Winter, *J. Am. Chem. Soc*. **2008**, *130*, 259-268.

S10 X. Liu, W. Chi, Q. Qiao, S. V. Kokate, E. P. Cabrera, Z. Xu, X. Liu, Y.-T. Chang, *ACS Sens.* **2020**, *5*, 731–739.

S11 B. Woods, D. Döllerer, B. Aikman, M. N. Wenzel, E. J. Sayers, F. E. Kühn, A. T. Jones A. Casini. *J. Inorg. Biochem.* 2019, **199**, 110781.

S12 SHELXTL v5.1, Bruker AXS, Madison, WI, 1998.

S13 SHELX-2013, G. M. Sheldrick, *Acta Cryst.* **2015**, *C71*, 3-8.
